# De novo design of protein structure and function with RFdiffusion

**DOI:** 10.1038/s41586-023-06415-8

**Published:** 2023-07-11

**Authors:** Joseph L. Watson, David Juergens, Nathaniel R. Bennett, Brian L. Trippe, Jason Yim, Helen E. Eisenach, Woody Ahern, Andrew J. Borst, Robert J. Ragotte, Lukas F. Milles, Basile I. M. Wicky, Nikita Hanikel, Samuel J. Pellock, Alexis Courbet, William Sheffler, Jue Wang, Preetham Venkatesh, Isaac Sappington, Susana Vázquez Torres, Anna Lauko, Valentin De Bortoli, Emile Mathieu, Sergey Ovchinnikov, Regina Barzilay, Tommi S. Jaakkola, Frank DiMaio, Minkyung Baek, David Baker

**Affiliations:** 1 Department of Biochemistry, University of Washington, Seattle, WA USA; 2 Institute for Protein Design, University of Washington, Seattle, WA USA; 3 Graduate Program in Molecular Engineering, University of Washington, Seattle, WA USA; 4 Columbia University, Department of Statistics, New York, NY USA; 5 Irving Institute for Cancer Dynamics, Columbia University, New York, NY USA; 6 Massachusetts Institute of Technology, Cambridge, MA USA; 7 Paul G. Allen School of Computer Science and Engineering, University of Washington, Seattle, WA USA; 8 National Centre for Scientific Research, École Normale Supérieure rue d’Ulm, Paris, France; 9 Graduate Program in Biological Physics, Structure and Design, University of Washington, Seattle, WA USA; 10 Department of Engineering, University of Cambridge, Cambridge, UK; 11 Faculty of Applied Sciences, Harvard University, Cambridge, MA USA; 12 John Harvard Distinguished Science Fellowship, Harvard University, Cambridge, MA USA; 13 School of Biological Sciences, Seoul National University, Seoul, Republic of Korea; 14 Howard Hughes Medical Institute, University of Washington, Seattle, WA USA

**Keywords:** Protein design, Proteins, Machine learning

## Abstract

There has been considerable recent progress in designing new proteins using deep-learning methods^1–9^. Despite this progress, a general deep-learning framework for protein design that enables solution of a wide range of design challenges, including de novo binder design and design of higher-order symmetric architectures, has yet to be described. Diffusion models^10,11^ have had considerable success in image and language generative modelling but limited success when applied to protein modelling, probably due to the complexity of protein backbone geometry and sequence–structure relationships. Here we show that by fine-tuning the RoseTTAFold structure prediction network on protein structure denoising tasks, we obtain a generative model of protein backbones that achieves outstanding performance on unconditional and topology-constrained protein monomer design, protein binder design, symmetric oligomer design, enzyme active site scaffolding and symmetric motif scaffolding for therapeutic and metal-binding protein design. We demonstrate the power and generality of the method, called RoseTTAFold diffusion (RFdiffusion), by experimentally characterizing the structures and functions of hundreds of designed symmetric assemblies, metal-binding proteins and protein binders. The accuracy of RFdiffusion is confirmed by the cryogenic electron microscopy structure of a designed binder in complex with influenza haemagglutinin that is nearly identical to the design model. In a manner analogous to networks that produce images from user-specified inputs, RFdiffusion enables the design of diverse functional proteins from simple molecular specifications.

## Abstract (web summary)

Fine-tuning the RoseTTAFold structure prediction network on protein structure denoising tasks yields a generative model for protein design that achieves outstanding performance on a wide range of protein structure and function design challenges.

## Main

De novo protein design seeks to generate proteins with specified structural and/or functional properties, for example, making a binding interaction with a given target^12^, folding into a particular topology^13^ or containing a catalytic site^4^. Denoising diffusion probabilistic models (DDPMs), a powerful class of machine learning models recently demonstrated to generate new photorealistic images in response to text prompts^14,15^, have several properties well suited to protein design. First, DDPMs generate highly diverse outputs, as they are trained to denoise data (for instance, images or text) that have been corrupted with Gaussian noise. By learning to stochastically reverse this corruption, diverse outputs closely resembling the training data are generated. Second, DDPMs can be guided at each step of the iterative generation process towards specific design objectives through provision of conditioning information. Third, for almost all protein design applications it is necessary to explicitly model three-dimensional (3D) structures; rotationally equivariant DDPMs can do this in a global representation frame independent manner. Recent work has adapted DDPMs for protein monomer design by conditioning on small protein ‘motifs’^5,9^ or on secondary structure and block-adjacency (‘fold’) information^8^. Although promising, these attempts have shown limited success in generating sequences that fold to the intended structures in silico^5,16^, probably due to the limited ability of the denoising networks to generate realistic protein backbones, and have not been tested experimentally.

**Fig. 1:**
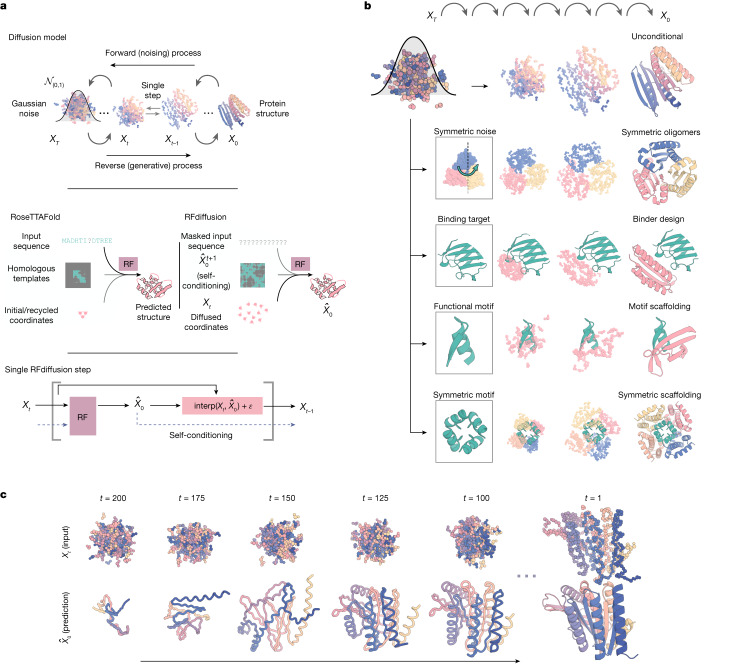
Protein design using RFdiffusion. **a**, Diffusion models for proteins are trained to recover corrupted (noised) protein structures and to generate new structures by reversing the corruption process through iterative denoising of initially random noise *X*_*T*_ into a realistic structure *X*_0_ (top panel). The RF structure prediction network (middle panel, left side) is fine-tuned with minimal architectural changes into RFdiffusion (middle panel, right side); the denoising network of a DDPM is also shown. In RF, the primary input to the model is the sequence. In RFdiffusion, the primary input is diffused residue frames (coordinates and orientations). In both cases, the model predicts final 3D coordinates (denoted ${\hat{X}}_{0}$ in RFdiffusion). The bottom panel shows that in RFdiffusion, the model receives its previous prediction as a template input (‘self-conditioning’, Supplementary Methods). At each timestep *t* of a trajectory (typically 200 steps), RFdiffusion takes ${\hat{X}}_{0}^{t+1}$ from the previous step and *X*_*t*_ and then predicts an updated *X*_0_ structure (${\hat{X}}_{0}^{t}$). The next coordinate input to the model (${X}_{t-1}$) is generated by a noisy interpolation (interp) towards ${\hat{X}}_{0}^{t}$. **b**, RFdiffusion is broadly applicable for protein design. RFdiffusion generates protein structures either without further input (top row) or by conditioning on (top to bottom): symmetry specifications; binding targets; protein functional motifs or symmetric functional motifs. In each case random noise, along with conditioning information, is input to RFdiffusion, which iteratively refines that noise until a final protein structure is designed. **c**, An example of an unconditional design trajectory for a 300-residue chain, depicting the input to the model (*X*_*t*_) and the corresponding ${\hat{X}}_{0}$ prediction. At early timesteps (high *t*), ${\hat{X}}_{0}$ bears little resemblance to a protein but is gradually refined into a realistic protein structure.

We reasoned that improved diffusion models for protein design could be developed by taking advantage of the deep understanding of protein structure implicit in powerful structure prediction methods such as AlphaFold2 (ref. ^17^) (AF2) and RoseTTAFold^18^ (RF). RF has properties well suited for use in a protein design DDPM (Fig. 1a): it generates protein structures with high precision, operates on a rigid-frame representation of residues with rotational equivariance and has an architecture enabling conditioning on design specifications at the individual residue, inter-residue distance and orientation, and 3D coordinate levels. In previous work, we fine-tuned RF to complete protein backbones around input functional motifs in a single step (RF_joint_ Inpainting^4^). Experimental characterization showed that the method can scaffold a wide range of protein functional motifs with atomic accuracy^19^, but the approach fails on minimalist site descriptions that do not sufficiently constrain the overall fold and, because it is deterministic, can produce only a limited diversity of designs for a given problem. We reasoned that by fine-tuning RF as the denoising network in a generative diffusion model instead, we could overcome both problems: because the starting point is random noise, each denoising trajectory yields a different solution, and because structure is built up progressively through many denoising iterations, little to no starting structural information should be required. In this study, we used an updated version of RF^18^ as the basis for the denoising network architecture (Supplementary Methods), but other equivariant structure prediction networks (AF2 (ref. ^17^), OmegaFold^20^, ESMFold^21^) could in principle be substituted into an analogous DDPM.

We construct a RF-based diffusion model, RFdiffusion, using the RF frame representation that comprises a Cα coordinate and N-Cα-C rigid orientation for each residue. We generate training inputs by noising structures sampled from the Protein Data Bank (PDB) for up to 200 steps^22^. For translations, we perturb Cα coordinates with 3D Gaussian noise. For residue orientations, we use Brownian motion on the manifold of rotation matrices (building on refs. ^23,24^). To enable RFdiffusion to learn to reverse each step of the noising process, we train the model by minimizing a mean-squared error (m.s.e.) loss between frame predictions and the true protein structure (without alignment), averaged across all residues (Supplementary Methods). This loss drives denoising trajectories to match the data distribution at each timestep and hence to converge on structures of designable protein backbones (Extended Data Fig. 2a). The m.s.e. contrasts to the loss used in RF structure prediction training (frame aligned point error or FAPE) in that, unlike FAPE, m.s.e. loss is not invariant to the global reference frame and therefore promotes continuity of the global coordinate frame between timesteps (Supplementary Methods).

To generate a new protein backbone, we first initialize random residue frames and RFdiffusion makes a denoised prediction. Each residue frame is updated by taking a step in the direction of this prediction with some noise added to generate the input to the next step. The nature of the noise added and the size of this reverse step is chosen such that the denoising process matches the distribution of the noising process (Supplementary Methods and Extended Data Fig. 2a). RFdiffusion initially seeks to match the full breadth of possible protein structures compatible with the purely random frames with which it is initialized, and hence the denoised structures do not initially seem protein-like (Fig. 1c, left). However, through many such steps, the breadth of possible protein structures from which the input could have arisen narrows and RFdiffusion predictions come to closely resemble protein structures (Fig. 1c, right). We use the ProteinMPNN network^1^ to subsequently design sequences encoding these structures, typically sampling eight sequences per design in line with previous work^5,16^ (but see Supplementary Fig. 2a). We also considered simultaneously designing structure and sequence within RFdiffusion, but given the excellent performance of combining ProteinMPNN with the diffusion of structure alone, we did not extensively explore this possibility.

Figure 1a highlights the similarities between RF structure prediction and an RFdiffusion denoising step: in both cases, the networks transform coordinates into a predicted structure, conditioned on inputs to the model. In RF, sequence is the primary input, with extra structural information provided as templates and initial coordinates to the model. In RFdiffusion, the primary input is the noised coordinates from the previous step. For specific design tasks, a range of auxiliary conditioning information, including partial sequence, fold information or fixed functional-motif coordinates can be provided (Fig. 1b and Supplementary Methods).

**Fig. 2:**
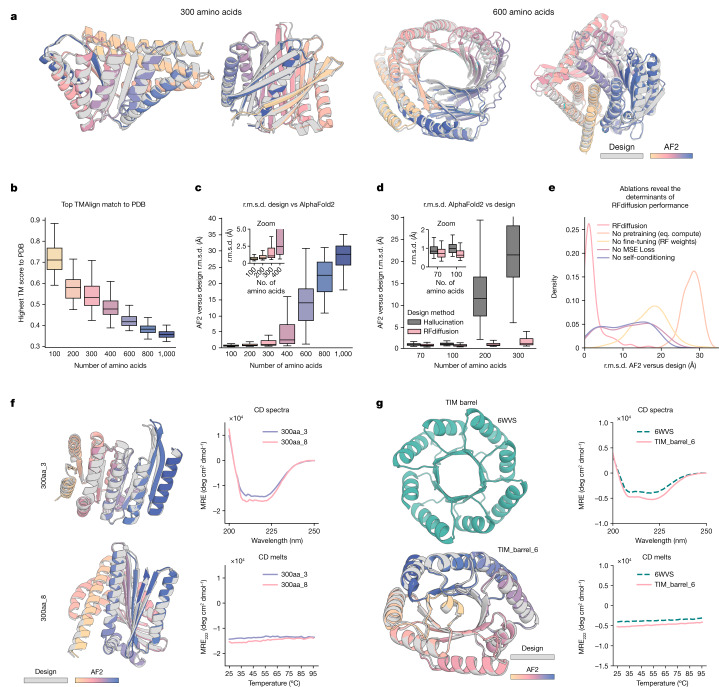
Outstanding performance of RFdiffusion for monomer generation. **a**, RFdiffusion can generate new monomeric proteins of different lengths (left 300, right 600) with no conditioning information. Grey, design model; colours, AF2 prediction. r.m.s.d. AF2 versus design (Å), left to right: 0.90, 0.98, 1.15, 1.67. **b**, Unconditional designs from RFdiffusion are new and not present in the training set as quantified by highest TM-score to the PDB; the divergence from previously known structures increases with length. **c**, Unconditional samples are closely repredicted by AF2 up to about 400 amino acids. **d**, RFdiffusion significantly outperforms Hallucination (with RF) at unconditional monomer generation (two-proportion *z*-test of in silico success: *n* = 400 designs per condition, *z* = 9.5, *P* = 1.6 × 10^−21^). Although Hallucination successfully generates designs up to 100 amino acids in length, in silico success rates rapidly deteriorate beyond this length. **e**, Ablating pretraining (by starting from untrained RF), RFdiffusion fine-tuning (that is, using original RF structure prediction weights as the denoiser), self-conditioning or m.s.e. losses (by training with FAPE) each notably decrease the performance of RFdiffusion. r.m.s.d. between design and AF2 is shown, for the unconditional generation of 300 amino acid proteins (Supplementary Methods). **f**, Two example 300 amino acid proteins that expressed as soluble monomers. Designs (grey) overlaid with AF2 predictions (colours) are shown on the left, alongside circular dichroism (CD) spectra (top) and melt curves (bottom) on the right. The designs are highly thermostable. **g**, RFdiffusion can condition on fold information. An example TIM barrel is shown (bottom left), conditioned on the secondary structure and block adjacency of a previously designed TIM barrel, PDB 6WVS (top left). Designs have very similar circular dichroism spectra to PDB 6WVS (top right) and are highly thermostable (bottom right). See also Extended Data Fig. 3 for further traces. Boxplots represent median ± interquartile range; tails are minimum and maximum excluding outliers (±1.5× interquartile range).

**Extended Data Fig. 1:**
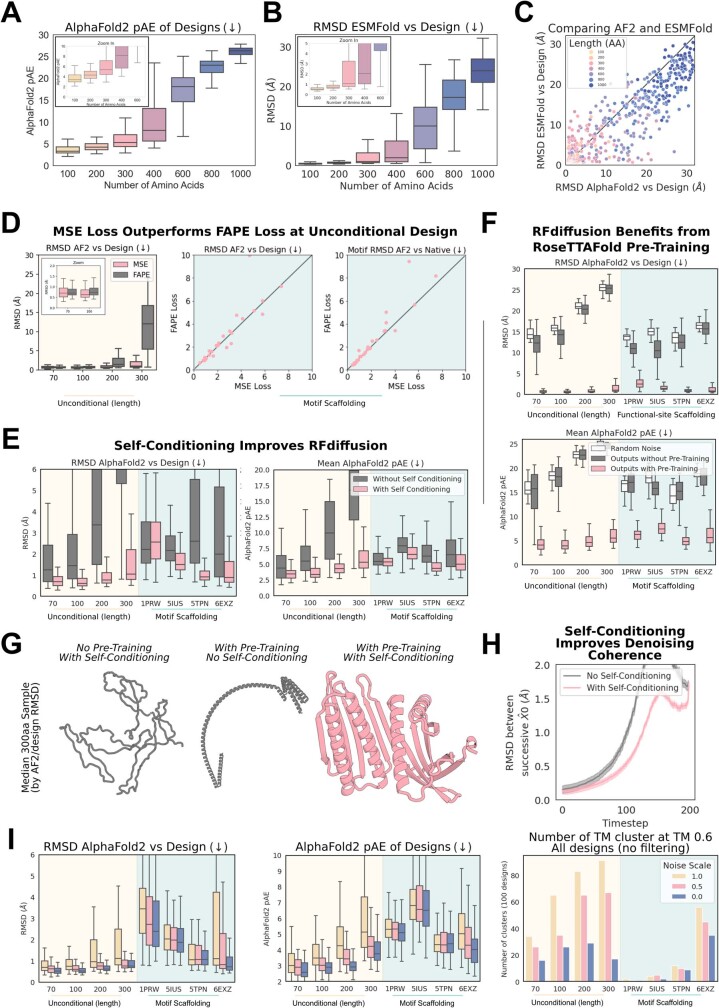
Training ablations reveal determinants of RFdiffusion success. **A**–**C**) RFdiffusion can generate high quality large unconditional monomers. Designs are routinely accurately recapitulated by AF2 (see also Fig. 2c), with high confidence (**A**) for proteins up to approximately 400 amino acids in length. **B**) Further orthogonal validation of designs by ESMFold. **C**) Recapitulation of the design structure is often better with ESMFold compared with AF2. For each backbone, the best of 8 ProteinMPNN sequences is plotted, with points therefore paired by backbone rather than sequence. **D**) Comparing RFdiffusion trained with MSE loss on Cα atoms and N-Cα-C backbone frames (Methods 2.5), rather than with FAPE loss^8,17^. The MSE loss is not invariant to the global coordinate frame, unlike FAPE loss, and is required for good performance at unconditional generation (left, two-proportion z-test of in silico success rate, *n* = 400 designs per condition*, z* = *4.1, p* = *4.1e-5*). For motif scaffolding problems, where the ‘motif’ provides a means to align the global coordinate frame between timesteps, FAPE loss performs approximately as well as MSE loss, suggesting the L2 nature of MSE loss (as opposed to the L1 loss in FAPE) is not empirically critical for performance. **E**) Allowing the model to condition on its X_0_ prediction at the previous timestep (see Supplementary Methods 2.4) improves designs. Designs with self-conditioning (pink) have improved recapitulation by AF2 (left) and better AF2 confidence in the prediction (right). Two-proportion z-test of in silico success rate, *n* = 800 designs per condition *z* = *11.4, p* = *6.1e-30*. **F**) RFdiffusion leverages the representations learned during RF pre-training. RFdiffusion fine-tuned from pre-trained RF (pink) comprehensively outperforms a model trained for an equivalent amount of time, from untrained weights (gray). For context, sequences generated by ProteinMPNN on these output backbones are little better than sampling ProteinMPNN sequences from random Gaussian-sampled coordinates (white). Two-proportion z-test of in silico success rate, pre-training vs without pre-training (or vs random noise; both have zero success rate), *n* = 800 designs per condition, *z* = *23.0, p* = *3.1e-117*. Note that the data in pink in **D**–**F** is the same data, reproduced in each plot for clarity. **G**) The median (by AF2 r.m.s.d. vs design) 300 amino acid unconditional sample highlighting the importance of self-conditioning and pre-training. Without pre-training (at least when trained with equivalent compute), RFdiffusion outputs bear little resemblance to proteins (gray, left). Without self-conditioning, outputs show characteristic protein secondary structures, but lack core-packing and ideality (gray, middle). With pre-training and self-conditioning, proteins are diverse and well-packed (pink, right). **H**) Greater coherence during unconditional denoising may partly explain the effect of self-conditioning. Successive X_0_ predictions are more similar when the model can self-condition (lower r.m.s.d. between X_0_ predictions, pink curve). Data are aggregated from unconditional design trajectories of 100, 200 and 300 residues. **I**) During the reverse (generation) process, the noise added at each step can be scaled (reduced). Reducing the noise scale improves the *in silico* design success rates (left, middle; two-proportion z-test of *in silico* success rate*, n* = 800 designs per condition, 0 vs 0.5: *z* = *1.7, p* = *0.09*, 0 vs 1: *z* = *6.5, p* = *6.8e-11*; 0.5 vs 1: *z* = *4.8, p* = *1.4e-6*). This comes at the expense of diversity, with the number of unique clusters at a TM-score cutoff of 0.6 reduced when noise is reduced (right). Note throughout this figure the 6EXZ_long benchmarking problem is abbreviated to 6EXZ for brevity. Boxplots represent median±IQR; tails: min/max excluding outliers (±1.5xIQR).

We explored two different strategies for training RFdiffusion: (1) in a manner akin to ‘canonical’ diffusion models, with predictions at each timestep independent of predictions at previous timesteps (as in previous work^5,8,9,16^), and (2) with self-conditioning^25^, in which the model can condition on previous predictions between timesteps (Fig. 1a, bottom row and Supplementary Methods). The latter strategy was inspired by the success of ‘recycling’ in AF2, which is also central to the more recent RF model used here (Supplementary Methods). Self-conditioning within RFdiffusion notably improved performance on in silico benchmarks encompassing both conditional and unconditional protein design tasks (Fig. 2e and Extended Data Fig. 1e). Increased coherence of predictions within self-conditioned trajectories may, at least in part, explain these performance increases (Extended Data Fig. 1h). Fine-tuning RFdiffusion from pretrained RF weights was far more successful than training for an equivalent length of time from untrained weights (Extended Data Fig. 1f,g, also Supplementary Fig. 1) and the m.s.e. loss was also crucial for unconditional generation (Extended Data Fig. 1d). For all in silico benchmarks in this paper, we use the AF2 structure prediction network^17^ for validation and define an in silico ‘success’ as an RFdiffusion output for which the AF2 structure predicted from a single sequence is (1) of high confidence (mean predicted aligned error (pAE), less than five), (2) globally within a 2 Å backbone root mean-squared deviation (r.m.s.d.) of the designed structure and (3) within 1 Å backbone r.m.s.d. on any scaffolded functional site (Supplementary Methods). This measure of in silico success has been found to correlate with experimental success^4,7,26^ and is significantly more stringent than template modelling (TM)-score-based metrics used elsewhere^5,16,27–29^ (Supplementary Fig. 2c,d).

## Unconditional protein monomer generation

**Extended Data Fig. 2:**
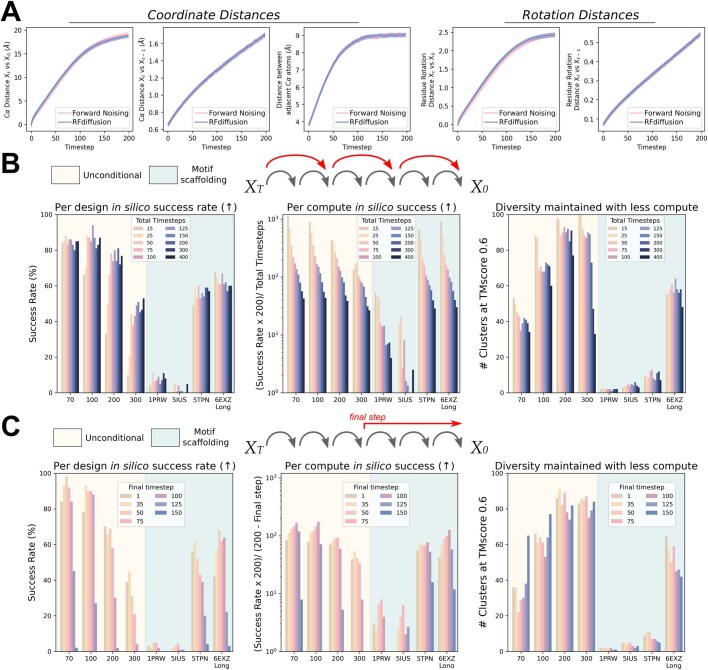
RFdiffusion learns the distribution of the denoising process, and inference efficiency can be improved. **A**) Analysis of simulated forward (noising) and reverse (denoising) trajectories shows that the distribution of Cα coordinates and residue orientations closely match, demonstrating that RFdiffusion has learned the distribution of the denoising process as desired. Left to right: i) average distance between a Cα coordinate at X_t_ and its position in X_0_; ii) average distance between a Cα coordinate at X_t_ and X_t-1_; iii) average distance between adjacent Cα coordinates at X_t_; iv) average rotation distance between a residue orientation at X_t_ and X_0_; v) average rotation distance between a residue orientation at X_t_ and X_t-1_. **B**-**C**) While RFdiffusion is trained to generate samples over 200 timesteps, in many cases, trajectories can be shortened to improve computational efficiency. **B**) Larger steps can be taken between timesteps at inference. Decreasing the number of timesteps speeds up inference, and often does not decrease in silico success rates (left) (for example, on an NVIDIA A4000 GPU, 100 amino acid designs can be generated with 15 steps, in ~11s, with an in silico success rate of over 60%). When normalized for compute budget (center) it is often much more efficient to run more trajectories with fewer timesteps. This can be done without loss of diversity in samples (right). For harder problems (e.g. unconditional 300 amino acids), one must strike an intermediate number of total timesteps (e.g., T = 50) for optimal compute efficiency. Note that for all other analyses in the paper, 200 inference steps were used, in line with how RFdiffusion is trained. **C**) An alternative to taking larger steps is to stop trajectories early (possible because RFdiffusion predicts X_0_ at every timestep). In many cases, trajectories can be stopped at timestep 50–75 with little effect on the final in silico success rate of designs (left), and when normalized by compute budget (center), success rates per unit time are typically higher generating more designs with early-stopping. Again, this can be done without a significant loss in diversity (right).

**Extended Data Fig. 3:**
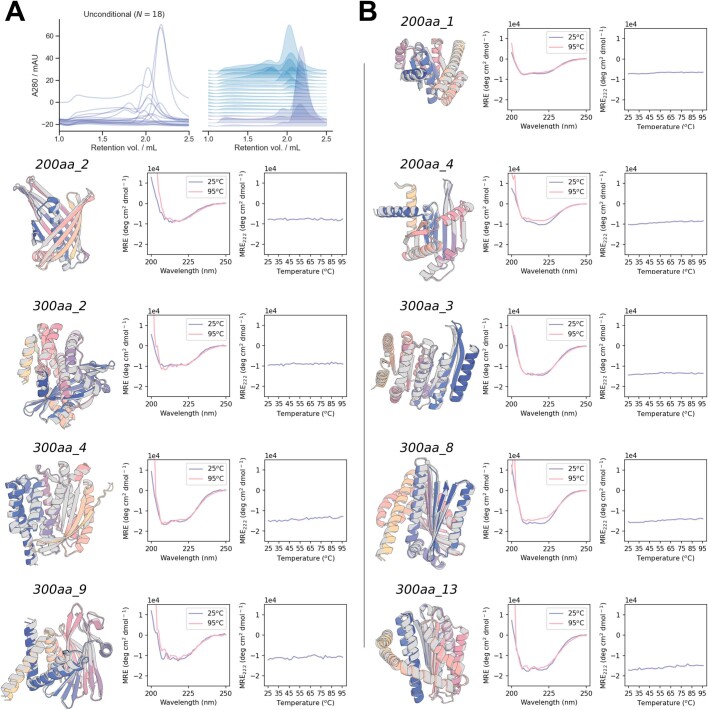
Unconditionally-generated designs are folded and thermostable. **A**) Four 200 amino acid and fourteen 300 amino acid proteins were tested for expression and stability. 9/18 designs expressed, with a major peak at the expected elution volume. Blue: 300 amino acid proteins; Purple: 200 amino acid proteins. **B**) Colored AF2 predictions overlaid on gray design models (left), circular dichroism spectra at 25 °C (blue) and 95 °C (pink) (middle) and circular dichroism melt curves (right) for all 9 designs passing expression thresholds. In all cases, proteins remain well folded even at 95 °C. Note that data on *300aa_3* and *300aa_8* are duplicated from Fig. 2f, reproduced here for clarity.

As shown in Fig. 2a–c and Supplementary Fig. 3c,d, starting from random noise, RFdiffusion can readily generate elaborate protein structures with little overall structural similarity to structures seen during training, indicating considerable generalization beyond the PDB (see Supplementary Table 1 for a comparison of all designs in the paper to the PDB). The designs are diverse (Supplementary Fig. 3a), spanning a wide range of alpha, beta and mixed alpha–beta topologies, with AF2 and ESMFold (Fig. 2c, Extended Data Fig. 1b,c and Supplementary Fig. 2b) predictions very close to the design structure models for de novo designs with as many as 600 residues. RFdiffusion generates plausible structures for even very large proteins, but these are difficult to validate in silico as they are probably generally beyond the single sequence prediction capabilities of AF2 and ESMFold. The quality and diversity of designs that are sampled are inherent to the model, and do not depend on any auxiliary conditioning input (for example, secondary structure information^8^). We experimentally characterized six of the 300 amino acid designs and three of the 200 amino acid designs, and found that they have circular dichroism spectra consistent with the mixed alpha–beta topologies of the designs and are extremely thermostable (Extended Data Fig. 3). Physics-based protein design methodologies have struggled in unconstrained generation of diverse protein monomers because of the difficulty of sampling on the very large and rugged conformational landscape^30^, and overcoming this limitation has been a primary test of deep-learning based protein design approaches^5,6,8,16,27,31^. RFdiffusion strongly outperforms (based on the AF2 success metric described above) Hallucination with RF, an experimentally validated method using Monte Carlo search or gradient descent to identify sequences predicted to fold into stable structures (Fig. 2d). RFdiffusion generation is also more compute efficient than unconstrained Hallucination with RF, and efficiency can be greatly improved by taking larger steps at inference time and by truncating trajectories early, which is possible because RF predicts the final structure at each timestep (Extended Data Fig. 2b,c). For example, a 100-residue protein can be generated in as little as 11 s on an NVIDIA RTX A4000 Graphical Processing Unit, in contrast to RF Hallucination, which takes around 8.5 min.

**Extended Data Fig. 4:**
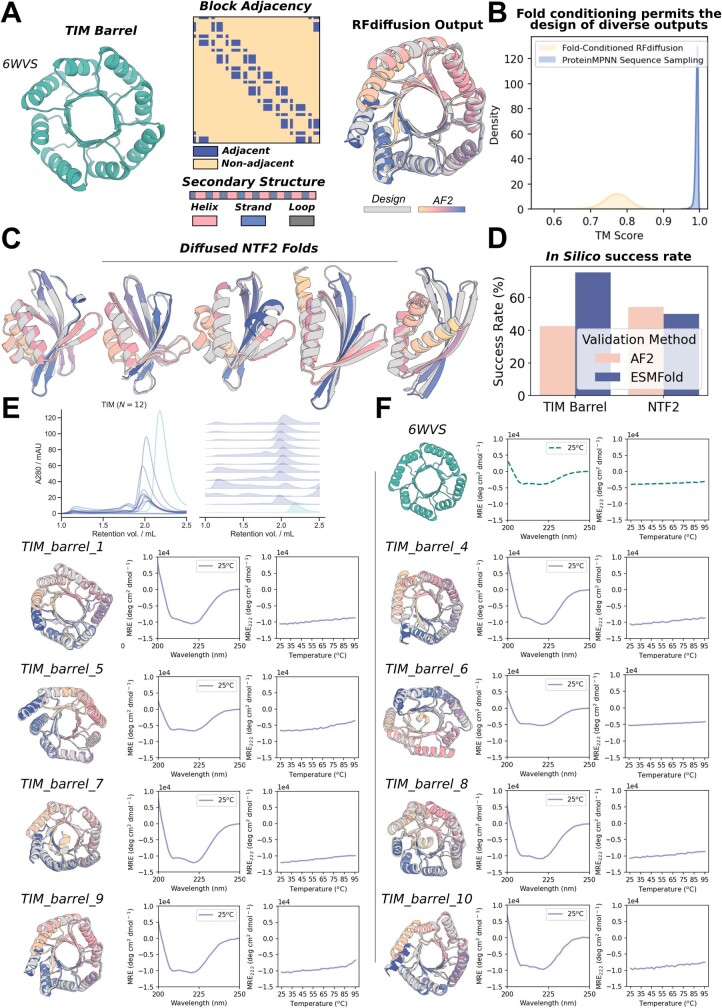
RFdiffusion can condition on fold information to generate specific, thermostable folds. **A**) 6WVS is a previously-described de novo designed TIM barrel (left). A fine-tuned RFdiffusion model can condition on 1D and 2D inputs representing this protein fold, specifically secondary structure (middle, bottom) and block-adjacency information (middle, top) (see Supplementary Methods 4.3.2). RFdiffusion then generates proteins that closely recapitulate this course-grained fold information (right). **B**) Outputs are diverse with respect to each other. With this coarse-grained fold specification, in silico successful designs are much more diverse (as quantified by pairwise TM-scores) compared to diversity generated through simply sampling many sequences for the original PDB backbone (6WVS). **C**) NTF2 folds are useful scaffolds for de novo enzyme design^56^, and can also be readily generated with fold-conditioning in RFdiffusion. Designs are diverse and closely recapitulated by AF2. **D**) In silico success rates are high with fold-conditioned diffusion. TIM barrels are generated with an AF2 in silico success rate of 42.5% (left bar, pink) with in silico success incorporating both AF2 metrics and a TM-score vs 6WVS > 0.5. NTF2 folds are generated with an AF2 in silico success rate of 54.1% (right bar, pink), with in silico success incorporating both AF2 metrics and a TM-score vs PDB: 1GY6 > 0.5. In silico success was further validated with ESMFold (blue bars), where a pLDDT > 80 was used as the confidence metric for success. Gray: RFdiffusion design, colors: AF2 prediction. **E**) 11 TIM barrel designs were purified alongside the 6WVS positive control. Ten of these express and elute predominantly as monomers (note that the designs are approximately 4kDa larger than 6WVS). **F**) Eight designs expressed sufficiently for analysis by circular dichroism. All designs are folded, with circular dichroism spectra consistent with the designed structure (middle), and similar to 6WVS. Designs were also all highly thermostable, with CD melt analyses demonstrating designs were folded even at 95 °C (right). Designs are shown in gray, with the AF2 predictions overlaid in colors (left). Note that data on 6WVS and *TIM_barrel_6* are duplicated from Fig. 2g, reproduced here for clarity.

It is often desirable to be able to specify a protein fold during design (such as triose-phosphate isomerase (TIM) barrels or cavity-containing NTF2s for small molecule binder and enzyme design^32,33^), and thus we further fine-tuned RFdiffusion to condition on secondary structure and/or fold information, enabling rapid and accurate generation of diverse designs with the desired topologies (Fig. 2g and Extended Data Fig. 4). In silico success rates were 42.5 and 54.1% for TIM barrels and NTF2 folds, respectively (Extended Data Fig. 4d), and experimental characterization of 11 TIM barrel designs indicated that at least eight designs were soluble, thermostable and had circular dichroism spectra consistent with the design model (Fig. 2g and Extended Data Fig. 4e,f).

## Design of higher-order oligomers

There is considerable interest in designing symmetric oligomers, which can serve as vaccine platforms^34^, delivery vehicles^35^ and catalysts^36^. Cyclic oligomers have been designed using structure prediction networks with an adaptation of Hallucination that searches for sequences predicted to fold to the desired cyclic symmetry, but this approach fails for higher-order dihedral, tetrahedral, octahedral and icosahedral symmetries, probably in part because of the much lower representation of such structures in the PDB^7^.

**Extended Data Fig. 5:**
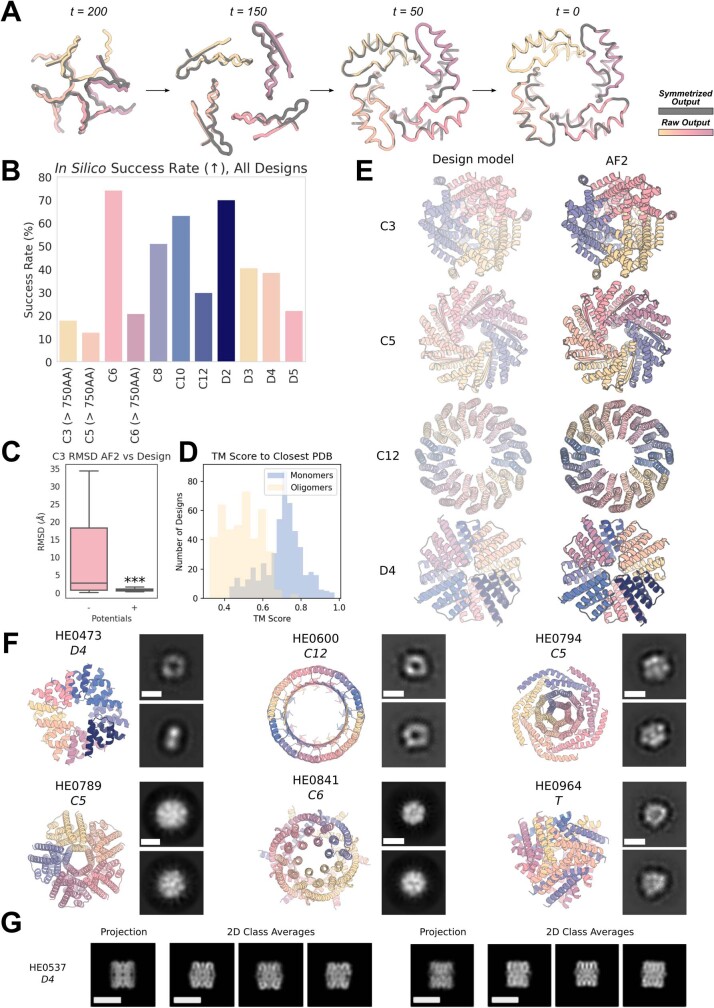
Symmetric oligomer design with RFdiffusion. **A**) Due to the (near-perfect - see Supplementary Methods 3.1) equivariance properties of RFdiffusion, X_0_ predictions from symmetric inputs are also symmetric, even at very early timepoints (and becoming increasingly symmetric through time; r.m.s.d. vs symmetrized: *t* = *200* 1.20 Å; *t* = *150* 0.40 Å; *t* = *50* 0.06 Å; *t* = *0* 0.02Å). Gray: symmetrized (top left) subunit; colors: RFdiffusion X0 prediction. **B**) In silico success rates for symmetric oligomer designs of various cyclic and dihedral symmetries. In silico success is defined here as the proportion of designs for which AF2 yields a prediction from a single sequence that has mean pLDDT > 80 and backbone r.m.s.d. over the oligomer between the design model and AF2 < 2Å. Note that 16 sequences per RFdiffusion design were sampled. **C**) Box plots of the distribution of backbone r.m.s.d.s between AF2 and the RFdiffusion design model with and without the use of external potentials during the trajectory. The external potentials used are the ‘inter-chain’ contact potential (pushing chains together), as well as the ‘intra-chain’ contact potential (making chains more globular). Using these potentials dramatically improves in silico success (Two-proportion z-test of in silico success rate: *n* = 100 designs per condition, *z* = *4.3, p* = *1.9e-5)*. **D**) Designs are diverse with respect to the training dataset (the PDB). While the monomers (typically 60–100 AA) show reasonable alignment to the PDB (median 0.72), the whole oligomeric assemblies showed little resemblance to the PDB (median 0.50). **E**) Additional examples of design models (left) against AF2 predictions (right) for C3, C5, C12, and D4 symmetric designs (the symmetries not displayed in Fig. 3) with backbone r.m.s.d.s (Å) against their AF2 predictions of 0.82, 0.63, 0.79, and 0.78 with total amino acids 750, 900, 960, 640. **F**) Additional nsEM data for symmetric designs. The model is shown on the left and the 2D class averages on the right for each design. **G**) Two orthogonal side views of *HE0537* by cryo-EM. Representative 2D class averages from the cryo-EM data are shown to the right of 2D projection images of the computational design model (lowpass filtered to 8 Å), which appear nearly identical to the experimental data. Scale bars shown (white) are 60 Å. Boxplot represents median ± IQR; tails: min/max excluding outliers (±1.5xIQR).

We set out to generalize RFdiffusion to create symmetric oligomeric structures with any specified point group symmetry. Given a specification of a point group symmetry for an oligomer with *n* chains, and the monomer chain length, we generate random starting residue frames for a single monomer subunit as in the unconditional generation case, and then generate *n* − 1 copies of this starting point arranged with the specified point group symmetry. Because RFdiffusion is equivariant (inherited from RF) with respect to rotation and relabelings of chains, symmetry is largely maintained in the denoising predictions; we explicitly resymmetrize at each step but this changes the structures only slightly (compare grey and coloured chains in Extended Data Fig. 5a and Supplementary Methods). For octahedral and icosahedral architectures, we explicitly model only the smallest subset of monomers required to generate the full assembly (for example, for icosahedra, the subunits at the five-, three- and twofold symmetry axes) to reduce the computational cost and memory footprint.

**Fig. 3:**
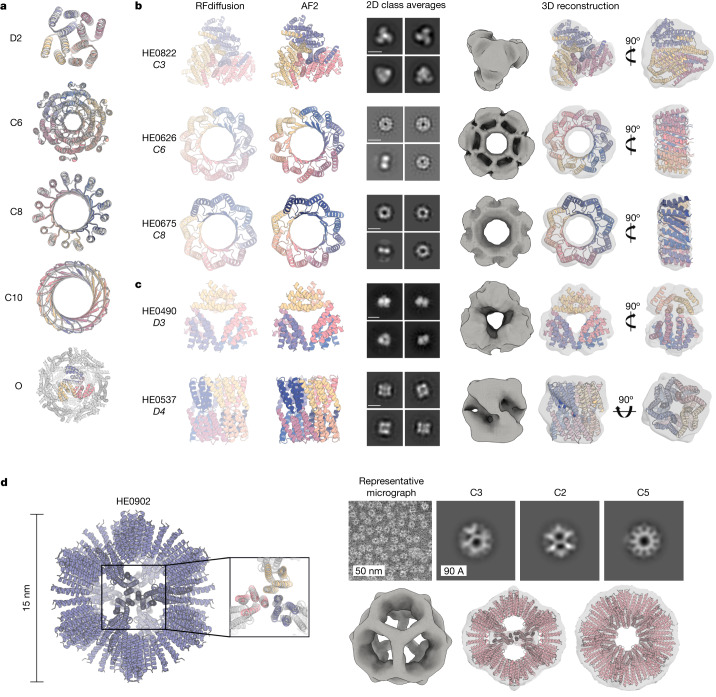
Design and experimental characterization of symmetric oligomers. **a**, RFdiffusion-generated assemblies overlaid with the AF2 structure predictions based on the designed sequences; in all five cases they are nearly indistinguishable (for the octahedron (bottom), the prediction was for the C3 substructure). Symmetries are indicated to the left of the design models. **b**,**c**, Designed assemblies characterized by nsEM. Model symmetries are as follows: cyclic, C3 (HE0822, 350 amino acids (AA) per chain), C6 (HE0626, 100 AA per chain) and C8 (HE0675, 60 AA per chain) (**b**); dihedral, D3 (HE0490, 80 AA per chain) and D4 (HE0537, 100 AA per chain) (**c**). From left to right: (1) symmetric design model, (2) AF2 prediction of design following sequence design with ProteinMPNN, (3) 2D class averages showing both top and side views (scale bar, 60 Å for all class averages) and (4) 3D reconstructions from class averages with the design model fit into the density map. The overall shapes are consistent with the design models, and confirm the intended oligomeric state. As in **a**, AF2 predictions of each design are nearly indistinguishable from the design model (backbone r.m.s.d.s (Å) for HE0822, HE0626, HE0490, HE0675 and HE0537, are 1.33, 1.03, 0.60, 0.74 and 0.75, respectively). **d**, nsEM characterization of an icosahedral particle (HE0902, 100 AA per chain). The design model, including the AF2 prediction of the C3 subunit are shown on the left. nsEM data are shown on the right: on top, a representative micrograph is shown alongside 2D class averages along each symmetry axis (C3, C2 and C5, from left to right) with the corresponding 3D reconstruction map views shown directly below overlaid on the design model.

Despite not being trained on symmetric inputs, RFdiffusion is able to generate symmetric oligomers with high in silico success rates (Extended Data Fig. 5b), particularly when guided by an auxiliary inter- and intrachain contact potential (Extended Data Fig. 5c). As illustrated in Fig. 3 and Extended Data Fig. 5e, RFdiffusion designs are nearly indistinguishable from AF2 predictions of the structures adopted by the designed sequences, and many show little resemblance to previously solved protein structures (Extended Data Fig. 5d and Supplementary Table 1). Several of the oligomeric topologies are not seen in the PDB, including two-layer beta barrels (Fig. 3a, C10 symmetry) and complex mixed alpha/beta topologies (Fig. 3a, C8 symmetry; closest TM align in PDB 6BRP, 0.47, and PDB 6BRO, 0.43, respectively).

We selected 608 designs for experimental characterization and found using size-exclusion chromatography (SEC) that at least 87 had oligomerization states closely consistent with the design models (within the 95% confidence interval, 126 designs within the 99% confidence interval, as determined by SEC calibration curves; Supplementary Figs. 4 and 5). We took advantage of the increased size of these oligomers (compared to the smaller unconditional and fold-conditioned monomers described above) and collected negative stain electron microscopy (nsEM) data on a subset of these designs across different symmetry groups. For most, distinct particles were evident with shapes resembling the design models in both the raw micrographs and subsequent two-dimensional (2D) classifications (Fig. 3 and Extended Data Fig. 5f). nsEM characterization of a C3 design (HE0822) with 350 residue subunits (1,050 residues in total) suggests that the actual structure is very close to the design, both over the 350 residue subunits and the overall C3 architecture. 2D class averages are clearly consistent with both top and side views of the design model, and a 3D reconstruction of the density has key features consistent with the design, including the distinctive pinwheel shape (Fig. 3b, top row). Electron microscopy 2D class averages of C5 and C6 designs with more than 750 residues (HE0794, HE0789, HE0841) were also consistent with the respective design models (Extended Data Fig. 5f).

RFdiffusion also generated cyclic oligomers with alpha and/or beta barrel structures that resemble expanded TIM barrels and provide an interesting comparison between innovation during natural evolution and innovation through deep learning. The TIM barrel fold, with eight strands and eight helices, is one of the most abundant folds in nature^37^. nsEM confirmed the structure of two RFdiffusion designed cyclic oligomers, which considerably extend beyond this fold (Fig. 3b, bottom rows). HE0626 is a C6 alpha–beta barrel composed of 18 strands and 18 helices, and HE0675 is a C8 octamer composed of an inner ring of 16 strands and an outer ring of 16 helices arranged locally in a very similar repeating pattern to the TIM barrel (1:1 helix:strand). For both HE0626 and HE0675 we obtained nsEM 3D reconstructions that are in agreement with the computational design models. The HE0600 design is also an alpha–beta barrel (Extended Data Fig. 5f), but has two strands for every helix (24 strands and 12 helices in total) and hence is locally different from a TIM barrel. Whereas natural evolution has extensively explored structural variations of the classic eight-strand or eight-helix TIM barrel fold, RFdiffusion can more readily explore global changes in barrel curvature, enabling discovery of TIM barrel-like structures with many more helices and strands.

RFdiffusion also readily generated structures with dihedral, tetrahedral and icosohedral symmetries (Fig. 3c,d and Extended Data Fig. 5e,f). SEC characterization indicated that 38 D2, seven D3 and three D4 designs had the expected molecular weights (these have four, six and eight chains, respectively) (Supplementary Fig. 5). Although the D2 dihedrals are too small for nsEM, 2D class averages—and for some, 3D reconstructions of D3 and D4 designs—were congruent with the overall topologies of the design models (Fig. 3c and Extended Data Fig. 5f). Similarly, 3D reconstruction (Fig. 3c) and cryogenic electron microscopy (cryo-EM) 2D class averages (Extended Data Fig. 5g and Supplementary Fig. 6) of the D4 HE0537 closely match the design model, recapitulating the roughly 45° offset between tetramic subunits. 2D nsEM class averages for a 12-chain tetrahedron (HE0964) were consistent with the design model (Extended Data Fig. 5f). Forty-eight icosahedra were selected for experimental validation, and one, HE0902, a 15 nm (diameter) highly porous assembly (Fig. 3d, left) was observed in nsEM micrographs to form homogeneous particles. 2D class averages and a 3D reconstruction very closely match the design model (Fig. 3d), with triangular hubs arrayed around the empty C5 axes. Designs such as HE0902 (and future similar large assemblies) should be useful as new nanomaterials and vaccine scaffolds, with robust assembly and (in the case of HE0902) the outward facing N and C termini offering many possibilities for antigen display.

## Functional-motif scaffolding

We next investigated the use of RFdiffusion for scaffolding protein structural motifs that carry out binding and catalytic functions, in which the role of the scaffold is to hold the motif in precisely the 3D geometry needed for optimal function. In RFdiffusion, we input motifs as 3D coordinates (including sequence and sidechains) both during conditional training and inference, and build scaffolds that hold the motif atomic coordinates in place. Many deep-learning methods have been developed recently to address this problem, including RF_joint_ Inpainting^4^, constrained Hallucination^4^ and other DDPMs^5,8,29^. To rigorously evaluate the performance of these methods in comparison to RFdiffusion across a broad set of design challenges, we established an in silico benchmark test (Supplementary Table 9) comprising 25 motif-scaffolding design problems addressed in six recent publications encompassing several design methodologies^4,5,29,38–40^. The challenges span a broad range of motifs, including simple ‘inpainting’ problems, viral epitopes, receptor traps, small molecule binding sites, binding interfaces and enzyme active sites.

**Fig. 4:**
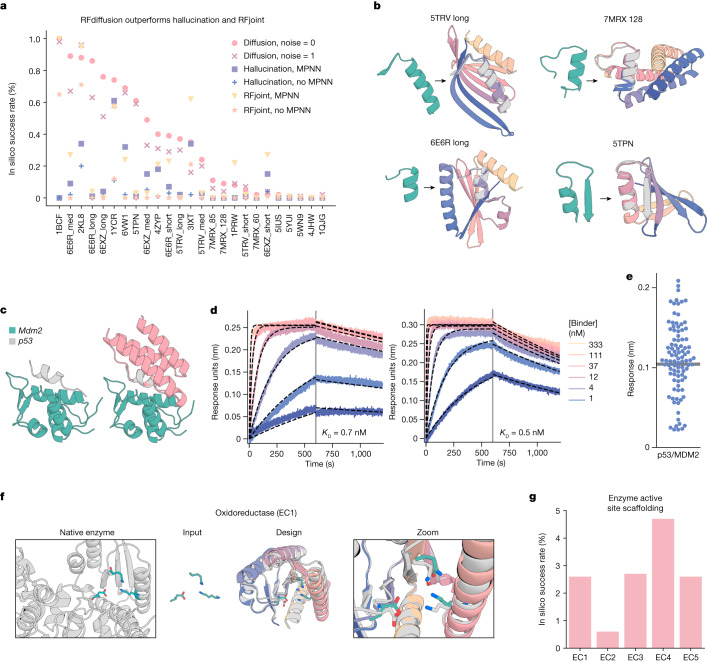
Scaffolding of diverse functional sites with RFdiffusion. **a**, RFdiffusion outperforms other methods across 25 benchmark motif-scaffolding problems collected from six recent publications (Supplementary Table 9). In silico success is defined as AF2 r.m.s.d. to design model less than 2 Å, AF2 r.m.s.d. to the native functional motif less than 1 Å and AF2 pAE less than five. One hundred designs were generated per problem, with no previous optimization on the benchmark set (some optimization was necessary for Hallucination). Supplementary Table 10 presents full results. In silico success rates on the problems are correlated between the methods, and RFdiffusion can still struggle on challenging problems in which all methods have low success. **b**, Four examples of designs in which RFdiffusion significantly outperforms existing methods. Teal, native motif; colours, AF2 prediction of a design. Metrics (r.m.s.d. AF2 versus design/versus native motif (Å), AF2 pAE): 5TRV long, 1.17/0.57; 4.73; 6E6R long, 0.89/0.27, 4.56; 7MRX long, 0.84/0.82 4.32; 5TPN, 0.59/0.49 3.77. **c**, RFdiffusion can scaffold the p53 helix that binds MDM2 (left) and makes extra contacts with the target (right, average 31% increased surface area. Design was p53_design_89). Designs were generated with an RFdiffusion model fine-tuned on complexes. **d**, BLI measurements indicate high-affinity binding to MDM2 (p53_design_89, 0.7 nM; p53_design_53, 0.5 nM*)*; the native affinity is 600 nM (ref. ^42^). **e**, Out of 95 designs, 55 showed binding to MDM2 (more than 50% of maximum response). Thirty-two of these were monomeric (Supplementary Fig. 10h). **f**, After fine-tuning (Supplementary Methods), RFdiffusion can scaffold enzyme active sites. An oxidoreductase example (EC1) is shown (PDB 1A4I); catalytic site (teal); RFdiffusion output (grey, model; colours, AF2 prediction); zoom of active site. AF2 versus design backbone r.m.s.d. 0.88 Å, AF2 versus design motif backbone r.m.s.d. 0.53 Å, AF2 versus design motif full-atom r.m.s.d. 1.05 Å, AF2 pAE 4.47. **g**, In silico success rates on active sites derived from EC1-5 (AF2 Motif r.m.s.d. versus native: backbone less than 1 Å, backbone and sidechain atoms less than 1.5 Å, r.m.s.d. AF2 versus design less than 2 Å, AF2 pAE less than 5).

RFdiffusion solves 23 of the 25 benchmark problems, compared to 15 for Hallucination and 19 for RF_joint_ Inpainting (Fig. 4a,b). For 19 out of 23 of the problems solved by RFdiffusion, the fraction of successful designs is higher than either Hallucination or RF_joint_ Inpainting. The excellent performance of RFdiffusion required no hyperparameter tuning or external potentials; this contrasts with Hallucination, for which problem-specific optimization can be required. In 17 out of 23 of the problems, RFdiffusion-generated successful solutions with higher in silico success rates when noise was not added during the reverse diffusion trajectories (see Extended Data Fig. 1i for further discussion on the effect of noise on design quality, and Supplementary Fig. 8 for analysis of design diversity). The ability of RFdiffusion to scaffold functional motifs is not related to their presence in the RFdiffusion training set (Supplementary Fig. 7).

One of the benchmark problems is the scaffolding of the p53 helix that binds MDM2. Inhibiting this interaction through high-affinity competitive inhibition by scaffolding the p53 helix and making further interactions with MDM2 is a promising therapeutic avenue^41^. In silico success has been described elsewhere^4^, but experimental success has not been reported. We used an RFdiffusion model fine-tuned on protein complexes (Supplementary Methods) to generate 96 designs scaffolding this helix. We scaffolded the p53 helix in the presence of MDM2, so extra interactions could be designed by RFdiffusion and experimentally identified 0.5 and 0.7 nM binders (Fig. 4c,d), three orders of magnitude higher affinity than the reported 600 nM affinity of the p53 peptide alone^42^. The overall success rate was quite high: out of the 96 designs, 55 showed some detectable binding at 10 μM (Fig. 4e and Supplementary Fig. 10h).

## Scaffolding enzyme active sites

**Extended Data Fig. 6:**
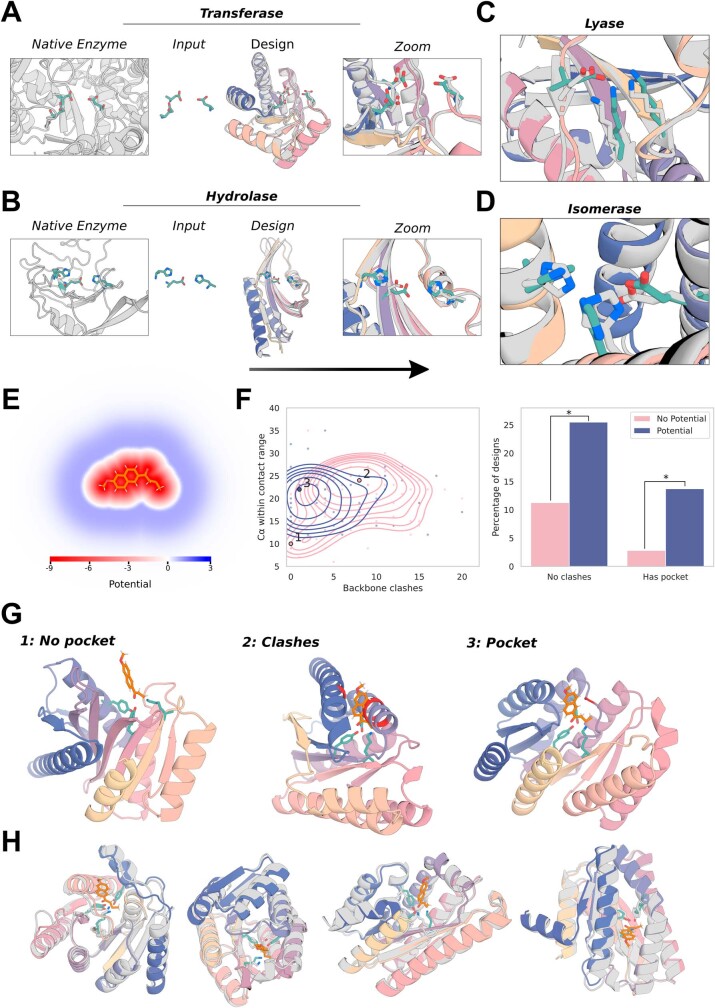
External potentials for generating pockets around substrate molecules. **A**–**D**) Example in silico successful designs for enzyme classes 2–5 (ref. ^57^, see also Fig. 4). Native enzyme (PDB: 1CWY, 1DE3, 1P1X, 1SNZ); catalytic site (teal); RFdiffusion output (gray: model, colors: AF2 prediction). Metrics (AF2 vs design backbone r.m.s.d., AF2 vs design motif backbone r.m.s.d., AF2 vs design motif full-atom r.m.s.d., AF2 pAE): EC2: 0.93 Å, 0.50 Å, 1.29 Å, 3.51; EC3: 0.92 Å, 0.60 Å, 1.07 Å, 4.59; EC4: 0.93 Å, 0.80 Å, 1.03 Å, 4.41; EC5: 0.78 Å, 0.44 Å, 1.14 Å, 3.32. **E**–**H**) Implicit modeling of a substrate while scaffolding a retroaldolase active site triad [TYR1051-LYS1083-TYR1180] from PDB: 5AN7. **E**) The potential used to implicitly model the substrate, which has both a repulsive and attractive field (see Supplementary Methods 4.4). **F**) Left: Kernel densities demonstrate that without using the external potential (pink), designs often fall into two failure modes: (1) no pocket, and (2) clashes with the substrate. Right: clashes (substrate < 3 Å of the backbone) & pockets (no clash and > 16 Cα within 3–8 Å of substrate) with and without the potential. Two-proportion z-test: *n* = 71/51 +/− potential; clashes *z* = *−2.05, p* = *0.02*, pocket *z* = *−2.27, p* = *0.01*. Each datapoint represents a design already passing the stringent in silico success metrics (AF2 motif r.m.s.d. < 1 Å, AF2 backbone r.m.s.d. < 2 Å, AF2 pAE < 5). Note that the potential and clash definition pertain only to backbone Cα atoms, and do not currently include sidechain atoms. **G**) Designs close to the labeled local maxima of the kernel density estimate. Without the potential, the catalytic triad is predominantly (*1*) exposed on the surface with no residues available to provide substrate stabilization or (*2*) buried in the protein core, preventing substrate access. With the potential, the catalytic triad is predominantly (*3*), partially buried in a concave pocket with shape complementary to the substrate. Backbone atoms within 3 Å of the substrate are shown in red. **H**) A variety of diverse designs with pockets made using the potential, with no clashes between the substrate and the AF2-predicted backbone. The functional form and parameters used for the pocket potential are detailed in Supplementary Methods 4.4. In each case the substrate is superimposed on the AF2 prediction of the catalytic triad.

A grand challenge in protein design is to scaffold minimal descriptions of enzyme active sites comprising a few single amino acids. Whereas some in silico success has been reported previously^4^, a general solution that can readily produce high-quality, orthogonally validated outputs remains elusive. Following fine-tuning on a task mimicking this problem (Supplementary Methods), RFdiffusion was able to scaffold enzyme active sites comprising many sidechain and backbone functional groups with high accuracy and in silico success rates across a range of enzyme classes (Fig. 4f and Extended Data Fig. 6a–d; in silico success required fine tuning). Although RFdiffusion is unable to explicitly model bound small molecules at present (however, see our conclusions), the substrate can be implicitly modelled using an external potential to guide the generation of ‘pockets’ around the active site. As a demonstration, we scaffold a retroaldolase active site triad while implicitly modelling the reaction substrate (Extended Data Fig. 6e–h).

## Symmetric functional-motif scaffolding

**Fig. 5:**
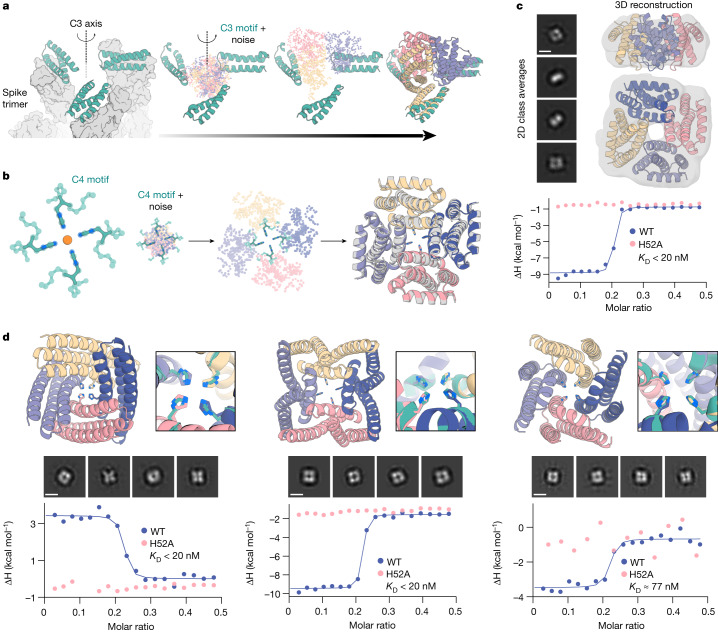
Symmetric motif scaffolding with RFdiffusion. **a**, Design of symmetric oligomers scaffolding the binding interface of ACE2 mimic AHB2 (left, teal) against the SARS-CoV-2 spike trimer (left, grey). Three AHB2 copies are input to RFdiffusion along with C3 noise (middle); output are C3-symmetric oligomers holding the three AHB2 copies in place to engage all spike subunits. AF2 predictions (right) recapitulate the AHB2 structure with 0.6 Å r.m.s.d. over the assymetric unit and 2.9 Å r.m.s.d. over the C3 assembly. **b**, Design of C4-symmetric oligomers to scaffold a Ni^2+^ binding motif (left). Starting from square-planar histidine rotamers within helical fragments (Supplementary Methods), RFdiffusion generates a C4 oligomer scaffolding the binding domain (middle). AF2 predictions (colour) agree closely with the design model (grey), with backbone r.m.s.d. less than 1.0 Å (right). **c**, nsEM 2D class averages (scale bar, 60 Å) and 3D reconstruction density are consistent with the symmetry and structure of the NiB1.17 design model shown superimposed on the density in ribbon representation (top). Isothermal titration calorimetry binding isotherm of design NiB1.17 (blue) indicates a dissociation constant less than 20 nM at a metal:monomer stoichiometry of 1:4. The H52A mutant isotherm (pink) ablates binding, indicating scaffolded histidine residues are critical for metal binding. **d**, Additional experimentally characterized Ni^2+^ binders NiB2.15 (left), NiB1.12 (middle) and NiB1.20 (right). Metal-coordinating sidechains in the design models (top, teal) are closely recapitulated in the AF2 predictions (colours). 2D nsEM class averages (middle; scale bar, 60 Å) are consistent with design models. Binding isotherms for wild-type (WT) and H52A mutant (bottom) indicate Ni^2+^ binding mediated directly by the scaffolded histidines at the designed stoichiometry. Note that for ITC plots, points represent single measurements.

Several important design challenges involve the scaffolding of several copies of a functional motif in symmetric arrangements. For example, many viral glycoproteins are trimeric and symmetry matched arrangements of inhibitory domains can be extremely potent^43–46^. Conversely, symmetric presentation of viral epitopes in an arrangement that mimics the virus could induce new classes of neutralizing antibodies^47,48^. To explore this general direction, we sought to design trimeric multivalent binders to the SARS-CoV-2 spike protein. In previous work, flexible linkage of a binder to the ACE2 binding site (on the spike protein receptor binding domain) to a trimerization domain yielded a high-affinity inhibitor that had potent and broadly neutralizing antiviral activity in animal models^43^. Ideally, however, symmetric fusions to binders would be rigid, so as to reduce the entropic cost of binding while maintaining the avidity benefits from multivalency. We used RFdiffusion to design C3-symmetric trimers that rigidly hold three binding domains (the functional motif in this case) such that they exactly match the ACE2 binding sites on the SARS-CoV-2 spike protein trimer. The designs were confidently predicted by AF2 to both assemble as C3-symmetric oligomers, and to scaffold the AHB2 SARS-CoV-2 binder interface with high accuracy (Fig. 5a).

The ability to scaffold functional sites with any desired symmetry opens up new approaches to designing metal-coordinating protein assemblies^49,50^. Divalent transition metal ions show distinct preferences for specific coordination geometries (for example, square planar, tetrahedral and octahedral) with ion-specific optimal sidechain–metal bond lengths. RFdiffusion provides a general route to building up symmetric protein assemblies around such sites, with the symmetry of the assembly matching the symmetry of the coordination geometry. As a first test, we sought to design square-planar Ni^2+^ binding sites. We designed C4 protein assemblies with four central histidine imidazoles arranged in an ideal Ni^2+^-binding site with square-planar coordination geometry (Fig. 5b). Diverse designs starting from distinct C4-symmetric histidine square-planar sites had good in silico success with the histidine residues in near ideal geometries for coordinating metal in the AF2-predicted structures (Supplementary Fig. 9).

**Extended Data Fig. 7:**
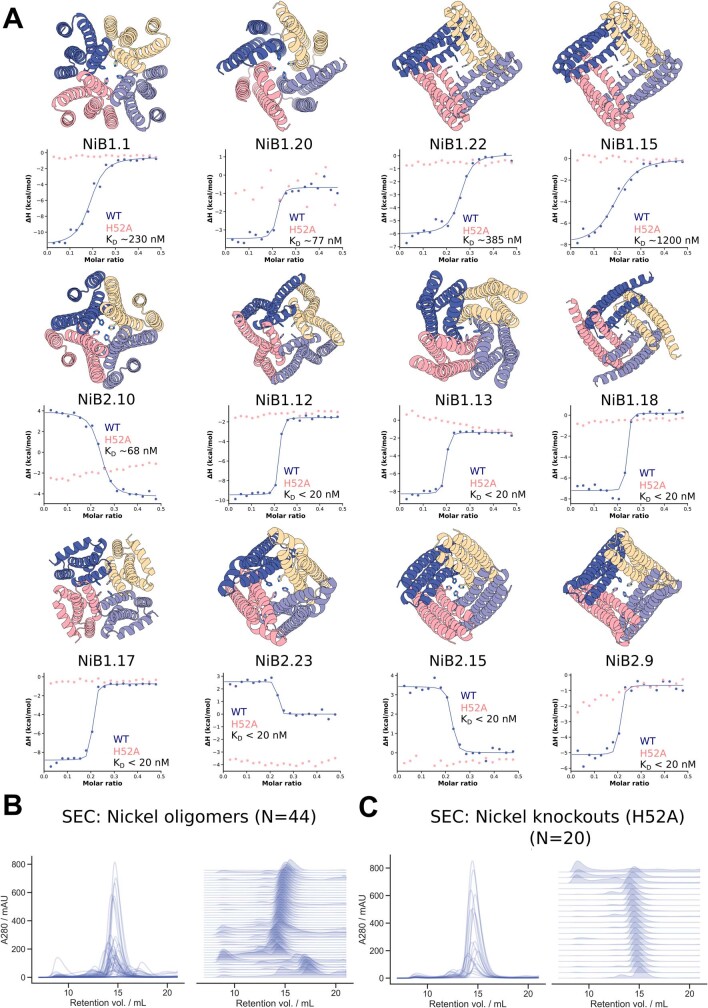
Additional Ni^2+^ binding C4 oligomers. **A**) AF2 predictions of a subset of the experimentally verified Ni^2+^ binding oligomers, with corresponding isothermal titration calorimetry (ITC) binding isotherms for the wild-type (blue) and H52A mutant (pink) below. Note that these, with Fig. 5, encompass all of the experimentally validated outputs deriving from unique RFdiffusion backbones. Wild-type dissociation constants are displayed in each plot. We observe a mixture of endothermic (*NiB2.10*, *NiB2.23*, *NiB2.15*) and exothermic isotherms. For all cases displayed we observe no binding to the ion for H52A mutants, indicating the scaffolded histidine at position 52 is critical for ion binding. K_D_ values in the isotherms indicate binding of the ion with the designed stoichiometry (1:4 Ni^2+^:protein). Note that each backbone depicted is from a unique RFdiffusion sampling trajectory, and that models and data for designs *NiB2.15*, *NiB1.12*, *NiB1.20* and *NiB1.17* from Fig. 5 are duplicated here for ease of viewing. **B**) Size exclusion chromatograms for elutions from the 44 purifications suggest the vast majority of designs are soluble and have the correct oligomeric state. **C**) Size exclusion chromatograms for 20 H52A mutants show that the mutants remain soluble and retain the intended oligomeric state. Note that only 18 of these 20 had wild-type sequences that definitively bound nickel. Note also that for ITC plots, points represent single measurements.

We expressed and purified 44 designs in *Escherichia coli*, and found that 37 had SEC chromatograms consistent with the intended oligomeric state (Extended Data Fig. 7b). Of the designs, 36 were tested for Ni^2+^ coordination by isothermal titration calorimetry, and 18 were found to bind Ni^2+^ with dissociation constants ranging from low nanomolar to low micromolar (Fig. 5c,d and Extended Data Fig. 7a). The inflection points in the wild-type isotherms indicate binding with the designed stoichiometry, a one to four ratio of ion to monomer. Although most of the designed proteins showed exothermic metal coordination, in a few cases binding was endothermic (Fig. 5d, left and Extended Data Fig. 7a: NiB2.9, NiB2.10, NiB2.15 and NiB2.23), suggesting that Ni^2+^ coordination is entropically driven in these assemblies. To confirm that Ni^2+^ binding was indeed mediated by the scaffolded histidine 52, we mutated this residue to alanine, which abolished or notably reduced binding in 17 out of 17 cases with successful expression (Extended Data Figs. 7a,c and Fig. 5c,d; one mutant did not express). We structurally characterized by nsEM a subset of the designs—NiB1.12, NiB1.15, NiB1.17 and NiB1.20—that showed histidine-dependent binding. All four designs showed clear fourfold symmetry both in the raw micrographs and in 2D class averages (Fig. 5c,d), with design NiB1.17 also clearly showing twofold axis side views with a measured diameter approximating the design model. A 3D reconstruction of NiB1.17 was in close agreement with the design model (Fig. 5c).

## Design of protein-binding proteins

The design of high-affinity binders to target proteins is a grand challenge in protein design, with numerous therapeutic applications^51^. A general method for de novo binder design from target structure information alone using the physically based Rosetta method was recently described^12^, and subsequently, using ProteinMPNN for sequence design and AF2 for design filtering was found to improve design success rates^26^. However, experimental success rates were low, still requiring many thousands of designs to be screened for each design campaign^12^, and the approach relied on prespecifying a particular set of protein scaffolds as the basis for the designs, inherently limiting the diversity and shape complementarity of possible solutions^12^. To our knowledge, no deep-learning method has yet demonstrated experimental general success in designing completely de novo binders.

**Fig. 6:**
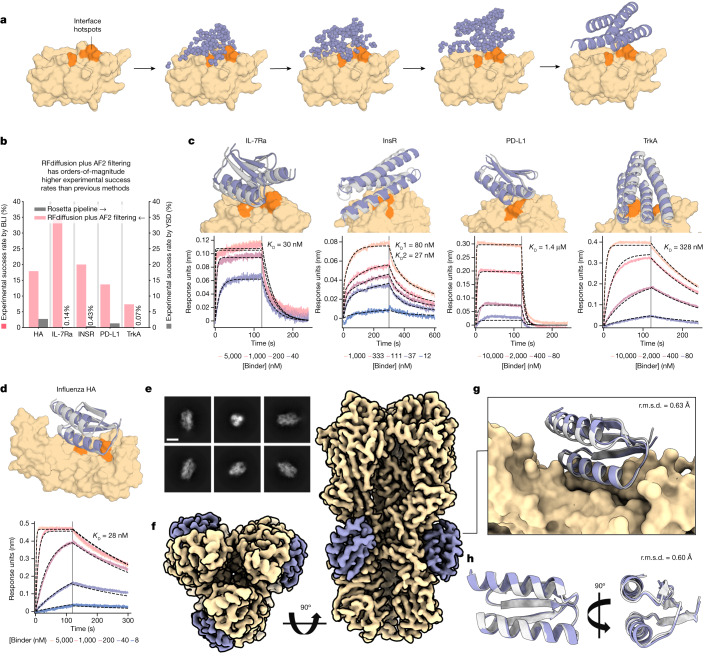
De novo design of protein-binding proteins. **a**, RFdiffusion generates protein binders given a target and specification of interface hotspot residues. **b**, De novo binders were designed to five protein targets; Influenza A H1 HA, IL-7Rα, InsR, PD-L1 and TrkA and hits with BLI response greater than or equal to 50% of the positive control were identified for all targets. For IL-7Rα, InsR, PD-L1 and TrkA, RFdiffusion has success rates roughly two orders of magnitude higher than the original design campaigns. We attribute one order of magnitude to RFdiffusion, and the second to filtering with AF2 (estimated success rates for previous campaigns if AF2 filtering had been used: HA, 0%; IL-7Rα, 2.2%; InsR, 5.5%; PD-L1, 3.7%; TrkA, 1.5%). **c**, For IL-7Rα, InsR, PD-L1 and TrkA, the highest affinity binder is shown above a BLI titration series. Reported *K*_D_ values are based on global kinetic fitting with fixed global *R*_max_. **d**, The highest affinity HA binder, HA_20, binds with a *K*_D_ of 28 nM. **c**,**d**, Yellow or orange, target or hotspot residues; grey, design model; purple, AF2 prediction (r.m.s.d. AF2 versus design). Binders: IL7Ra_55 (2.1 Å), InsulinR_30 (2.6 Å), PDL1_77 (1.5 Å), TrkA_88 (1.4 Å) (left to right in **c**) and HA_20 (1.7 Å) (**d**). **e**, Cryo-EM 2D class averages of *HA_20* bound to influenza HA, strain A/USA:Iowa/1943 H1N1 (scale bar, 10 nm). **f**, 2.9 Å cryo-EM 3D reconstruction of the complex viewed along two orthogonal axes. *HA_20* (purple) is bound to H1 along the stem of all three subunits. **g**, The cryo-EM structure of the HA_20 binder in complex closely matches the design model (r.m.s.d. to RFdiffusion design, 0.63 Å; yellow, influenza HA). **h**, Structure of the HA_20 binder alone superimposed on the design model viewed along two orthogonal axes. For cryo-EM panels, yellow, Influenza H1 map and/or structure; grey, HA_20 binder design model; purple, HA_20 binder map or structure.

**Extended Data Fig. 8:**
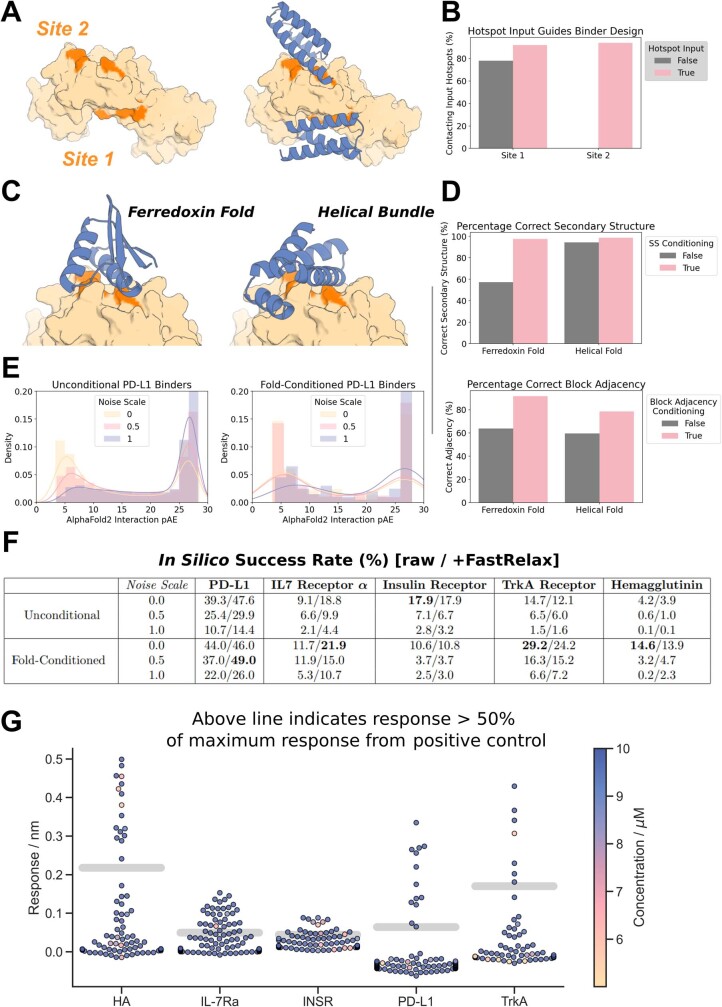
Targeted unconditional and fold-conditioned protein binder design. **A**-**B**) The ability to specify where on a target a designed binder should bind is crucial. Specific “hotspot” residues can be input to a fine-tuned RFdiffusion model, and with these inputs, binders almost universally target the correct site. **A**) IL-7Rα (PDB: 3DI3) has two patches that are optimal for binding, denoted Site 1 and Site 2 here. For each site, 100 designs were generated (without fold-specification). **B**) Without guidance, designs typically target Site 1 (left bar, gray), with contact defined as Cα-Cα distance between binder and hotspot reside < 10 Å. Specifying Site 1 hotspot residues increases further the efficiency with which Site 1 is targeted (left bar, pink). In contrast, specifying the Site 2 hotspot residues can completely redirect RFdiffusion, allowing it to efficiently target this site (right bar, pink). **C**-**D**) As well as conditioning on hotspot residue information, a fine-tuned RFdiffusion model can also condition on input fold information (secondary structure and block-adjacency information - see Supplementary Methods 4.5). This effectively allows the specification of a (for instance, particularly compatible) fold that the binder should adopt. **C**) Two examples showing binders can be specified to adopt either a ferredoxin fold (left) or a particular helical bundle fold (right). **D**) Quantification of the efficiency of fold-conditioning. Secondary structure inputs were accurately respected (top, pink). Note that in this design target and target site, RFdiffusion without fold-specification made generally helical designs (right, gray bar). Block-adjacency inputs were also respected for both input folds (bottom, pink). **E**) Reducing the noise added at each step of inference improves the quality of binders designed with RFdiffusion, both with and without fold-conditioning. As an example, the distribution of AF2 interaction pAEs (known to indicate binding when pAE < 10^26^) is shown for binders designed to PD-L1. In both cases, the proportion of designs with interaction pAE < 10 is high (blue curve), and improved when the noise is scaled by a factor 0.5 (pink curve) or 0 (yellow curve). **F**) Full *in silico* success rates for the protein binders designed to five targets. In each case, the best fold-conditioned results are shown (i.e. from the most target-compatible input fold), and the success rates at each noise scale are separated. In line with current best practice^26^, we tested using Rosetta FastRelax^58^ before designing the sequence with ProteinMPNN, but found that this did not systematically improve designs. *In silico* success is defined in line with current best practice^26^: AF2 pLDDT of the monomer > 80, AF2 interaction pAE < 10, AF2 r.m.s.d. monomer vs design < 1 Å. **G**) Experimentally-validated de novo protein binders were identified for all five of the targets. Designs that bound at 10 μM during single point BLI screening with a response equal to or greater than 50% of the positive control were considered binders. Concentration is denoted by hue for designs that were screened at concentrations less than 10 μM and thus may be false negatives.

We reasoned that RFdiffusion might be able to address this challenge by directly generating binding proteins in the context of the target. For many therapeutic applications, for example, blocking a protein–protein interaction, it is desirable to bind to a particular site on a target protein. To enable this, we fine-tuned RFdiffusion on protein complex structures, providing a feature as input indicating a subset of the residues on the target chain (called ‘interface hotspots’) to which the diffused chain binds (Fig. 6a and Extended Data Fig. 8a,b). For design challenges in which a particular binder fold might be especially compatible, we enabled coarse-grained control over binder scaffold topology by fine-tuning an extra model to condition binder diffusion on secondary structure and block-adjacency information, in addition to conditioning on interface hotspots (Extended Data Fig. 8c,d and Supplementary Methods).

To compare RFdiffusion to previous binder design methods, we performed binder design campaigns against five targets: Influenza A H1 Haemagglutinin (HA)^52^, Interleukin-7 Receptor-ɑ (IL-7Rɑ)^12^, Programmed Death-Ligand 1 (PD-L1)^12^, Insulin Receptor (InsR) and Tropomyosin Receptor Kinase A (TrkA)^12^. We designed putative binders to each target, both with and without conditioning on compatible fold information, with high in silico success rates (Extended Data Fig. 8e,f). Designs were filtered by AF2 confidence in the interface and monomer structure^26^, and 95 were selected for each target for experimental characterization.

The designed binders were expressed in *E. coli* and purified, and binding was assessed through single point biolayer interferometry (BLI) screening at 10 μM binder concentration (Extended Data Fig. 8g). The overall experimental success rate, defined as binding at or above 50% of the maximal response for the positive control, was 19% (this is a conservative estimate as some designs that showed binding had insufficient material to permit screening at 10 μM: Extended Data Fig. 8g); an increase of roughly two orders of magnitude over our previous Rosetta-based method on the same targets (Fig. 6b). Binders were identified for all five targets, with fewer than 100 designs tested per target compared to thousands in previous studies. Full BLI titrations for a subset of the designs showed nanomolar affinities with no further experimental optimization, including HA and IL-7Rɑ binders with affinities of roughly 30 nM (Fig. 6c). Binding interfaces were often highly distinct from interfaces to these targets in the PDB (Supplementary Figs. 11 and 12). To assess binder specificity, six of the highest affinity IL-7Rɑ binders were assessed by means of competition BLI, and all six competed for binding with a structurally validated positive control binding to the same site (Supplementary Fig. 10a; further work is required to fully characterize proteome-wide specificity).

**Extended Data Fig. 9:**
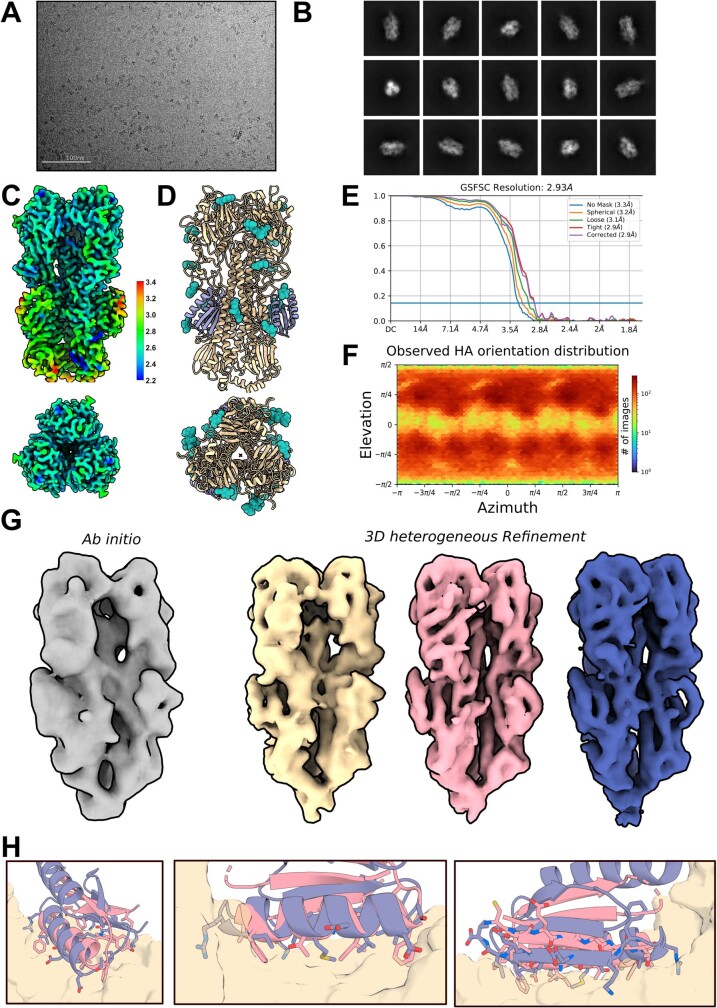
Cryo-electron microscopy structure determination of designed Influenza HA binder. **A**) Representative raw micrograph showing ideal particle distribution and contrast. **B**) 2D Class averages of Influenza H1+*HA_20* binder with clearly defined secondary structure elements and a full-sampling of particle view angles (scale bar = 10 nm). **C**) Cryo-EM local resolution map calculated using an FSC value of 0.143 viewed along two different angles. Local resolution estimates range from ~2.3 Å at the core of H1 to ~3.4 Å along the periphery of the N-terminal helix of the *HA_20* binder. **D**) Cryo-EM structure of the full H1+*HA_20* binder complex (purple: *HA_20*; yellow: H1; teal: glycans). **E**) Global resolution estimation plot. **F**) Orientational distribution plot demonstrating complete angular sampling. **G**) 3D ab initio (left) and 3D heterogenous refinement (right - unsharpened) outputs, performed in the absence of applied symmetry, and showing clear density of the *HA_20* binder bound to all three stem epitopes of the Iowa43 HA glycoprotein trimer, in all maps. **H**) The designed binder has topological similarity to 5VLI, a protein in the PDB, but binds with very different interface contacts.

We solved the structure of the highest affinity Influenza binder, *HA_20*, in complex with Iowa43 HA using cryo-EM (Extended Data Table 1). Raw electron micrographs revealed a well-folded HA glycoprotein with clearly discernible side, top and tilted view orientations suspended in a thin layer of vitreous ice (Extended Data Fig. 9a). The 2D class averages further show clear secondary structure elements corresponding to both Iowa43 HA (Extended Data Fig. 9b), as well as the *HA_20* binder bound to the stem (Fig 6e). The 3D heterogenous refinement without symmetry revealed full occupancy of all three HA stem epitopes by the *HA_20* binder. A final non-uniform 3D refinement reconstruction with C3 symmetry yielded a 2.9 Å map of the HA/*HA_20* protein–protein complex (Fig 6f) and corresponding 3D structure that almost perfectly matches the computational design model (0.63 Å, Fig 6f,g; the sidechain interactions at the interface are very different from the closest structure in the PDB; Extended Data Fig. 9h). Over the binder alone, the experimental structure deviates from the RFdiffusion design by only 0.6 Å (Fig. 6h). These results demonstrate the ability of RFdiffusion to generate new proteins with atomic level accuracy, and to precisely target functionally relevant sites on therapeutically important proteins.

## Discussion

RFdiffusion is a comprehensive improvement over current protein design methods. RFdiffusion readily generates diverse unconditional designs up to 600 residues in length that are accurately predicted by AF2, far exceeding the complexity and accuracy achieved by most previous methods (a recent Hallucination-based approach also achieved high unconditional performance^53^). Half of our tested unconditional designs express in a soluble way,  and have circular dichroism spectra consistent with the design models and high thermostability. Despite their substantially increased complexity, the ideality and stability of RFdiffusion designs is akin to that of de novo protein designs generated using previous methods such as Rosetta. RFdiffusion enables generation of higher-order architectures with any desired symmetry, unlike Hallucination methods, which have so far been limited to cyclic symmetries. Electron microscopy confirmed that the structures of these oligomers are very similar to the design models, which in many cases show little global similarity to known protein oligomers.

There has been recent progress in scaffolding protein functional motifs using deep-learning methods (RF Hallucination, RF_joint_ Inpainting and diffusion), but Hallucination is slow for large systems, Inpainting fails when insufficient starting information is provided and previous diffusion methods had low accuracy. RFdiffusion outperforms these previous methods in the complexity of the motifs that can be scaffolded, the precision with which sidechains are positioned (for catalysis and other functions), and the accuracy of motif recapitulation by AF2. The design of MDM2 binding proteins with three orders of magnitude higher affinities than the scaffolded P53 motif demonstrates the robustness of RFdiffusion motif scaffolding. Combining accurate motif scaffolding with the design of symmetric assemblies enabled consistent and atomically precise positioning of sidechains to coordinate Ni^2+^ ions across diverse tetrameric assemblies

For binder design from target structural information alone, previous work required testing tens of thousands of sequences^12^. RFdiffusion, when combined with improved filtering^26^ raises experimental success rates by two orders of magnitude; high-affinity binders can be identified from dozens of designs, in many cases eliminating the requirement for slow and expensive high-throughput screening (at least for the non-polar sites targeted here; further studies will be required to assess success rates on more polar target sites and sites without native binding partners). A high-resolution cryo-EM structure of one of these designs in complex with influenza HA shows that RFdiffusion can design functional proteins with atomic accuracy. Vázquez Torres et al. demonstrate the ability of RFdiffusion to design picomolar affinity binders to flexible helical peptides^54^, further highlighting its use for de novo binder design. Vázquez Torres et al. also show how RFdiffusion can be extended for protein model refinement by partial noising and denoising, which enables tuneable sampling around a given input structure. For peptide binder design, this enabled increases in affinity of nearly three orders of magnitude without high-throughput screening.

The breadth and complexity of problems solvable with RFdiffusion and the robustness and accuracy of the solutions far exceeds what has been achieved previously. In a manner reminiscent of the generation of images from text prompts, RFdiffusion makes possible, with minimal specialist knowledge, the generation of functional proteins from minimal molecular specifications (for example, high-affinity binders to a user-specified target protein, and diverse protein assemblies from user-specified symmetries).

The power and scope of RFdiffusion can be extended in several directions. RF has recently been extended to nucleic acids and protein–nucleic acid complexes^55^, which should enable RFdiffusion to design nucleic acid binding proteins and perhaps folded RNA structures. Extension of RF to incorporate ligands should similarly enable extension of RFdiffusion to explicitly model ligand atoms, and allow the design of protein–ligand interactions. The ability to customize RFdiffusion to specific design challenges by addition of external potentials and by fine-tuning (as illustrated here for catalytic site scaffolding, binder-targeting and fold specification), along with continued improvements to the underlying methodology, should enable de novo protein design to achieve still higher levels of complexity, to approach and, in some cases, surpass what natural evolution has achieved.

### Reporting summary

Further information on research design is available in the Nature Portfolio Reporting Summary linked to this article.

## Online content

Any methods, additional references, Nature Portfolio reporting summaries, source data, extended data, supplementary information, acknowledgements, peer review information; details of author contributions and competing interests; and statements of data and code availability are available at 10.1038/s41586-023-06415-8.

### Supplementary information

Supplementary Information The supplementary information file is a single PDF that contains text, figures and tables that aim to help the reader understand the theoretical underpinnings of RFdiffusion, its implementation and its application to the design challenges posed in the paper. Descriptions of in silico and experimental methods can be found within.

Reporting Summary

## Figures and Tables

**Extended Data Table 1:**
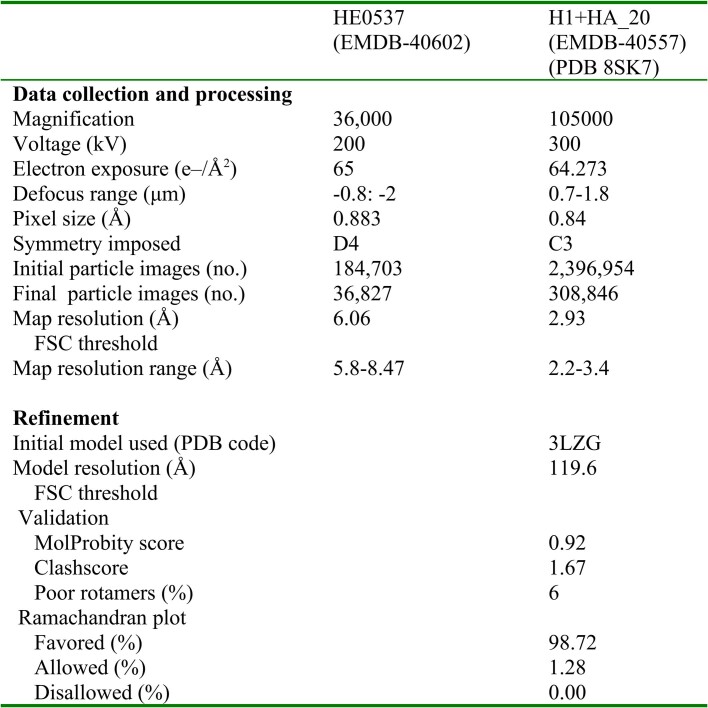
Cryo-EM data collection, refinement and validation statistics

## Data Availability

Design structures, AF2 models and experimental measurements are available at https://figshare.com/s/439fdd59488215753bc3. Cryo-EM maps and corresponding atomic models for the Influenza HA binder in Fig. 6d–h have been deposited in the PDB and the Electron Microscopy Data Bank under accession codes 8SK7 and EMDB-40557, respectively. Electron microscopy data collected for the HE0537 oligomer are available at EMDB-40602.

## Code availability

Code for running RFdiffusion has been released on GitHub, free for academic, personal and commercial use at https://github.com/RosettaCommons/RFdiffusion. It is also available as a Google Colab notebook, accessible through GitHub.
