# Supplementary material for: De novo design of protein structure and function with RFdiffusion
Source: Nature. 2023 Jul 11;620(7976):1089–100. doi: 10.1038/s41586-023-06415-8 (PMC10468394; doi:10.1038/s41586-023-06415-8)
Supplement: Supplementary file 1 — The supplementary information file is a single PDF that contains text, figures and tables that aim to help the reader understand the theoretical underpinnings of RFdiffusion, its implementation and its application to the design challenges posed in the paper. Descriptions of in silico and experimental methods can be found within. [file 41586_2023_6415_MOESM1_ESM.pdf]

nature portfolio

https://doi.org/10.1038/s41586-023-06415-8

Supplementary information

De novo design of protein structure and function with RFdiffusion

In the format provided by the authors and unedited

Supplement for De novo design of protein structure and function with RFdiffusion

Joseph L. Watson[†1,2], David Juergens[†1,2,3], Nathaniel R. Bennett[†1,2,3], Brian L. Trippe[†2,4,5], Jason Yim[†2,6], Helen E. Eisenach[†1,2], Woody Ahern[†1,2,7], Andrew J. Borst[1,2], Robert J. Ragotte[1,2], Lukas F. Milles[1,2], Basile I. M. Wicky[1,2], Nikita Hanikel[1,2], Samuel J. Pellock[1,2], Alexis Courbet[1,2,9], William Sheffler[1,2], Jue Wang[1,2], Preetham Venkatesh[1,2,8], Isaac Sappington[1,2,8], Susana Vázquez Torres[1,2,8], Anna Lauko[1,2,8], Valentin De Bortoli[9], Emile Mathieu[10], Sergey Ovchinnikov[14,15], Regina Barzilay[6], Tommi S. Jaakkola[6], Frank DiMaio[1,2], Minkyung Baek[12], and David Baker*[1,2,11]

[1]Department of Biochemistry, University of Washington, Seattle, WA 98105, USA

[2]Institute for Protein Design, University of Washington, Seattle, WA 98105, USA

[3]Graduate Program in Molecular Engineering, University of Washington, Seattle, WA 98105, USA

[4]Columbia University, Department of Statistics, New York, NY 10027, USA

[5]Irving Institute for Cancer Design, Columbia University, New York, NY 10027, USA

[6]Massachusetts Institute of Technology, Cambridge, MA 02139, USA

[7]Paul G. Allen School of Computer Science and Engineering, University of Washington, Seattle, WA 98105, USA

[8]Graduate Program in Biological Physics, Structure and Design, University of Washington, Seattle, WA 98105, USA

[9]Centre National de la recherche scientifique, École Normale Supérieure rue d'Ulm, Paris 75005, France

[10]Department of Engineering, University of Cambridge, Cambridge CB2 1PZ, United Kingdom

[11]Howard Hughes Medical Institute, University of Washington, Seattle, WA 98105, USA

[12]School of Biological Sciences, Seoul National University, Seoul 08826, Republic of Korea

[13]Faculty of Applied Sciences, Harvard University, Cambridge, MA 01451, USA

[14]John Harvard Distinguished Science Fellowship, Harvard University, Cambridge, MA 01451 USA

[*]To whom correspondence should be addressed

[†]Equal contribution

June 2023

Contents

I Supplementary Information Figures 5
SI Figure 1 . . . 6
SI Figure 2 . . . 7
SI Figure 3 . . . 9
SI Figure 4 . . . 11
SI Figure 5 . . . 13
SI Figure 6 . . . 15
SI Figure 7 . . . 17
SI Figure 8 . . . 19
SI Figure 9 . . . 21
SI Figure 10 . . . 23
SI Figure 11 . . . 25
SI Figure 12 . . . 27

1 List of Supplementary Information Tables 30
SI Table 1 . . . 30

II Supplementary Methods 35

1 RoseTTAFold: updated architecture and training details **36**
1.1 Backbone structure representation . . . 36
1.2 RoseTTAFold architecture . . . 37
1.2.1 MSA to MSA updates . . . 41
1.2.2 MSA to Pair updates . . . 41
1.2.3 Pair to Pair updates . . . 41
1.2.4 Structure to Structure updates . . . 42
1.2.5 Structure Refinement . . . 42
1.2.6 Recycling . . . 42
1.3 RoseTTAFold losses . . . 43
1.4 RoseTTAFold training . . . 44

2 RFdiffusion: principles and formulation as a generative model of structure **45**
2.1 Diffusion probabilistic modeling background and notation . . . 46
2.2 Residue translations, forward and reverse transitions . . . 47
2.3 Residue rotations, forward and reverse transitions . . . 48
2.4 Self-conditioning in reverse process sampling . . . 53
2.5 Mean squared error loss on residue frames . . . 55
2.6 Geometric invariances and RFdiffusion . . . 57

3 RFdiffusion: methodology for controlled design **60**
3.1 Generation of oligomers with point group symmetries . . . 60
3.2 Conditional training for functional-motif scaffolding . . . 63
3.3 Fine-tuning and architecture modifications . . . 64
3.4 Guiding RFdiffusion inference with external potentials . . . 65
3.5 Tuning diversity by scaling noise at inference . . . 66

4 RFdiffusion: training and fine-tuning **67**
4.1 RFdiffusion base model . . . 67
4.2 Enzyme active site scaffolding by fine-tuning on minimal motifs . . . 72
4.3 Architectural modifications for protein-protein interaction design . . . 74
4.3.1 Protein–protein interface hotspots . . . 74
4.3.2 Secondary structure and block adjacency . . . 75
4.3.3 Fine-tuning for protein–protein interaction design . . . 76
4.4 Use of potentials for symmetric oligomers and pocket design . . . 77

5 In silico experimental methods **79**
5.1 Justification for using AlphaFold2 as an *in silico* metric . . . 79
5.2 ProteinMPNN and AlphaFold2 settings . . . 79
5.3 *In silico* success rate . . . 80
5.4 Unconditional benchmarking . . . 81
5.5 Conditional benchmarking . . . 82
5.6 Assessing diversity of designs . . . 88

5.7 Using protein BLAST to check for similar sequences in UniRef90 88
5.8 Assessing choice of losses 89
5.9 Design of fold-conditioned proteins 90
5.10 Design of symmetric oligomers 90
5.11 Design of p53 helix scaffolds 91
5.12 Design of theoretical scaffolds to enzyme active sites 92
5.13 Design of theoretical C3-symmetric spike SARS-CoV-2 spike protein binding oligomers 93
5.14 Design of symmetric Nickel binding oligomers 93
5.15 Design of protein binders to rigid targets 95
5.16 Figures and statistics shown in the paper 97
5.17 Ablations 98

6 In vitro experimental methods 101
6.1 Plasmid construction 101
6.2 Protein expression and purification 101
6.3 Negative-stain EM sample preparation 103
6.4 Negative-stain EM data collection, processing, and validation 103
6.5 CryoEM sample preparation and data collection 105
6.6 CryoEM data processing and model building 106
6.7 Circular dichroism experiments 108
6.8 Bio-layer inferometry (BLI) binding experiments 108
6.9 Comparison of experimental success rates between design campaigns 109
6.10 Isothermal titration calorimetry 110

List of Supplementary Methods Tables

1 Description of features input to RoseTTAFold. 39
2 Description of outputs returned by RoseTTAFold. 40
3 RoseTTAFold training hyperparameters. 45
4 Description of features input to RFdiffusion. 68
5 Outputs returned by RFdiffusion. 69
6 RFdiffusion training hyperparameters. 73
7 RFdiffusion input feature modified for fine-tuning on complexes. 75
8 Modified features used in fine-tuning on complexes and fold-conditioning. 75
9 A benchmarking set of recently published functional-motif scaffolding problems . . . 85
10 Functional-site scaffolding benchmark results 86
11 Scaffolding sites from orphan proteins or proteins in the training set 87
12 Training ablations for RFdiffusion. 99
13 Inference time ablations for RFdiffusion. 100
14 Model-to-density correlation of oligomers docked into EM density 104

Part I

Supplementary Information Figures

SI Figure 1

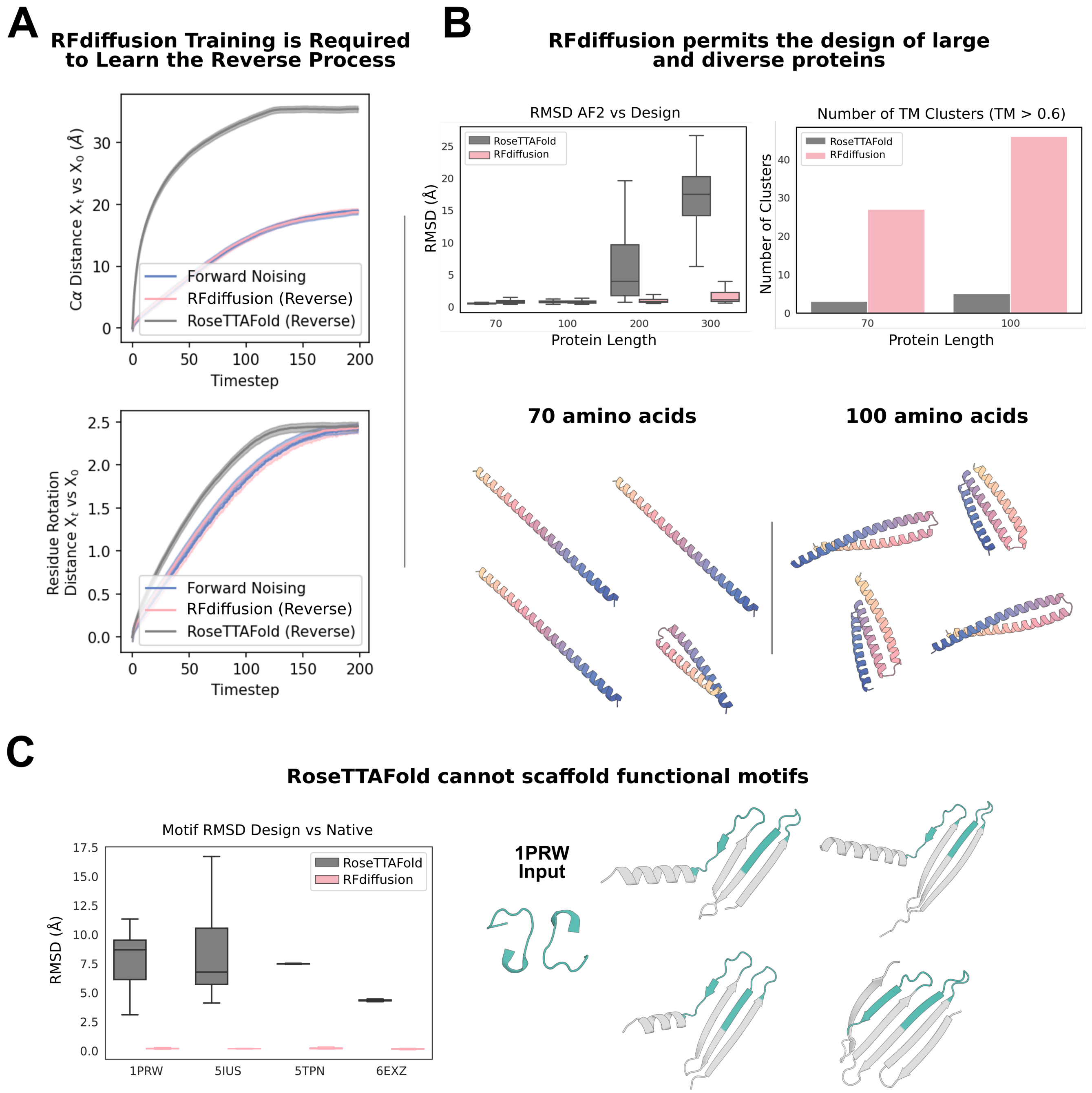
A
RFdiffusion Training is Required to Learn the Reverse Process
Cα Distance X_t vs X_0 (Å)
Timestep
Forward Noising
RFdiffusion (Reverse)
RoseTTAFold (Reverse)
Residue Rotation Distance X_t vs X_0
B
RFdiffusion permits the design of large and diverse proteins
RMSD AF2 vs Design
RMSD (Å)
Protein Length
RoseTTAFold
RFdiffusion
Number of TM Clusters (TM > 0.6)
Number of Clusters
70 amino acids
100 amino acids
C
RoseTTAFold cannot scaffold functional motifs
Motif RMSD Design vs Native
1PRW
5IUS
5TPN
6EXZ
1PRW Input

SI Figure 1: RFdiffusion fine-tuning is required to adapt RoseTTAFold into a generative model. A) The initial RoseTTAFold structure prediction network cannot perform the correct reversal of the time-dependent forward noising process. Twenty 300 amino acid unconditional designs were generated with either RoseTTAFold or RFdiffusion, and their predictions at each timestep analyzed. While RFdiffusion makes translational- and rotational-predictions (pink) that closely match the forward noising process (blue), RoseTTAFold (gray) makes inconsistent predictions. **B)** Non-finetuned RoseTTAFold does have some ability to unconditionally-generate designable backbones, although this deteriorates at lengths greater than 100 amino acids (top left). The 70 and 100 amino acid, designable designs, are not diverse however, with significantly fewer structural clusters than RFdiffusion (top right). Four randomly selected RoseTTAFold-generated designs are depicted (bottom), demonstrating this lack of structural diversity. **C)** RFdiffusion also requires fine-tuning to scaffold protein functional sites. While RFdiffusion learns to keep the functional motif fixed in the output (median RMSD on the functional motif of 0.19Å), RoseTTAFold cannot (7.45Å). This inability is shown on the right, with four randomly-selected RoseTTAFold outputs highlighting the inability to maintain the correct internal structure of the double EF-hand motif from PDB: 1PRW. Boxplot represents median±IQR; tails: min/max excluding outliers(±1.5xIQR).

SI Figure 2

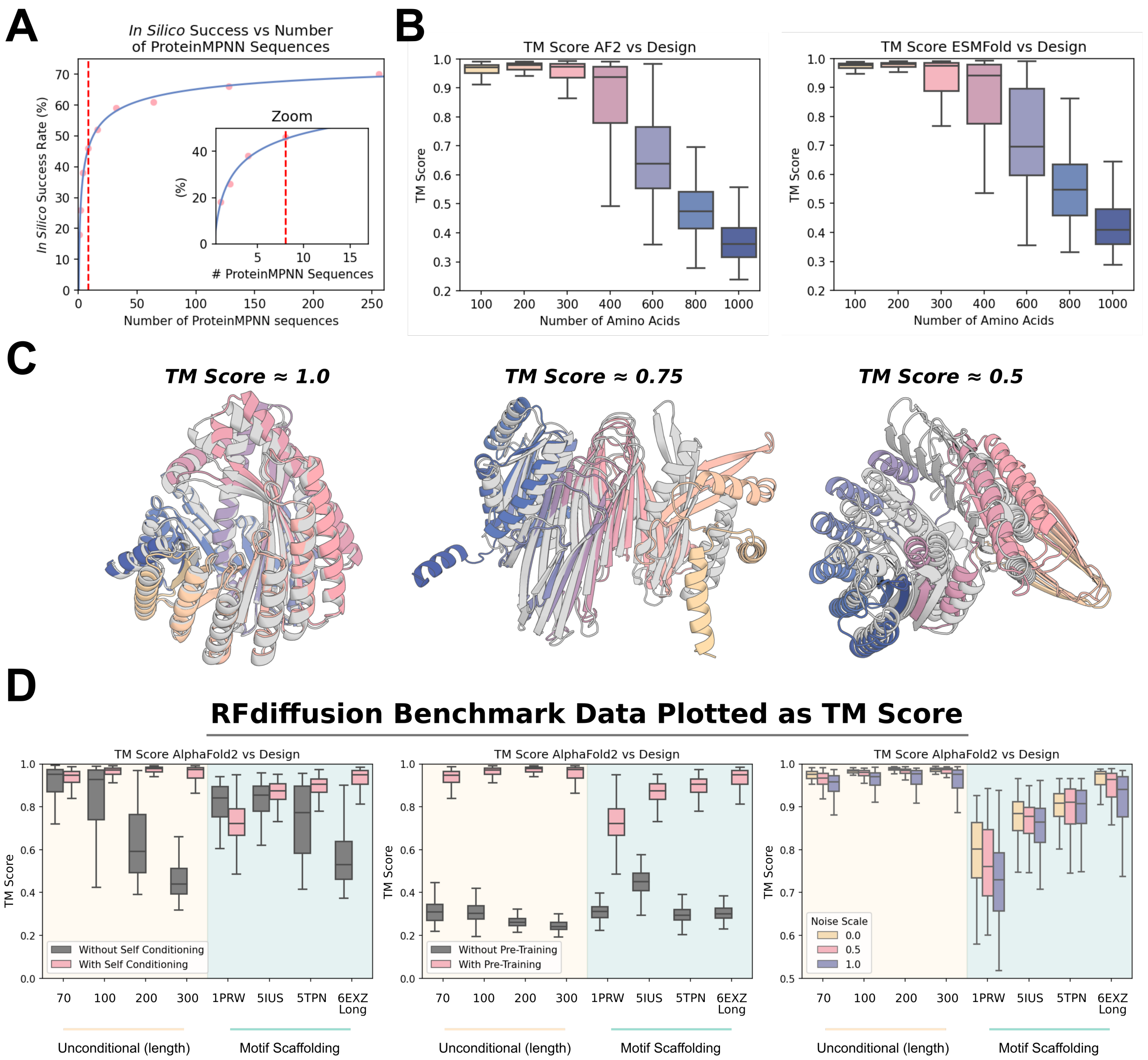

A
In Silico Success vs Number of ProteinMPNN Sequences
In Silico Success Rate (%)
Number of ProteinMPNN sequences
Zoom
(%)
ProteinMPNN Sequences
B
TM Score AF2 vs Design
TM Score ESMFold vs Design
TM Score
Number of Amino Acids
C
TM Score ≈ 1.0
TM Score ≈ 0.75
TM Score ≈ 0.5
D
RFdiffusion Benchmark Data Plotted as TM Score
TM Score AlphaFold2 vs Design
Without Self Conditioning
With Self Conditioning
Without Pre-Training
With Pre-Training
Noise Scale
1PRW
5IUS
5TPN
6EXZ Long
Unconditional (length)
Motif Scaffolding

Supplementary Information Figure 2: Improving metrics for defining *in silico* success. A) In this work, following [5, 16], we sample 8 ProteinMPNN sequences for reported benchmarks. Higher *in silico* success rates can be achieved through sampling greater numbers. Data displayed is for unconditional 300 amino acid proteins. **B-C)** TM score between a design and a subsequent orthogonal prediction (e.g. AF2), has been previously used, typically with a threshold of > 0.5, as a metric for *in silico* design success. **B)** RFdiffusion designs have high TM score agreement to both the AF2 (left) and ESMFold (right) predictions of the unconditional structures, with TM > 0.5 for a significant fraction of designs even up to 1000 amino acids in length. **C)** TM score is, however, much less stringent than RMSD alignment. Depicted here are three unconditional RFdiffusion designs of 600 amino acids in length (gray), overlaid with the AF2 prediction (colors), with TM scores of 0.983, 0.757 and 0.506 respectively. While a TM score of 0.5 clearly shows some resemblance to the designed structure, it differs significantly and should not be classed as "successfully designed". RMSD with a strict threshold (for example, 2Å) is significantly more stringent. RMSDs for the displayed designs are 1.15Å, 9.78Å and 21.4Å respectively. **D)** To permit comparison to other work, where TM score has been used, we replot three benchmarks as TM score between design and AF2 prediction. These correspond to Extended Data Fig. 1E (left), Extended Data Fig. 1F (middle), Extended Data Fig. 1I (right). Boxplots represent median±IQR; tails: min/max excluding outliers(±1.5xIQR).

SI Figure 3

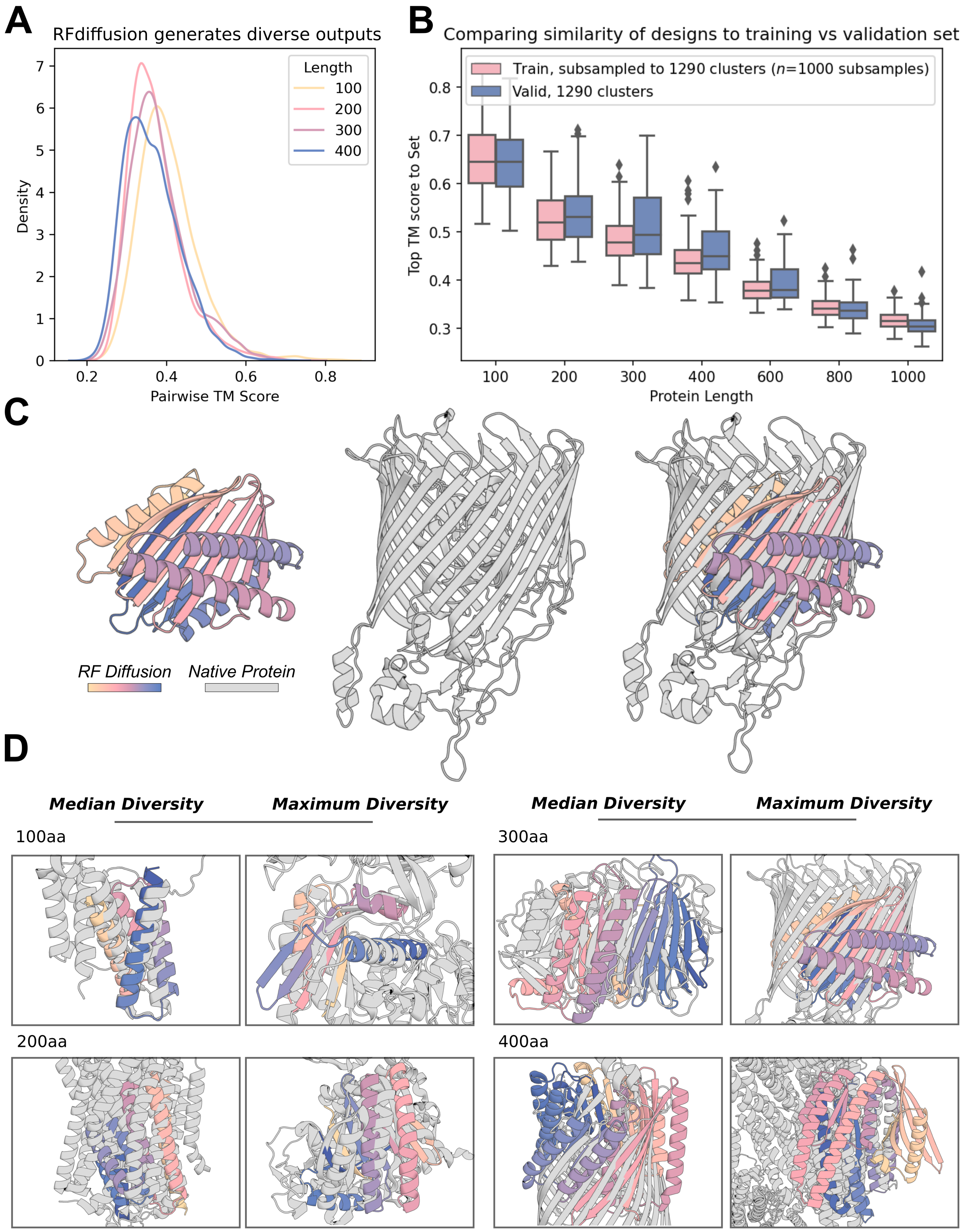
A
RFdiffusion generates diverse outputs
Length
100
200
300
400
Density
Pairwise TM Score
B
Comparing similarity of designs to training vs validation set
Train, subsampled to 1290 clusters (n=1000 subsamples)
Valid, 1290 clusters
Top TM score to Set
Protein Length
C
RF Diffusion
Native Protein
D
Median Diversity
Maximum Diversity
Median Diversity
Maximum Diversity
100aa
300aa
200aa
400aa

SI Figure 3: RFdiffusion designs are diverse and dissimilar to proteins in the PDB. A) Comparing unconditional designs to one another (100 designs per length) demonstrates that, by TM score alignment, designs are diverse (medians 100-400aa: 0.39, 0.36, 0.37, 0.35). **B)** Outputs from RFdiffusion are not systematically more similar to example structures in the training set than the validation set. The RFdiffusion (and original RF) training set is structurally distinct from the validation set. Structural comparison to the validation set, or a subsampled training set (to normalize for the total number of TM alignments performed) shows that RFdiffusion outputs show comparable similarity to each set (ANOVA with Tukey post-hoc test, n = 100 designs per condition, mean difference between sets = 0.0065, p = 0.32). 1000 unique training set subsamples are averaged. We therefore conclude that any structural similarity seen to proteins in the PDB is as a result of learning the distribution of native-like proteins, rather than because of memorization. Further, the greater similarity (higher TM score) of short proteins is similarly not due to memorization, but instead the smaller space of possible protein folds for smaller sequences. **C)** Example of the most diverse (lowest TM score hit) to the PDB for a set of 300 amino acid designs. The folds of the design (left) and native protein (middle) are highly dissimilar, aligning only across a portion of the beta-sheet (right). **D)** Additional example designs demonstrating extrapolation beyond the training set for generating novel folds. Gray: closest protein in the PDB by TM score, colors: RFdiffusion design model, overlaid by TM alignment. For each protein length, the median and most diverse samples are shown (the 300 amino acid design is the same as in panel **C)**. While for short proteins, designs typically show some similarity to known protein folds, with increasing length, designs become increasingly dissimilar to the PDB. TM score (closest PDB, TM score; median, most diverse): 100aa: 5WVE_A, 0.71; 4W5T_A, 0.59; 200aa: 4AV3_A, 0.58; 4CLY_A, 0.47; 300aa: 4PEW_B, 0.53; 4RDR_A, 0.46; 400aa: 4AIP_A, 0.49; 6R9T_A, 0.42. Boxplot represents median±IQR; tails: min/max excluding outliers(±1.5xIQR).

SI Figure 4

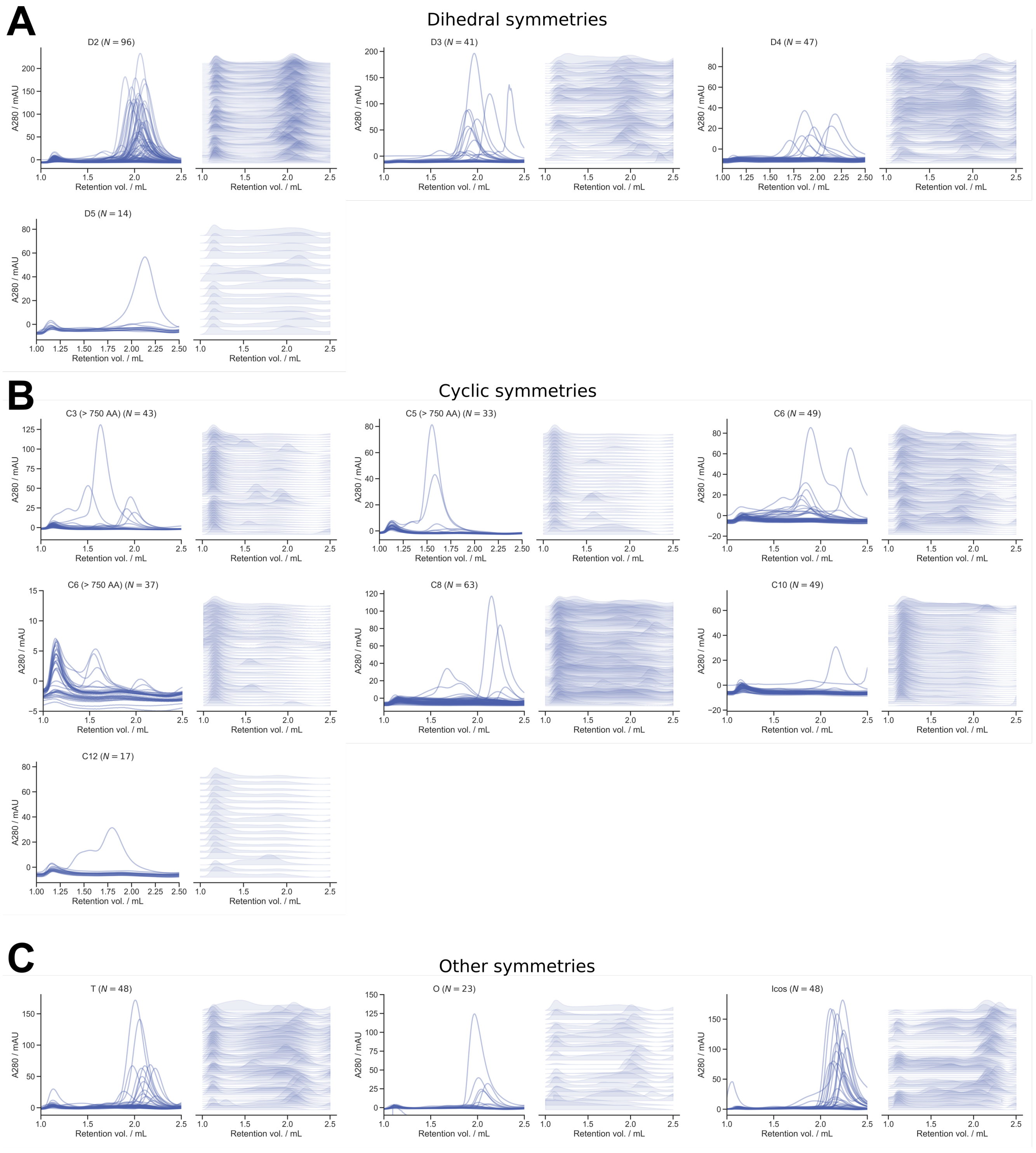

A
Dihedral symmetries
D2 (N = 96)
D3 (N = 41)
D4 (N = 47)
D5 (N = 14)
B
Cyclic symmetries
C3 (> 750 AA) (N = 43)
C5 (> 750 AA) (N = 33)
C6 (N = 49)
C6 (> 750 AA) (N = 37)
C8 (N = 63)
C10 (N = 49)
C12 (N = 17)
C
Other symmetries
T (N = 48)
O (N = 23)
Icos (N = 48)
A280 / mAU
Retention vol. / mL

Supplementary Information Figure 4: Size exclusion chromatography of symmetric oligomers. A-C) Size exclusion chromatography (SEC) was used as a primary screening method for all RFdiffusion-generated oligomers. Here, SEC traces from 608 oligomers are shown for each of the experimentally tested symmetry groups, excluding the void volume. Panel **A)** shows dihedral symmetries, **B)** shows cyclic symmetries, and **C)** shows all others. For each set of traces, on the left, data are overlaid for all designs, and on the right, traces are normalized and stacked. As designs increase in complexity (higher number of individual subunits), the amount of soluble protein shown by SEC visibly decreases. For tetrahedral, octahedral, and icosahedral designs, many have soluble protein peaks that are possibly dimer and trimer subunits (unassembled cages).

SI Figure 5

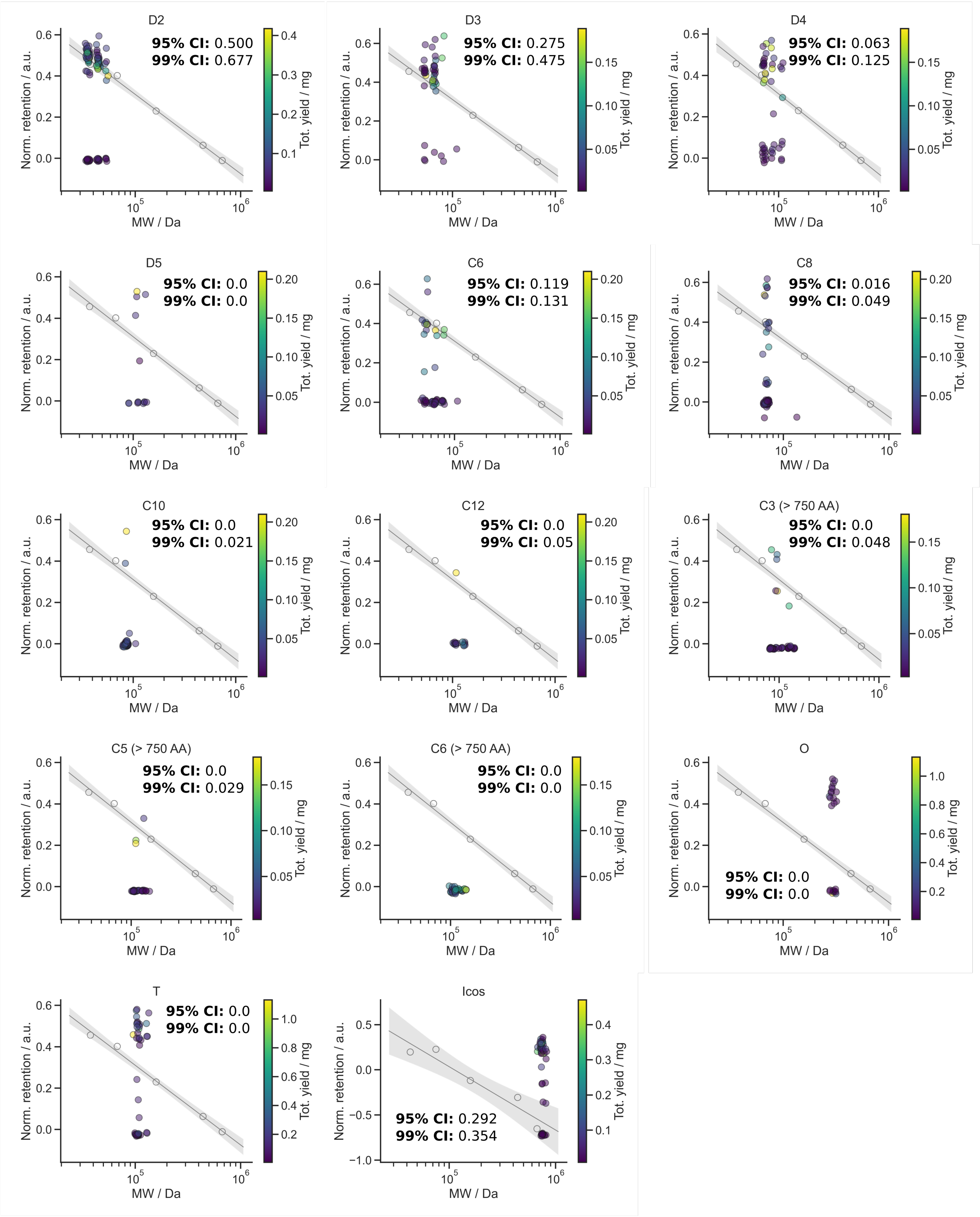
D2
95% CI: 0.500
99% CI: 0.677
D3
95% CI: 0.275
99% CI: 0.475
D4
95% CI: 0.063
99% CI: 0.125
D5
95% CI: 0.0
99% CI: 0.0
C6
95% CI: 0.119
99% CI: 0.131
C8
95% CI: 0.016
99% CI: 0.049
C10
95% CI: 0.0
99% CI: 0.021
C12
95% CI: 0.0
99% CI: 0.05
C3 (> 750 AA)
95% CI: 0.0
99% CI: 0.048
C5 (> 750 AA)
95% CI: 0.0
99% CI: 0.029
C6 (> 750 AA)
95% CI: 0.0
99% CI: 0.0
O
95% CI: 0.0
99% CI: 0.0
T
95% CI: 0.0
99% CI: 0.0
Icos
95% CI: 0.292
99% CI: 0.354
Norm. retention / a.u.
MW / Da
Tot. yield / mg

Supplementary Information Figure 5: SEC elution peaks of symmetric oligomers vs. calibration curves. Retention volume for the major SEC peak versus molecular weight for each design are plotted in comparison to a known calibration curve. The calibration curve is shown in gray, with shading representing the 95% confidence interval. Total yield of each design is indicated by the scale bar on the right of the graphs, and success rates for the 95% CI and 99% CI are denoted on each graph per each symmetry. Given that MW is being used as a proxy for hydrodynamic radius, we expect that some designs (e.g. cycles with large pores) may be true to their design model, but deviate from the standard curve. These calibration curves provide a rough estimate of the success rate of each symmetry group, and help guide the selection process for downstream analysis of any design. In some cases, even though no designs are within the 99% CI, we still selected designs to screen by nsEM. For example, we are able to confirm HE0822 (C3) by nsEM despite misalignment between the theoretical and actual elution profiles (Fig. 3B). Because of their size, the icosahedra were run on an S6 column with lower resolution; thus, the calibration curve fit results in bigger confidence intervals compared to an S200 column, which was used to screen all other oligomers (See Methods 6.2). We expect that for oligomers run on the S200, reported success rates are fairly conservative, whereas for designs run on the S6, experimental success rates are likely lower than reported.

SI Figure 6

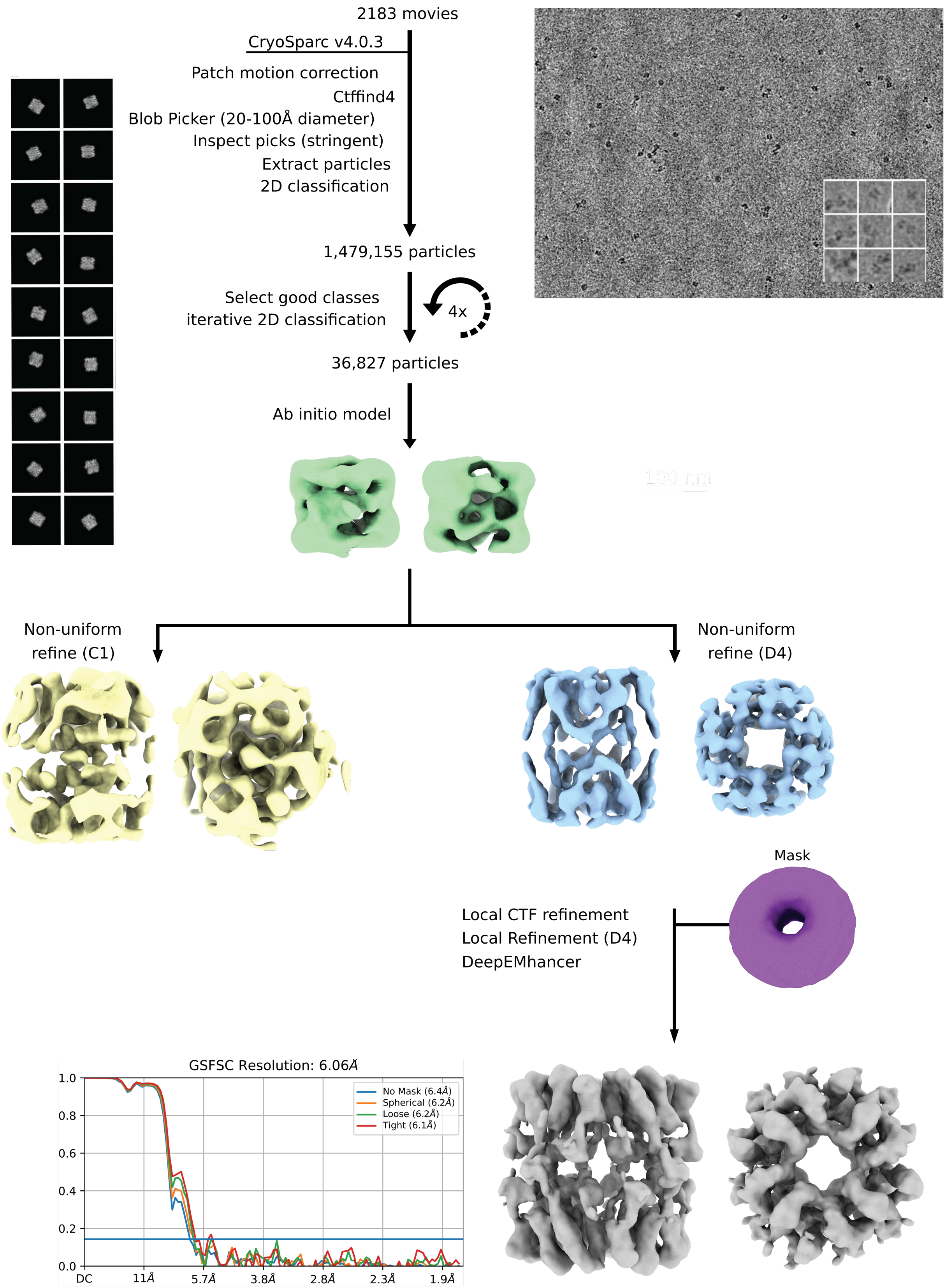
2183 movies
CryoSparc v4.0.3
Patch motion correction
Ctffind4
Blob Picker (20-100Å diameter)
Inspect picks (stringent)
Extract particles
2D classification
1,479,155 particles
Select good classes
iterative 2D classification
4x
36,827 particles
Ab initio model
100 nm
Non-uniform
refine (C1)
Non-uniform
refine (D4)
Mask
Local CTF refinement
Local Refinement (D4)
DeepEMhancer
GSFSC Resolution: 6.06Å
No Mask (6.4Å)
Spherical (6.2Å)
Loose (6.2Å)
Tight (6.1Å)
1.0
0.8
0.6
0.4
0.2
0.0
DC
11Å
5.7Å
3.8Å
2.8Å
2.3Å
1.9Å

Supplementary Information Figure 6: Details of HE0537 cryo-EM data processing pipeline. 2D class averages showing exclusively side-views of HE0537, and an *ab initio* reconstruction followed by a C1 non-uniform refinement yielding identifiable D4 features corresponding to the size and rough secondary structure of the design model. Further data processing was attempted with D4 symmetry imposed, but the strong preferred orientation precluded generation of a reliable 3D map for detailed structural analysis. At this time, only the predicted 2D projection images of the design model are analyzed/compared alongside the corresponding experimental cryo-EM 2D class average side views in Extended Data Fig. 5G, which display strikingly high agreement to the design. A representative raw cryo-EM micrograph is shown on the right along with nine example extracted particles and characteristic 2D class averages used in the processing pipeline. An FSC validation curve for the final reconstruction is shown along with the density map.

SI Figure 7

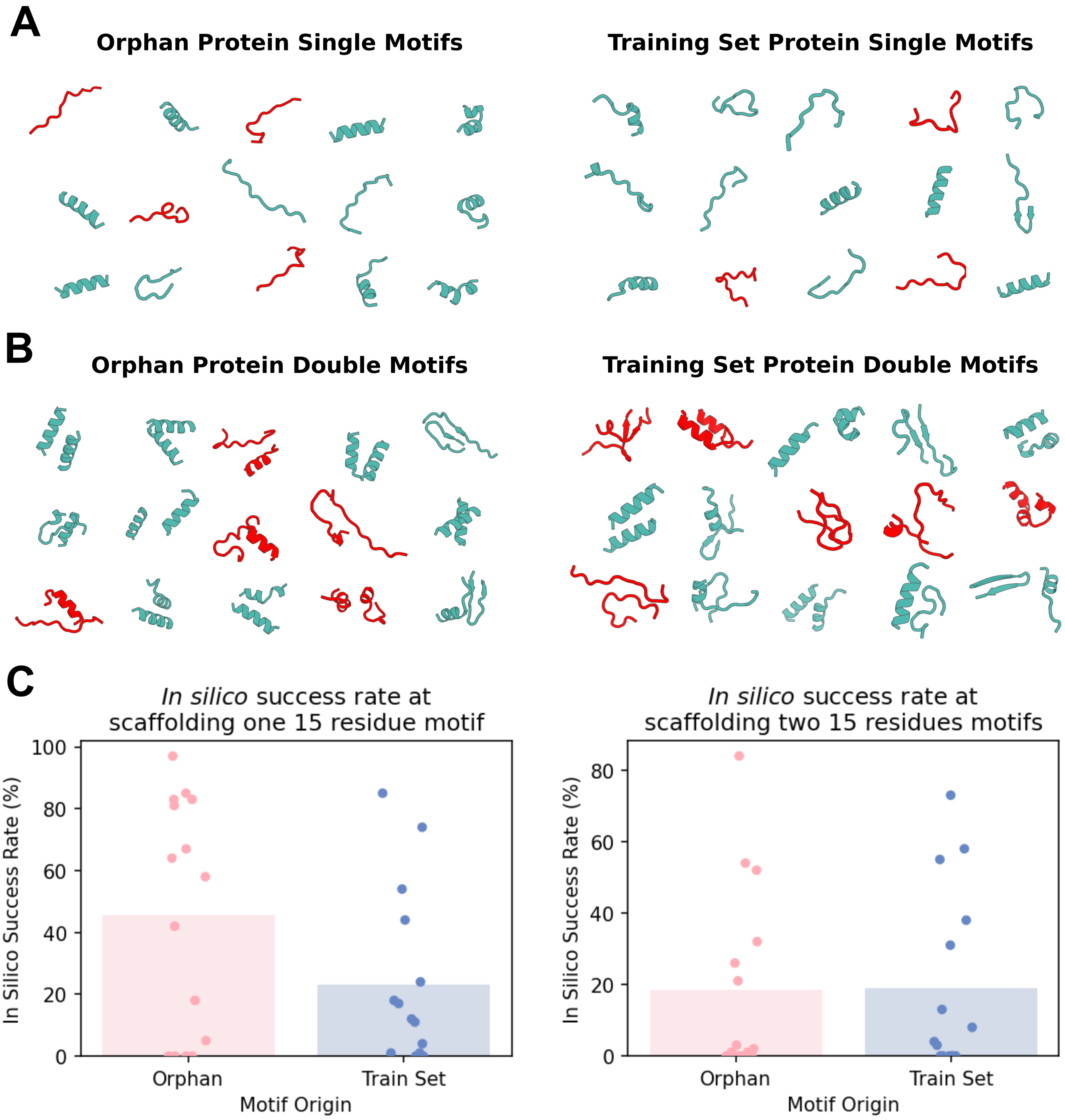

A
Orphan Protein Single Motifs
Training Set Protein Single Motifs
B
Orphan Protein Double Motifs
Training Set Protein Double Motifs
C
In silico success rate at scaffolding one 15 residue motif
In silico success rate at scaffolding two 15 residues motifs
In Silico Success Rate (%)
0
20
40
60
80
100
Orphan
Train Set
Motif Origin

Supplementary Information Figure 7: RFdiffusion can scaffold never-seen-before motifs. To test whether the ability to scaffold "motifs" was related to their presence in the training set, we randomly extracted "motifs" (either single contiguous 15 residue segments, or two discontinuous 15 residue segments close in 3D space) from two sources. The first set represents the 15 orphan proteins described in Wu et al. [20], which lack sequence or structure homology to known proteins, and are in neither the RF, RFdiffusion or AF2 training sets The second set were randomly sampled from the RF (and RFdiffusion) training set. Full details of the structures and "motif" regions are described in Supplementary Methods Table 9, along with tabulated *in silico* results in Supplementary Methods Table 10. **A-B)** Illustrations of the "motifs" randomly selected for scaffolding. Teal: motifs that RFdiffusion can successfully scaffold *in silico* (AF2 pAE < 5, RMSD AF2 vs Design < 2Å, Motif RMSD vs Native < 1Å). Red: motifs that RFdiffusion failed to scaffold in silico. **C)** The success rate on both single and double "motifs" is at least as high for "orphan" motifs as compared to "motifs" from the training dataset. Note that while RFdiffusion failures were generally loop-rich, RFdiffusion is able to scaffold "motifs" of a broad range of topologies and secondary structures. Success rates are typically lower for discontiguous motifs as compared to single motifs.

SI Figure 8

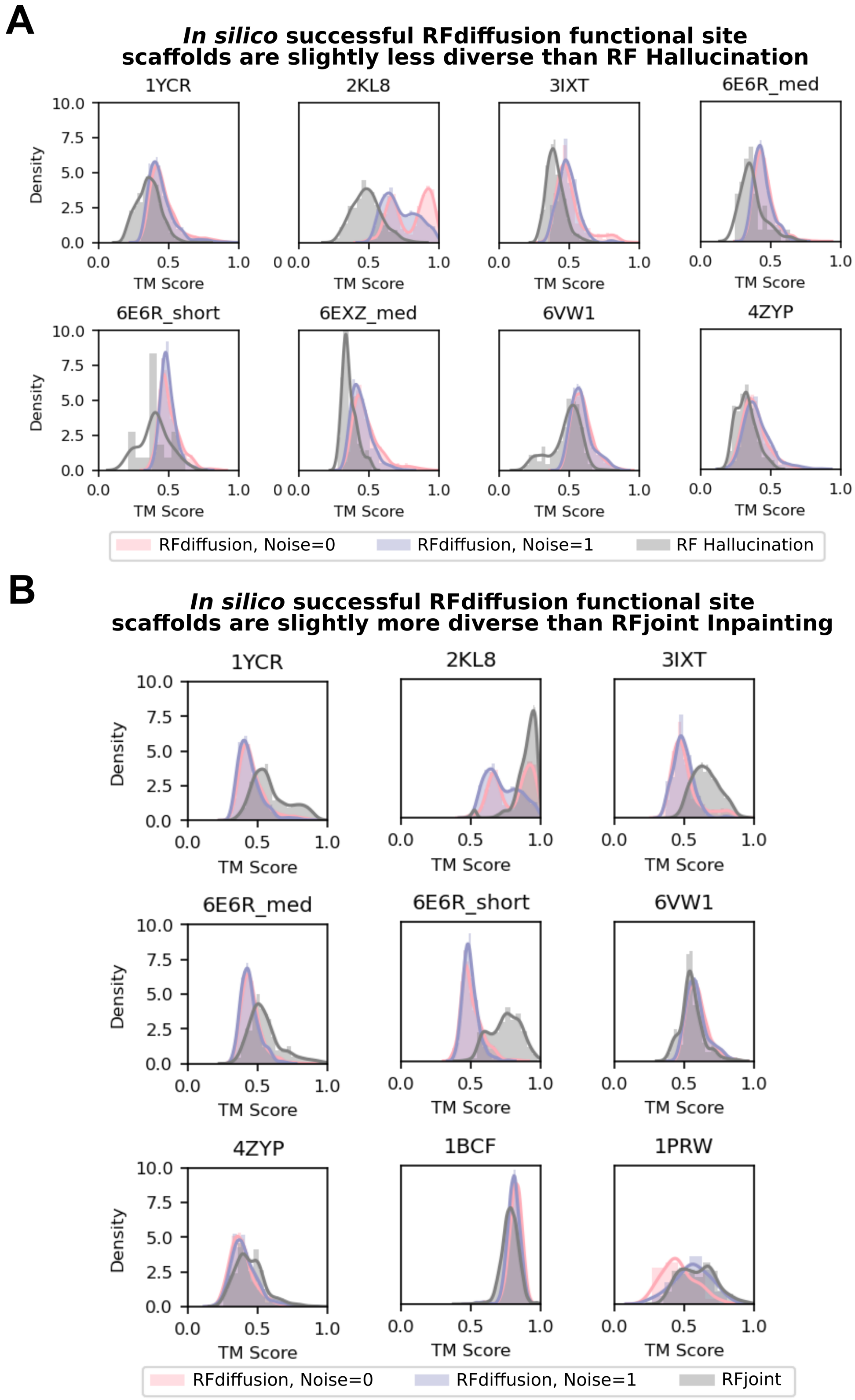
A
In silico successful RFdiffusion functional site scaffolds are slightly less diverse than RF Hallucination
1YCR
2KL8
3IXT
6E6R_med
6E6R_short
6EXZ_med
6VW1
4ZYP
Density
TM Score
RFdiffusion, Noise=0
RFdiffusion, Noise=1
RF Hallucination
B
In silico successful RFdiffusion functional site scaffolds are slightly more diverse than RFjoint Inpainting
1YCR
2KL8
3IXT
6E6R_med
6E6R_short
6VW1
4ZYP
1BCF
1PRW
Density
TM Score
RFdiffusion, Noise=0
RFdiffusion, Noise=1
RFjoint

Supplementary Information Figure 8: Comparing the diversity of functional motif scaffolds. The diversity of scaffolds produced by the three "motif-scaffolding" methods; RFdiffusion, RF_{joint} Inpainting and RF Hallucination was compared by pairwise TM score analysis of the designed scaffolds (Methods 5.6). Only scaffolding problems with 5 or more *in silico* successes are plotted, to permit meaningful comparison. **A)** RFdiffusion *in silico* successes are typically somewhat less diverse than (the smaller number of) *in silico* successes generated by RF Hallucination. **B)** RFdiffusion *in silico* successes are generally more diverse than *in silico* successes generated by RF_{joint} inpainting, where diversity comes solely from the sampling of different scaffold lengths.

SI Figure 9

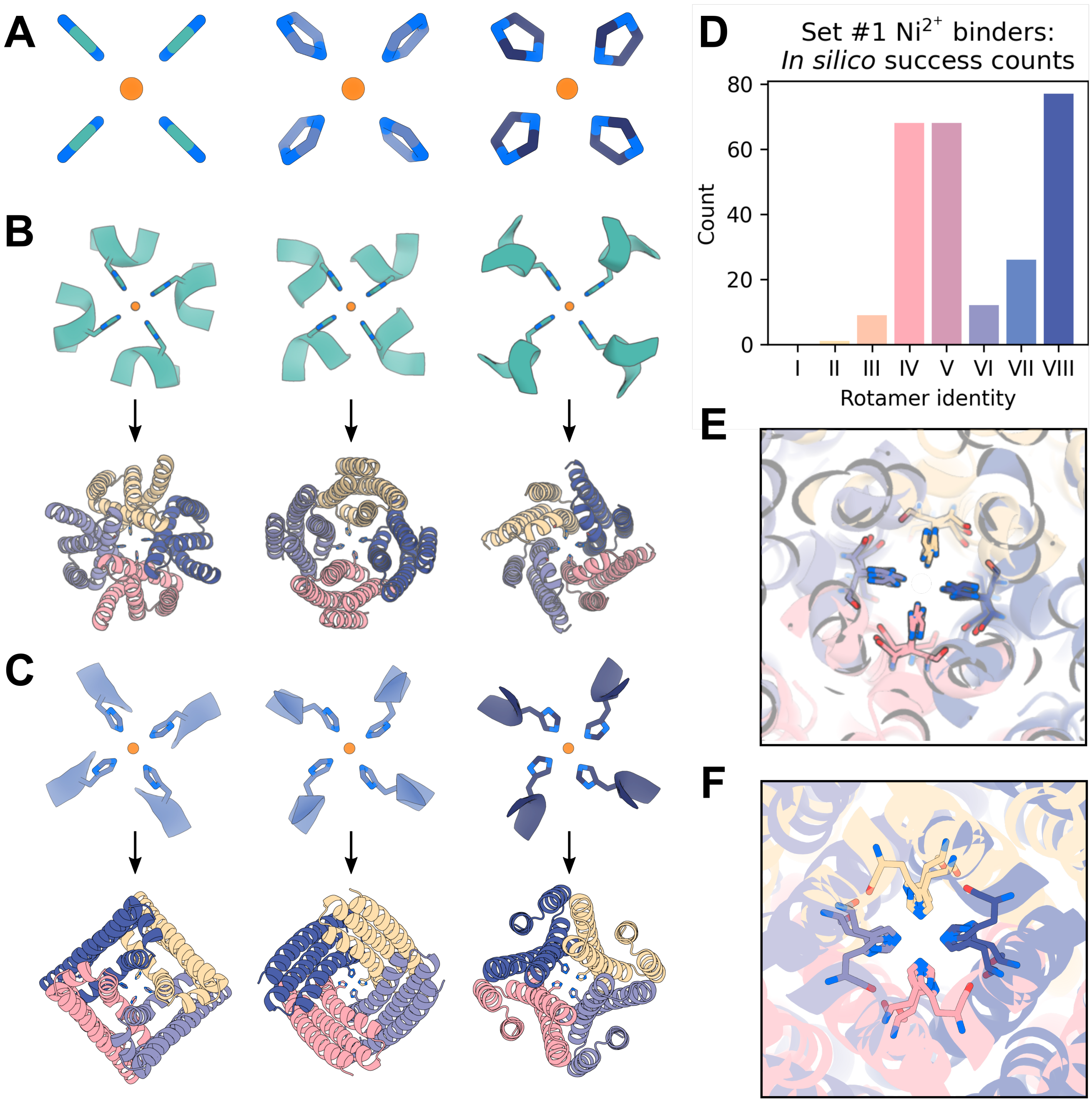
A
B
C
D
Set #1 Ni2+ binders:
In silico success counts
Count
80
60
40
20
0
I
II
III
IV
V
VI
VII
VIII
Rotamer identity
E
F

Supplementary Information Figure 9: Symmetric motif scaffolding for square-planar Ni^{2+} binding. A) Symmetrized imidazole groups of varying shear angles used for constructing the square-planar motifs to scaffold, with 2.2Å between the theoretically Ni^{2+} coordinating nitrogen and the symmetry axis. **B**) Depiction of a subset of the C4-symmetrized backbone-dependent ($\phi = -40^o, \psi = -60^o$) rotamers [59] ("inverse rotamers", Methods 5.9) used as motifs from set 1 input to RFdiffusion for symmetrically scaffolding the theoretical Ni2+ binding site (teal, top). AF2 predictions of selected *in silico* successes scaffolding the C4 inverse rotamers show significant structural diversity in RFdiffusion solutions (colors, bottom). All AF2 structures have full-atom RMSD < 1.0Å between AF2 predictions and the input motif, AF2 pAE < 6, and AF2 plDDT $>$ 90. **C)** Depiction of a different subset of the C4-symmetrized backbone-dependent ($\phi = -40^o, \psi = -60^o$) inverse rotamers [59] used as motifs from set 2 (top), with AF2 predictions of selected *in silico* successes (bottom). All AF2 structures have full-atom RMSD < 1.0Å between AF2 predictions and the input motif, AF2 pAE < 6, AF2 plDDT > 90 **D)** In silico success count for the inverse rotamers from set 1 depicted in panel B. An *in silico* "success" here is defined in accordance with success on oligomers and success on the active site scaffolding. That is, an AF2 prediction for a single sequence which has (1) full-atom RMSD over the four histidine residues between the AF2 prediction and the ideal motif of < 1.5Å (2) AF2 pLDDT > 80, (3) backbone RMSD between AF2 and the ideal motif < 1.0Å and (4) backbone RMSD between AF2/design over the entire protein $<$ 2.0Å (Methods 5.3). **E)** Overlay of various AF2 predictions for designs scaffolding motifs derived from imidazole groups with no shear (panel A, left) shows a diverse array of RFdiffusion solutions can all place the histidine imidazole groups at near-ideal distances from a theoretical nickel ion. **F)** Overlay of various AF2 predictions for motifs derived from imidazole groups with shear (panel A, middle and right) again displays diverse backbone solutions for placing the imidazole groups at near-ideal distances from the theoretical Ni^{2+} ion.

SI Figure 10

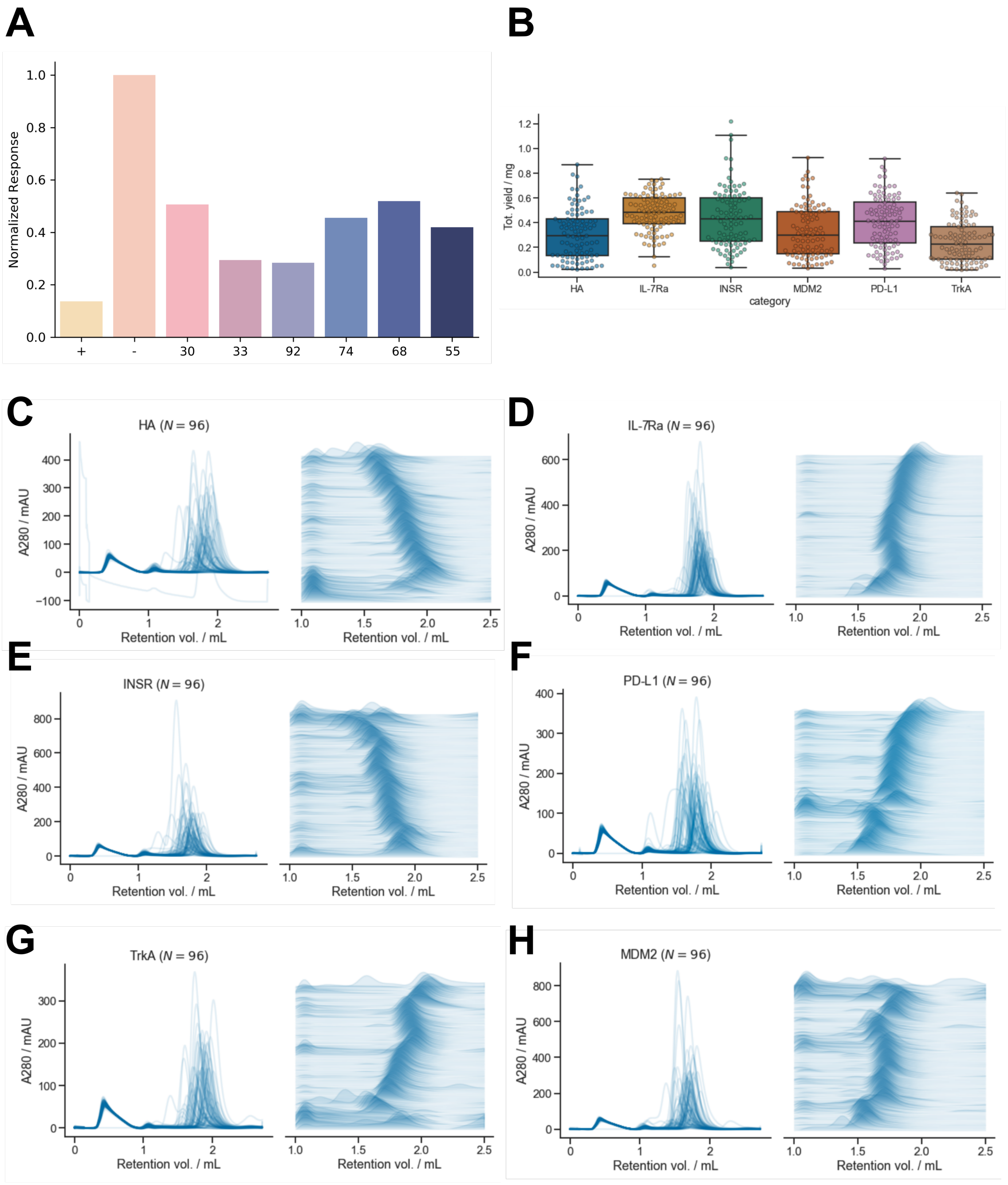

A
B
C
D
E
F
G
H
Normalized Response
Tot. yield / mg
category
HA
IL-7Ra
INSR
MDM2
PD-L1
TrkA
HA (N = 96)
IL-7Ra (N = 96)
INSR (N = 96)
PD-L1 (N = 96)
TrkA (N = 96)
MDM2 (N = 96)
A280 / mAU
Retention vol. / mL

Supplementary Information Figure 10: Additional Characterization of Binder Designs. **A)** IL-7Rα Competition Assay. Positive control (known IL-7Rα binder from ref [12]) was amine conjugated to ar2g biosensor tips. 100nM IL-7Rα with 1μM of each design then was used as analyte. Positive control was also included as an analyte as there should be no binding. Response is normalized to binding of IL-7Rα on its own. All six diffusion-generated binders compete with the positive control, indicating they bind to the intended site. **B)** Most binders are expressed with high yield in E. coli. **C-H)** SEC elution profiles indicate most binders elute as monomers. In order: Influenza Hemagglutinin, IL-7Rα, Insulin Receptor, PD-L1, TrkA, Mdm2 (p53 scaffolds).

SI Figure 11

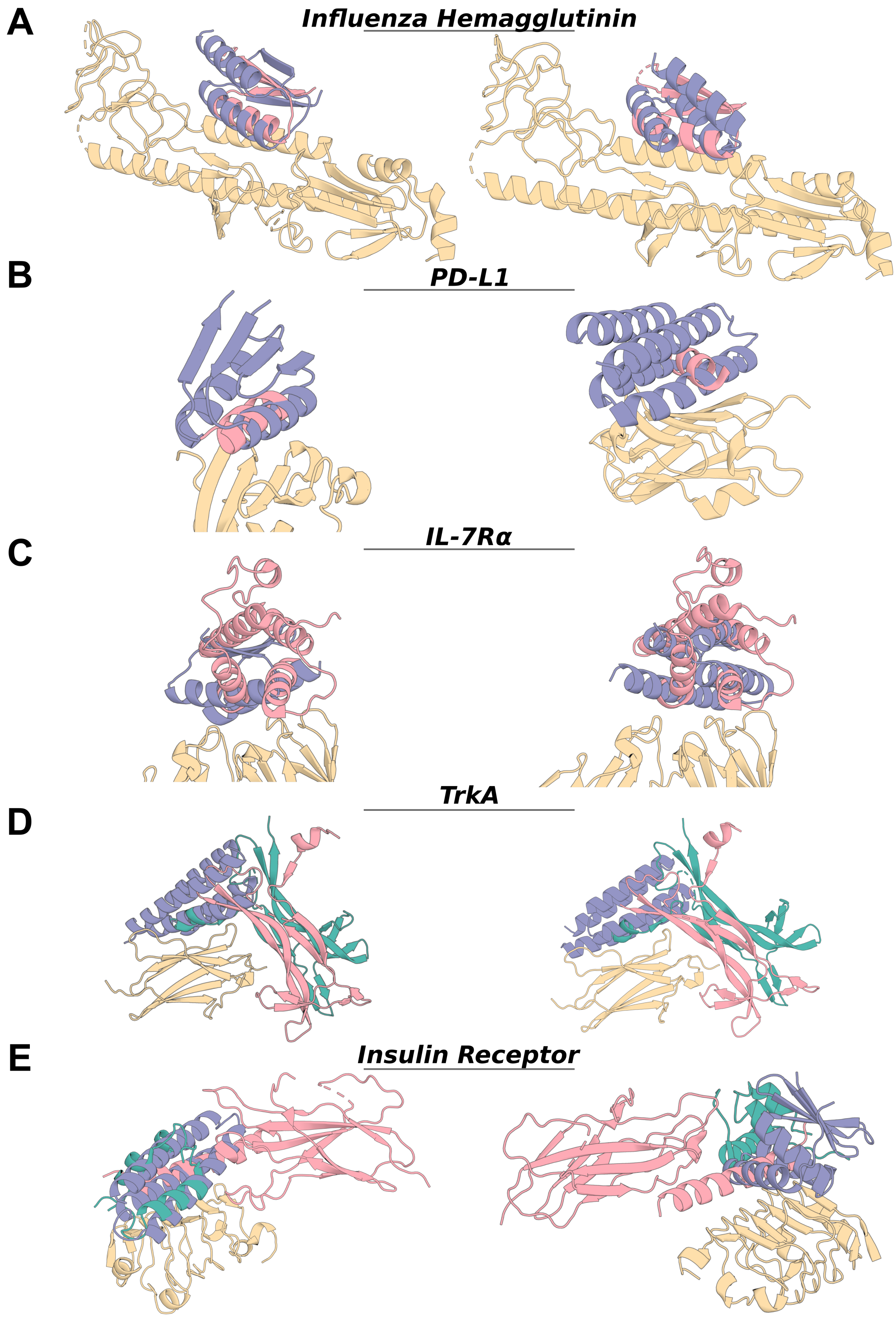
A
Influenza Hemagglutinin
B
PD-L1
C
IL-7Rα
D
TrkA
E
Insulin Receptor

Supplementary Information Figure 11: Alignment of RFdiffusion binder structures to complexes in the training set. For all targets the AF2 model of the highest affinity RFdiffusion binder (left, blue) and another experimentally-validated RFdiffusion binder with a different dock (right, blue) is shown in complex with its target (yellow). The closest matching interface, as determined by manual inspection of PDB entries with the same target protein name are aligned to the designed binder. **A)** Influenza HA binders aligned to PDB 5VLI (pink). **B)** PD-L1 binders aligned to PDB 7UXO (pink). **C)** IL-7Rα binders aligned to PDB 3DI2 (pink). **D)** TrkA binders aligned to PDB 2IFG_E (pink) and 2IFG_F (teal). **E)** Insulin Receptor binders aligned to PDB 6PXV_C (pink) and 6PXV_D (teal).

SI Figure 12

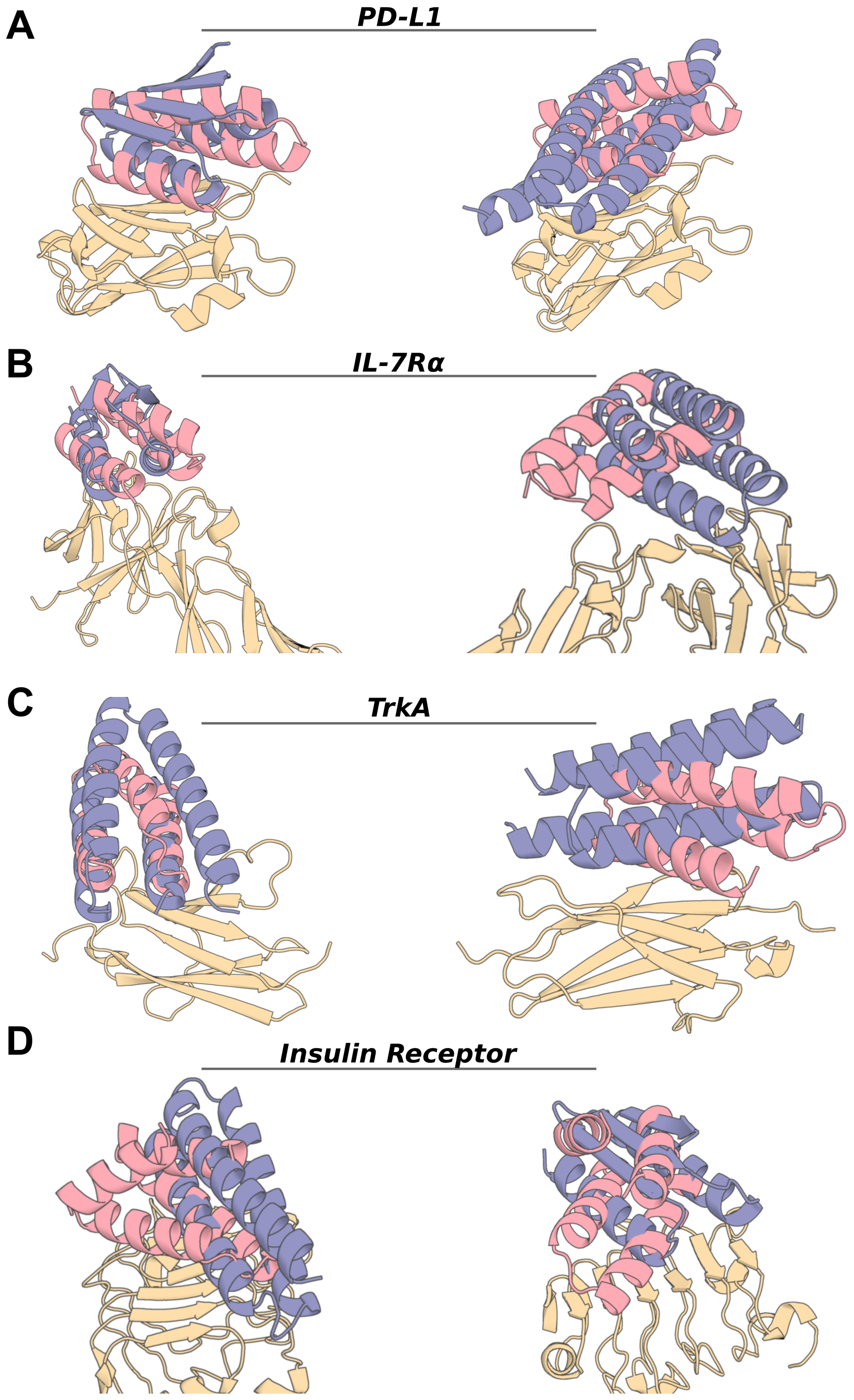

Supplementary Information Figure 12: Alignment of RFdiffusion binder structures to previously designed binders. The AF2 model of the highest affinity RFdiffusion binder (left, blue) and another experimentally-validated RFdiffusion binder with a different dock (right, blue) is shown in complex with its target (yellow). The structure of the final affinity-matured binder from Cao et al. [12] is shown in pink. The final affinity-matured binder to Influenza HA was in the training set and is shown in Extended Data Fig. 9H and omitted here. **A)** PD-L1 binder aligned to Rosetta design model of the affinity-matured PD-L1 binder from Cao et al. **B)** IL-7Rα binder aligned to the crystal structure of the affinity-matured IL-7Rα binder from Cao et al. (7OPB). **C)** TrkA binder aligned to the crystal structure of the affinity-matured TrkA binder from Cao et al. [12] (7N3T). **D)** Insulin receptor binder aligned to the Rosetta design model of the affinity-matured Insulin receptor binder from Cao et al [12]. The four previous de novo binders were not in the RF or RFdiffusion training set.

1 List of Supplementary Information Tables

SI Table 1

Dataset	Associated Figures	Description of analyzed dataset	(TMalign) Median of highest TM Score to PDB	(TMalign) 5th, 25th, 75th, 95th percentile highest TMscore to PDB	(TMalign) Median pairwise TM score within filtered designs	(TMalign) 5th, 25th, 75th, 95th percentile pairwise TM score	(BLAST) Fraction with significant blast hits (E-value < 0.1) in UniRef90	(BLAST) Query SeqID for best UniRef90 hit (5th/25th/50th/75th/95th percentiles)
p53 Helix Scaffolds	Fig. 4C-E	Set of 95 tested designs, AlphaFold2 models	0.645	0.588 / 0.623 / 0.690 / 0.735	0.406	0.333 / 0.371 / 0.455 / 0.547	0.073	0.219/0.232/0.262/0.296/0.316
IL-7Ra binders	Fig. 6	Set of 95 tested designs, AlphaFold2 models	0.793	0.708 / 0.754 / 0.839 / 0.901	0.483	0.370 / 0.428 / 0.544 / 0.665	0.124	0.2/0.289/0.3/0.329/0.383
Insulin binders	Fig. 6	Set of 95 tested designs, AlphaFold2 models	0.801	0.703 / 0.759 / 0.838 / 0.878	0.467	0.350 / 0.405 / 0.538 / 0.669	0.229	0.224/0.289/0.318/0.344/0.403
TrkA binders	Fig. 6	Set of 95 tested designs, AlphaFold2 models	0.794	0.691 / 0.753 / 0.827 / 0.893	0.447	0.348 / 0.399 / 0.524 / 0.705	0.198	0.252/0.3/0.333/0.356/0.405
Influenza Hemagglutinnin binders	Fig. 6	Set of 95 tested designs, AlphaFold2 models	0.807	0.722 / 0.753 / 0.839 / 0.875	0.469	0.368 / 0.421 / 0.536 / 0.677	0.083	0.262/0.28/0.31/0.34/0.352
PD-L1 binders	Fig. 6	Set of 95 tested designs, AlphaFold2 models	0.804	0.717 / 0.763 / 0.829 / 0.867	0.479	0.349 / 0.413 / 0.546 / 0.708	0.125	0.237/0.264/0.282/0.302/0.348
Nickel-binding Oligomers (monomer)	Fig. 5	Set of 34 unique tested backbones, AlphaFold2 models. Monomer extracted from the C4 symmetric model	0.773	0.688 / 0.722 / 0.809 / 0.836	0.495	0.394 / 0.448 / 0.596 / 0.715	0.115	0.228/0.286/0.301/0.335/0.35
Nickel-binding Oligomers (oligomers)	Fig. 5	Set of 34 unique tested backbones, AlphaFold2 models. Full complex TM aligned	0.511	0.412 / 0.489 / 0.526 / 0.555	0.395	0.275 / 0.327 / 0.531 / 0.733	NA (see monomer)	NA (see monomer)
Symmetric Oligomer (C3, > 750 amino acids) - Monomer	Ext. Data Fig. 5B	Set of 20 unique tested backbones, AlphaFold2 models. Monomer extracted from oligomer	0.519	0.449 / 0.47 / 0.603 / 0.651	0.294	0.223 / 0.258 / 0.360 / 0.548	0.45	0.129/0.18/0.21/0.251/0.283
Symmetric Oligomer (C5, > 750 amino acids) - Monomer	Ext. Data Fig. 5B	Set of 17 unique tested backbones, AlphaFold2 models. Monomer extracted from oligomer	0.583	0.512 / 0.551 / 0.605 / 0.678	0.372	0.291 / 0.329 / 0.418 / 0.572	0.176	0.162/0.186/0.217/0.256/0.288
Symmetric Oligomer (C6) - Monomer	Ext. Data Fig. 5B	Set of 39 unique tested backbones, AlphaFold2 models. Monomer extracted from oligomer	0.727	0.665 / 0.697 / 0.781 / 0.887	0.445	0.334 / 0.384 / 0.541 / 0.678	0.128	0.273/0.325/0.338/0.35/0.363
Symmetric Oligomer (C6, > 750 amino acids) - Monomer	Ext. Data Fig. 5B	Set of 14 unique tested backbones, AlphaFold2 models. Monomer extracted from oligomer	0.598	0.538 / 0.545 / 0.629 / 0.72	0.338	0.234 / 0.294 / 0.434 / 0.603	0.286	0.164/0.18/0.19/0.203/0.227
Symmetric Oligomer (C8) - Monomer	Ext. Data Fig. 5B	Set of 35 unique tested backbones, AlphaFold2 models. Monomer extracted from oligomer	0.73	0.68 / 0.708 / 0.755 / 0.901	0.432	0.352 / 0.391 / 0.500 / 0.689	0.086	0.317/0.317/0.317/0.317/0.317
Symmetric Oligomer (C10) - Monomer	Ext. Data Fig. 5B	Set of 20 unique tested backbones, AlphaFold2 models. Monomer extracted from oligomer	0.705	0.599 / 0.668 / 0.722 / 0.742	0.54	0.266 / 0.430 / 0.630 / 0.714	0.05	0.417/0.417/0.417/0.417/0.417
Symmetric Oligomer (C12) - Monomer	Ext. Data Fig. 5B	Set of 9 unique tested backbones, AlphaFold2 models. Monomer extracted from oligomer	0.708	0.679 / 0.698 / 0.731 / 0.854	0.435	0.355 / 0.407 / 0.493 / 0.681	0	NA - no hits
Symmetric Oligomer (D2) - Monomer	Ext. Data Fig. 5B	Set of 84 unique tested backbones, AlphaFold2 models. Monomer extracted from oligomer	0.777	0.651 / 0.735 / 0.822 / 0.915	0.458	0.350 / 0.408 / 0.516 / 0.632	0.095	0.271/0.297/0.308/0.414/0.449
Symmetric Oligomer (D3) - Monomer	Ext. Data Fig. 5B	Set of 26 unique tested backbones, AlphaFold2 models. Monomer extracted from oligomer	0.763	0.692 / 0.738 / 0.812 / 0.85	0.506	0.401 / 0.453 / 0.553 / 0.652	0.231	0.264/0.271/0.294/0.322/0.344
Symmetric Oligomer (D4) - Monomer	Ext. Data Fig. 5B	Set of 30 unique tested backbones, AlphaFold2 models. Monomer extracted from oligomer	0.749	0.667 / 0.707 / 0.792 / 0.846	0.425	0.308 / 0.376 / 0.477 / 0.563	0.1	0.335/0.342/0.35/0.392/0.425
Symmetric Oligomer (D5) - Monomer	Ext. Data Fig. 5B	Set of 7 unique tested backbones, AlphaFold2 models. Monomer extracted from oligomer	0.738	0.69 / 0.717 / 0.748 / 0.891	0.423	0.257 / 0.342 / 0.480 / 0.574	0	NA - no hits
Symmetric Oligomer (C3, > 750 amino acids) - Full oligomer	Ext. Data Fig. 5B	Set of 20 unique tested backbones, AlphaFold2 models. Full complex	0.368	0.325 / 0.35 / 0.424 / 0.551	0.27	0.218 / 0.246 / 0.320 / 0.471	NA (see monomer)	NA (see monomer)
Symmetric Oligomer (C5, > 750 amino acids) - Full oligomer	Ext. Data Fig. 5B	Set of 17 unique tested backbones, AlphaFold2 models. Full complex	0.374	0.341 / 0.356 / 0.454 / 0.553	0.311	0.222 / 0.271 / 0.372 / 0.511	NA (see monomer)	NA (see monomer)
Symmetric Oligomer (C6) - Full oligomer	Ext. Data Fig. 5B	Set of 39 unique tested backbones, AlphaFold2 models. Full complex	0.578	0.464 / 0.512 / 0.611 / 0.669	0.417	0.219 / 0.318 / 0.537 / 0.799	NA (see monomer)	NA (see monomer)
Symmetric Oligomer (C6, > 750 amino acids) - Full oligomer	Ext. Data Fig. 5B	Set of 14 unique tested backbones, AlphaFold2 models. Full complex	0.439	0.335 / 0.355 / 0.501 / 0.558	0.315	0.214 / 0.262 / 0.409 / 0.689	NA (see monomer)	NA (see monomer)
Symmetric Oligomer (C8) - Full oligomer	Ext. Data Fig. 5B	Set of 35 unique tested backbones, AlphaFold2 models. Full complex	0.575	0.447 / 0.516 / 0.617 / 0.68	0.467	0.254 / 0.327 / 0.625 / 0.878	NA (see monomer)	NA (see monomer)
Symmetric Oligomer (C10) - Full oligomer	Ext. Data Fig. 5B	Set of 20 unique tested backbones, AlphaFold2 models. Full complex	0.597	0.477 / 0.556 / 0.643 / 0.661	0.73	0.480 / 0.624 / 0.826 / 0.900	NA (see monomer)	NA (see monomer)
Symmetric Oligomer (C12) - Full oligomer	Ext. Data Fig. 5B	Set of 9 unique tested backbones, AlphaFold2 models. Full complex	0.509	0.414 / 0.476 / 0.528 / 0.629	0.429	0.310 / 0.350 / 0.558 / 0.731	NA (see monomer)	NA (see monomer)
Symmetric Oligomer (D2) - Full oligomer	Ext. Data Fig. 5B	Set of 84 unique tested backbones, AlphaFold2 models. Full complex	0.5	0.416 / 0.464 / 0.536 / 0.596	0.34	0.263 / 0.306 / 0.388 / 0.515	NA (see monomer)	NA (see monomer)
Symmetric Oligomer (D3) - Full oligomer	Ext. Data Fig. 5B	Set of 26 unique tested backbones, AlphaFold2 models. Full complex	0.403	0.385 / 0.391 / 0.435 / 0.459	0.312	0.254 / 0.285 / 0.350 / 0.471	NA (see monomer)	NA (see monomer)
Symmetric Oligomer (D4) - Full oligomer	Ext. Data Fig. 5B	Set of 30 unique tested backbones, AlphaFold2 models. Full complex	0.384	0.343 / 0.359 / 0.403 / 0.423	0.293	0.232 / 0.267 / 0.341 / 0.439	NA (see monomer)	NA (see monomer)
Symmetric Oligomer (D5) - Full oligomer	Ext. Data Fig. 5B	Set of 7 unique tested backbones, AlphaFold2 models. Full complex	0.353	0.332 / 0.345 / 0.373 / 0.398	0.329	0.227 / 0.268 / 0.375 / 0.523	NA (see monomer)	NA (see monomer)
Sars-CoV-2 C3 binder scaffolds - Monomer	Fig. 5A	Set of 7 *in silico* successful backbones, AlphaFold2 models. Monomer extracted from oligomer	0.621	0.574 / 0.594 / 0.642 / 0.662	0.452	0.392 / 0.422 / 0.73 / 0.837	0.375	0.159/0.193/0.234/0.279/0.322
Sars-CoV-2 C3 binder scaffolds - Full oligomer	Fig. 5A	Set of 7 *in silico* successful backbones, AlphaFold2 models. Full complex	0.412	0.402 / 0.404 / 0.421 / 0.426	0.423	0.346 / 0.36 / 0.794 / 0.885	NA (see monomer)	NA (see monomer)
EC1 Enzyme Scaffolds	Fig. 4G	Set of 30 *in silico* successful EC1 active site scaffolds, AlphaFold2 models	0.663	0.58 / 0.615 / 0.687 / 0.702	0.458	0.332 / 0.403 / 0.524 / 0.614	0.29	0.183/0.193/0.193/0.227/0.244
EC2 Enzyme Scaffolds	Fig. 4G	Set of 5 *in silico* successful EC2 active site scaffolds, AlphaFold2 models	0.605	0.532 / 0.577 / 0.637 / 0.689	0.367	0.276 / 0.311 / 0.400 / 0.470	0.429	0.214/0.217/0.22/0.22/0.22
EC3 Enzyme Scaffolds	Fig. 4G	Set of 31 *in silico* successful EC3 active site scaffolds, AlphaFold2 models	0.578	0.522 / 0.55 / 0.603 / 0.631	0.353	0.285 / 0.320 / 0.400 / 0. 491	0.031	0.227/0.227/0.227/0.227/0.227
EC4 Enzyme Scaffolds	Fig. 4G	Set of 55 *in silico* successful EC4 active site scaffolds, AlphaFold2 models	0.639	0.533 / 0.607 / 0.673 / 0.704	0.442	0.328 / 0.389 / 0.494 / 0.572	0.304	0.184/0.2/0.213/0.227/0.26
EC5 Enzyme Scaffolds	Fig. 4G	Set of 30 *in silico* successful EC5 active site scaffolds, AlphaFold2 models	0.66	0.556 / 0.594 / 0.698 / 0.747	0.374	0.289 / 0.332 / 0.437 / 0.531	0.226	0.204/0.22/0.26/0.277/0.291
Unconditional length 100	Fig. 2C	Set of 100 designs used for benchmarking success rate at length 100, RFdiffusion outputs	0.701	0.615 / 0.662 / 0.76 / 0.863	0.394	0.302 / 0.353 / 0.445 / 0.543	0.16	0.258/0.28/0.3/0.32/0.37
Unconditional length 200	Fig. 2C	Set of 100 designs used for benchmarking success rate at length 200, RFdiffusion outputs	0.576	0.485 / 0.525 / 0.613 / 0.68	0.36	0.286 / 0.325 / 0.405 / 0.506	0.32	0.118/0.139/0.18/0.232/0.287
Unconditional length 300	Fig. 2C	Set of 100 designs used for benchmarking success rate at length 300, RFdiffusion outputs	0.526	0.462 / 0.490 / 0.585 / 0.644	0.368	0.279 / 0.329 / 0.418 / 0.531	0.39	0.113/0.14/0.163/0.182/0.251
Unconditional length 400	Fig. 2C	Set of 100 designs used for benchmarking success rate at length 400, RFdiffusion outputs	0.478	0.420 / 0.448 / 0.521 / 0.593	0.353	0.274 / 0.312 / 0.419 / 0.496	0.41	0.082/0.108/0.13/0.162/0.225
Unconditional length 600	Fig. 2C	Set of 100 designs used for benchmarking success rate at length 600, RFdiffusion outputs	0.413	0.377 / 0.396 / 0.439 / 0.486	0.35	0.278 / 0.314 / 0.39 / 0.499	0.39	0.048/0.074/0.092/0.107/0.192
Unconditional length 800	Fig. 2C	Set of 100 designs used for benchmarking success rate at length 800, RFdiffusion outputs	0.376	0.347 / 0.361 / 0.394 / 0.420	0.322	0.261 / 0.287 / 0.355 / 0.403	0.39	0.045/0.064/0.076/0.098/0.128
Unconditional length 1000	Fig. 2C	Set of 100 designs used for benchmarking success rate at length 1000, RFdiffusion outputs	0.353	0.327 / 0.339 / 0.366 / 0.393	0.296	0.256 / 0.278 / 0.315 / 0.355	0.31	0.032/0.041/0.047/0.067/0.09
TIM barrel	Ext. Data Fig. 4	Set of 12432 tim-barrel fold conditioned designs	NA	NA	NA	NA	0.19	0.11/0.148/0.177/0.212/0.263
NTF2	Ext. Data Fig. 4	Set of 7200 ntf2 fold conditioned designs	NA	NA	NA	NA	0.138	0.171/0.21/0.244/0.278/0.328

Supplementary Information Table 1 - Part 1/3

Dataset	Associated Figures	Description of analyzed dataset	(TMalign) Median of highest TM Score to PDB	(TMalign) 5th, 25th, 75th, 95th percentile highest TMscore to PDB	(TMalign) Median pairwise TM score within filtered designs	(TMalign) 5th, 25th, 75th, 95th percentile pairwise TM score	(BLAST) Fraction with significant blast hits (E-value < 0.1) in UniRef90	(BLAST) Query SeqID for best UniRef90 hit (5th/25th/50th/75th/95th percentiles)
rsv_site4 (noise 1)	Fig. 4A	Set of 31 *in silico* successful rsv_site4 designs with 1X noise scale, AlphaFold2 model, excluding "motif"	0.637	0.542 / 0.605 / 0.696 / 0.771	0.39	0.278 / 0.339 / 0.456 / 0.589	0.129	0.055/0.069/0.078/0.08/0.08
rsv_site4 (noise 0)	Fig. 4A	Set of 40 *in silico* successful rsv_site4 designs with 0x noise scale, AlphaFold2 model, excluding "motif"	0.654	0.548 / 0.573 / 0.714 / 0.758	0.375	0.265 / 0.326 / 0.432 / 0.556	0.175	0.04/0.042/0.049/0.065/0.091
2KL8 (noise 1)	Fig. 4A	Set of 96 *in silico* successful 2kl8 designs with 1x noise, AlphaFold2 model, excluding "motif"	0.816	0.789 / 0.801 / 0.825 / 0.842	0.7	0.561 / 0.627 / 0.823 / 0.942	1	0.035/0.051/0.063/0.076/0.089
2KL8 (noise 0)	Fig. 4A	Set of 88 *in silico* successful 2kl8 designs with 0x noise, AlphaFold2 model, excluding "motif"	0.821	0.803 / 0.815 / 0.828 / 0.844	0.845	0.600 / 0.671 / 0.923 / 0.976	1	0.038/0.063/0.076/0.089/0.089
5tpn (noise 1)	Fig. 4A	Set of 59 *in silico* successful 5tpn designs with 1x noise, AlphaFold2 model, excluding "motif"	0.676	0.601 / 0.647 / 0.708 / 0.739	0.4	0.280 / 0.341 / 0.49 / 0.634	0.93	0.015/0.06/0.075/0.094/0.125
5tpn (noise 0)	Fig. 4A	Set of 61 *in silico* successful 5tpn designs with 0x noise, AlphaFold2 model, excluding "motif"	0.67	0.612 / 0.646 / 0.695 / 0.731	0.391	0.281 / 0.337 / 0.47 / 0.622	0.967	0.015/0.058/0.077/0.097/0.169
1BCF (noise 1)	Fig. 4A	Set of 98 in silico successful 1bcf designs with 1x noise, AlphaFold2 model, excluding "motif"	0.814	0.746 / 0.771 / 0.84 / 0.863	0.809	0.733 / 0.78 / 0.838 / 0.879	0.49	0.079/0.097/0.114/0.182/0.218
1BCF (noise 0)	Fig. 4A	Set of 100 *in silico* successful 1bcf designs with 0x noise, AlphaFold2 model, excluding "motif"	0.82	0.765 / 0.792 / 0.84 / 0.868	0.824	0.743 / 0.793 / 0.853 / 0.892	0.54	0.084/0.108/0.156/0.195/0.251
6VW1 (noise 1)	Fig. 4A	Set of 66 *in silico* successful 6vw1 designs with 1x noise, AlphaFold2 model, excluding "motif"	0.736	0.656 / 0.707 / 0.758 / 0.798	0.576	0.484 / 0.534 / 0.627 / 0.755	1	0.013/0.026/0.038/0.055/0.073
6VW1 (noise 0	Fig. 4A	Set of 69 *in silico* successful 6vw1 designs with 0x noise, AlphaFold2 model, excluding "motif"	0.743	0.674 / 0.712 / 0.771 / 0.798	0.583	0.489 / 0.539 / 0.636 / 0.743	1	0.0/0.015/0.039/0.055/0.082
3IXT (noise 1)	Fig. 4A	Set of 16 *in silico* successful 3ixt designs with 1x noise, AlphaFold2 model, excluding "motif"	0.768	0.714 / 0.721 / 0.801 / 0.882	0.483	0.386 / 0.448 / 0.53 / 0.604	1	0.0/0.0/0.0/0.035/0.122
3IXT (noise 0)	Fig. 4A	Set of 35 *in silico* successful 3ixt designs with 0x noise, AlphaFold2 model, excluding "motif"	0.787	0.708 / 0.748 / 0.804 / 0.837	0.48	0.395 / 0.438 / 0.533 / 0.778	1	0.0/0.0/0.0/0.06/0.14
5TRV_long (noise 1)	Fig. 4A	Set of 30 *in silico* successful 5trv_long designs with 1x noise, AlphaFold2 model, excluding "motif"	0.591	0.535 / 0.572 / 0.636 / 0.714	0.337	0.277 / 0.306 / 0.386 / 0.485	1	0.0/0.002/0.009/0.058/0.095
5TRV_long (noise 0)	Fig. 4A	Set of 37 *in silico* successful 5trv_long designs with 0x noise	0.609	0.54 / 0.562 / 0.637 / 0.719	0.351	0.28 / 0.313 / 0.411 / 0.535	1	0.0/0.009/0.034/0.069/0.107
6EXZ_long (noise 1)	Fig. 4A	Set of 51 *in silico* successful 6exz_long designs with 1x noise, AlphaFold2 model, excluding "motif"	0.665	0.605 / 0.648 / 0.711 / 0.743	0.397	0.327 / 0.363 / 0.445 / 0.56	0.49	0.036/0.036/0.091/0.227/0.28
6EXZ_long (noise 0)	Fig. 4A	Set of 76 *in silico* successful 6exz_long designs with 0x noise	0.707	0.615 / 0.666 / 0.737 / 0.778	0.413	0.334 / 0.378 / 0.462 / 0.566	0.51	0.018/0.027/0.064/0.114/0.274
6E6R_med (noise 1)	Fig. 4A	Set of 67 *in silico* successful designs with 1x noise, AlphaFold2 model, excluding "motif"	0.75	0.661 / 0.728 / 0.784 / 0.83	0.433	0.354 / 0.396 / 0.478 / 0.577	0.164	0.173/0.237/0.269/0.282/0.321
6E6R_med (noise 0)	Fig. 4A	Set of 89 *in silico* successful designs with 0x noise, AlphaFold2 model, excluding "motif"	0.768	0.651 / 0.721 / 0.802 / 0.841	0.441	0.357 / 0.403 / 0.489 / 0.599	0.134	0.181/0.231/0.244/0.292/0.333
7MRX_128 (noise 1)	Fig. 4A	Set of 4 *in silico* successful designs with 1x noise, AlphaFold2 model, excluding "motif"	0.655	0.616 / 0.640 / 0.691 / 0.766	0.354	0.328 / 0.333 / 0.377 / 0.498	1	0.023/0.023/0.035/0.055/0.073
7MRX_128 (noise 0)	Fig. 4A	Set of 9 *in silico* successful designs with 0x noise, AlphaFold2 model, excluding "motif"	0.678	0.584 / 0.620 / 0.697 / 0.730	0.461	0.337 / 0.405 / 0.508 / 0.745	1	0.0/0.039/0.047/0.109/0.123
6E6R_long (noise 1)	Fig. 4A	Set of 63 *in silico* successful designs with 1x noise, AlphaFold2 model, excluding "motif"	0.7	0.618 / 0.654 / 0.728 / 0.811	0.398	0.321 / 0.364 / 0.441 / 0.515	0.206	0.148/0.204/0.25/0.269/0.302
6E6R_long (noise 0)	Fig. 4A	Set of 86 *in silico* successful designs with 0x noise, AlphaFold2 model, excluding "motif"	0.702	0.635 / 0.670 / 0.746 / 0.827	0.421	0.338 / 0.38 / 0.467 / 0.558	0.15	0.176/0.185/0.25/0.269/0.315
5TRV_short (noise 1)	Fig. 4A	Set of 7 *in silico* successful designs with 1x noise, AlphaFold2 model, excluding "motif"	0.737	0.69 / 0.719 / 0.754 / 0.818	0.582	0.344 / 0.352 / 0.813 / 0.844	1	0.0/0.0/0.0/0.018/0.043
5TRV_short (noise 0)	Fig. 4A	Set of 4 *in silico* successful designs with 0x noise, AlphaFold2 model, excluding "motif"	0.822	0.77 / 0.796 / 0.845 / 0.863	0.415	0.314 / 0.366 / 0.484 / 0.576	1	0.0/0.0/0.009/0.018/0.018
1PRW (noise 1)	Fig. 4A	Set of 9 *in silico* successful designs with 1x noise, AlphaFold2 model, excluding "motif"	0.616	0.588 / 0.610 / 0.634 / 0.668	0.56	0.353 / 0.486 / 0.66 / 0.739	1	0.061/0.086/0.091/0.112/0.136
1PRW (nosie 0)	Fig. 4A	Set of 8 *in silico* successful designs with 0x noise, AlphaFold2 model, excluding "motif"	0.575	0.535 / 0.563 / 0.622 / 0.687	0.452	0.302 / 0.386 / 0.542 / 0.642	1	0.055/0.061/0.082/0.088/0.099
6EXZ_med (noise 1)	Fig. 4A	Set of 33 *in silico* successful designs with 1x noise, AlphaFold2 model, excluding "motif"	0.728	0.656 / 0.698 / 0.752 / 0.776	0.433	0.354 / 0.394 / 0.484 / 0.578	0.82	0.012/0.025/0.038/0.081/0.134
6EXZ_med (noise 0)	Fig. 4A	Set of 49 *in silico* successful designs with 0x noise, AlphaFold2 model, excluding "motif"	0.736	0.687 / 0.716 / 0.756 / 0.779	0.456	0.369 / 0.415 / 0.526 / 0.699	0.82	0.025/0.05/0.062/0.1/0.154
6E6R_short (noise 1)	Fig. 4A	Set of 29 *in silico* successful designs with 1x noise, AlphaFold2 model, excluding "motif"	0.824	0.752 / 0.771 / 0.845 / 0.886	0.486	0.422 / 0.459 / 0.522 / 0.600	0.034	0.375/0.375/0.375/0.375/0.375
6E6R_short (noise 0)	Fig. 4A	Set of 39 *in silico* successful designs with 0x noise, AlphaFold2 model, excluding "motif"	0.824	0.733 / 0.794 / 0.848 / 0.882	0.486	0.413 / 0.454 / 0.546 / 0.661	0.1	0.215/0.24/0.292/0.344/0.369
5IUS (noise 1)	Fig. 4A	NA (no *in silico* successful 1x noise designs), AlphaFold2 model, excluding "motif"	NA	NA	NA	NA	NA	NA
5IUS (noise 0)	Fig. 4A	Set of 2 *in silico* successful designs with 0x noise, AlphaFold2 model, excluding "motif"	0.688	NA	0.465	NA	1	0.021/0.024/0.027/0.03/0.033
5TRV_med (noise 1)	Fig. 4A	Set of 20 *in silico* successful designs with 1x noise, AlphaFold2 model, excluding "motif"	0.643	0.587 / 0.614 / 0.672 / 0.701	0.342	0.289 / 0.321 / 0.381 / 0.483	1	0.0/0.0/0.012/0.07/0.084
5TRV_med (noise 0)	Fig. 4A	Set of 24 *in silico* successful designs with 0x noise, AlphaFold2 model, excluding "motif"	0.647	0.560 / 0.621 / 0.673 / 0.705	0.36	0.279 / 0.330 / 0.418 / 0.595	1	0.0/0.0/0.012/0.047/0.09
7MRX_85 (noise 1)	Fig. 4A	Set of 6 *in silico* successful designs with 1x noise, AlphaFold2 model, excluding "motif"	0.711	0.642 / 0.675 / 0.731 / 0.756	0.384	0.335 / 0.376 / 0.477 / 0.581	1	0.012/0.053/0.076/0.1/0.115
7MRX_85 (noise 0)	Fig. 4A	Set of 11 *in silico* successful designs with 0x noise, AlphaFold2 model, excluding "motif"	0.711	0.656 / 0.695 / 0.737 / 0.762	0.457	0.362 / 0.419 / 0.51 / 0.867	1	0.029/0.047/0.059/0.112/0.141
1YCR (noise 1)	Fig. 4A	Set of 58 *in silico* successful designs with 1x noise, AlphaFold2 model, excluding "motif"	0.749	0.669 / 0.718 / 0.782 / 0.886	0.423	0.338 / 0.382 / 0.482 / 0.610	0.14	0.207/0.232/0.266/0.331/0.396
1YCR (noise 0)	Fig. 4A	Set of 74 *in silico* successful designs with 0x noise, AlphaFold2 model, excluding "motif"	0.757	0.680 / 0.724 / 0.799 / 0.888	0.439	0.349 / 0.396 / 0.507 / 0.689	0.0676	0.206/0.232/0.25/0.354/0.426
6EXZ_short (noise 1)	Fig. 4A	Set of 4 *in silico* successful designs with 1x noise, AlphaFold2 model, excluding "motif"	0.746	0.696 / 0.722 / 0.779 / 0.828	0.455	0.433 / 0.439 / 0.514 / 0.550	0.75	0.042/0.05/0.06/0.07/0.078
6EXZ_short (noise 0)	Fig. 4A	Set of 2 *in silico* successful designs with 0x noise, AlphaFold2 model, excluding "motif"	0.736	0.704 / 0.718 / 0.754 / 0.768	0.438	NA	1	0.062/0.07/0.08/0.09/0.098
7MRX_60 (noise 1)	Fig. 4A	NA (no *in silico* successful 1x noise designs), AlphaFold2 model, excluding "motif"	NA	NA	NA	NA	NA	NA
7MRX_60 (noise 0)	Fig. 4A	Set of 2 *in silico* successful designs with 0x noise, AlphaFold2 model, excluding "motif"	0.792	NA	0.472	NA	1	0.051/0.054/0.058/0.062/0.066
5WN9 (noise 1)	Fig. 4A	Set of 1 *in silico* successful designs with 1x noise, AlphaFold2 model, excluding "motif"	0.636	NA	NA	NA	1	0/0/0/0/0 (alignments were always on motif)
5WN9 (noise 0)	Fig. 4A	NA (no *in silico* successful 0x noise designs), AlphaFold2 model, excluding "motif"	NA	NA	NA	NA	NA (no hits)	NA (no hits)
1QJG (noise 1)	Fig. 4A	Set of 2 *in silico* successful designs with 1x noise, AlphaFold2 model, excluding "motif"	0.717	NA	0.642	NA	NA (no hits)	NA (no hits)

Supplementary Information Table 1 - Part 2/3

Dataset	Associated Figures	Description of analyzed dataset	(TMalign) Median of highest TM Score to PDB	(TMalign) 5th, 25th, 75th, 95th percentile highest TMscore to PDB	(TMalign) Median pairwise TM score within filtered designs	(TMalign) 5th, 25th, 75th, 95th percentile pairwise TM score	(BLAST) Fraction with significant blast hits (E-value < 0.1) in UniRef90	(BLAST) Query SeqID for best UniRef90 hit (5th/25th/50th/75th/95th percentiles)
Orphan_single_motif_7A8S	Supp. Inf. Fig. 7	Set of 83 in silico successful designs with 1x noise, AlphaFold2 model, excluding "motif"	0.668	0.6 / 0.635 / 0.701 / 0.751	0.421	0.317 / 0.372 / 0.484 / 0.604	0.54	0.174/0.191/0.217/0.252/0.313
Orphan_single_motif_7AHO	Supp. Inf. Fig. 7	Set of 18 in silico successful designs with 1x noise, AlphaFold2 model, excluding "motif"	0.66	0.546 / 0.613 / 0.693 / 0.756	0.385	0.278 / 0.326 / 0.459 / 0.616	0.61	0.17/0.183/0.2/0.252/0.304
Orphan_single_motif_7DGW	Supp. Inf. Fig. 7	Set of 97 in silico successful designs with 1x noise, AlphaFold2 model, excluding "motif"	0.822	0.693 / 0.766 / 0.847 / 0.88	0.494	0.379 / 0.439 / 0.563 / 0.705	0.26	0.21/0.261/0.287/0.33/0.355
Orphan_single_motif_7DNS	Supp. Inf. Fig. 7	Set of 81 in silico successful designs with 1x noise, AlphaFold2 model, excluding "motif"	0.669	0.584 / 0.627 / 0.709 / 0.81	0.394	0.321 / 0.359 / 0.435 / 0.521	0.27	0.167/0.23/0.278/0.311/0.364
Orphan_single_motif_7F7P	Supp. Inf. Fig. 7	Set of 64 in silico successful designs with 1x noise, AlphaFold2 model, excluding "motif"	0.787	0.683 / 0.73 / 0.841 / 0.876	0.48	0.355 / 0.42 / 0.548 / 0.681	0.44	0.183/0.259/0.274/0.307/0.345
Orphan_single_motif_7K3H	Supp. Inf. Fig. 7	Set of 83 in silico successful designs with 1x noise, AlphaFold2 model, excluding "motif"	0.842	0.782 / 0.817 / 0.867 / 0.932	0.503	0.408 / 0.46 / 0.565 / 0.684	0.66	0.206/0.261/0.304/0.339/0.391
Orphan_single_motif_7KUW	Supp. Inf. Fig. 7	Set of 85 in silico successful designs with 1x noise, AlphaFold2 model, excluding "motif"	0.674	0.583 / 0.634 / 0.708 / 0.741	0.394	0.323 / 0.36 / 0.434 / 0.515	0.21	0.181/0.226/0.252/0.298/0.357
Orphan_single_motif_7KWW	Supp. Inf. Fig. 7	Set of 5 in silico successful designs with 1x noise, AlphaFold2 model, excluding "motif"	0.623	0.593 / 0.615 / 0.646 / 0.679	0.339	0.298 / 0.326 / 0.453 / 0.524	0.8	0.132/0.137/0.139/0.167/0.235
Orphan_single_motif_7MQQ	Supp. Inf. Fig. 7	Set of 42 in silico successful designs with 1x noise, AlphaFold2 model, excluding "motif"	0.749	0.629 / 0.688 / 0.792 / 0.869	0.431	0.339 / 0.387 / 0.486 / 0.609	0.33	0.208/0.237/0.287/0.296/0.313
Orphan_single_motif_7S5L	Supp. Inf. Fig. 7	Set of 58 in silico successful designs with 1x noise, AlphaFold2 model, excluding "motif"	0.756	0.623 / 0.703 / 0.809 / 0.855	0.449	0.335 / 0.39 / 0.513 / 0.682	0.48	0.226/0.25/0.291/0.313/0.357
Orphan_single_motif_7TJL	Supp. Inf. Fig. 7	Set of 67 in silico successful designs with 1x noise, AlphaFold2 model, excluding "motif"	0.813	0.713 / 0.771 / 0.85 / 0.931	0.478	0.381 / 0.432 / 0.533 / 0.647	0.25	0.197/0.252/0.287/0.322/0.35
Training_set_single_motif_1YES	Supp. Inf. Fig. 7	Set of 12 in silico successful designs with 1x noise, AlphaFold2 model, excluding "motif"	0.697	0.631 / 0.68 / 0.771 / 0.855	0.408	0.323 / 0.377 / 0.457 / 0.54	0.75	0.19/0.243/0.261/0.304/0.355
Training_set_single_motif_2EF5	Supp. Inf. Fig. 7	Set of 18 in silico successful designs with 1x noise, AlphaFold2 model, excluding "motif"	0.667	0.603 / 0.652 / 0.698 / 0.715	0.404	0.301 / 0.338 / 0.51 / 0.627	0.167	0.263/0.274/0.287/0.317/0.342
Training_set_single_motif_2FYD	Supp. Inf. Fig. 7	Set of 17 in silico successful designs with 1x noise, AlphaFold2 model, excluding "motif"	0.645	0.586 / 0.627 / 0.684 / 0.743	0.359	0.305 / 0.333 / 0.395 / 0.472	0.29	0.146/0.174/0.235/0.235/0.311
Training_set_single_motif_2W7Y	Supp. Inf. Fig. 7	Set of 1 in silico successful designs with 1x noise, AlphaFold2 model, excluding "motif"	0.605	0.605 / 0.605 / 0.605 / 0.605			NA (no hits)	NA (no hits)
Training_set_single_motif_3ES3	Supp. Inf. Fig. 7	Set of 85 in silico successful designs with 1x noise, AlphaFold2 model, excluding "motif"	0.824	0.703 / 0.785 / 0.852 / 0.888	0.492	0.373 / 0.437 / 0.557 / 0.681	0.46	0.174/0.235/0.27/0.313/0.376
Training_set_single_motif_3FKA	Supp. Inf. Fig. 7	Set of 54 in silico successful designs with 1x noise, AlphaFold2 model, excluding "motif"	0.625	0.572 / 0.593 / 0.658 / 0.702	0.385	0.291 / 0.336 / 0.442 / 0.539	0.333	0.155/0.191/0.248/0.285/0.301
Training_set_single_motif_3TQB	Supp. Inf. Fig. 7	Set of 4 in silico successful designs with 1x noise, AlphaFold2 model, excluding "motif"	0.6	0.572 / 0.591 / 0.603 / 0.605	0.364	0.249 / 0.275 / 0.416 / 0.497	0.5	0.203/0.217/0.235/0.252/0.266
Training_set_single_motif_4JWC	Supp. Inf. Fig. 7	Set of 44 in silico successful designs with 1x noise, AlphaFold2 model, excluding "motif"	0.821	0.658 / 0.75 / 0.866 / 0.901	0.489	0.369 / 0.426 / 0.575 / 0.75	0.386	0.2/0.252/0.278/0.313/0.325
Training_set_single_motif_4WSF	Supp. Inf. Fig. 7	Set of 24 in silico successful designs with 1x noise, AlphaFold2 model, excluding "motif"	0.649	0.578 / 0.633 / 0.675 / 0.693	0.402	0.307 / 0.355 / 0.467 / 0.554	0.333	0.166/0.215/0.252/0.289/0.367
Training_set_single_motif_4XJC	Supp. Inf. Fig. 7	Set of 11 in silico successful designs with 1x noise, AlphaFold2 model, excluding "motif"	0.633	0.589 / 0.612 / 0.647 / 0.684	0.379	0.303 / 0.336 / 0.432 / 0.491	0.72	0.148/0.198/0.23/0.276/0.301
Training_set_single_motif_5ECF	Supp. Inf. Fig. 7	Set of 74 in silico successful designs with 1x noise, AlphaFold2 model, excluding "motif"	0.851	0.792 / 0.82 / 0.874 / 0.926	0.517	0.42 / 0.471 / 0.592 / 0.716	0.41	0.2/0.23/0.283/0.346/0.375
Training_set_single_motig_5NE0	Supp. Inf. Fig. 7	Set of 1 in silico successful designs with 1x noise, AlphaFold2 model, excluding "motif"	0.648	0.648 / 0.648 / 0.648 / 0.648			1	0.217/0.217/0.217/0.217/0.217
Orphan_double_motif_7A8S	Supp. Inf. Fig. 7	Set of 2 in silico successful designs with 1x noise, AlphaFold2 model, excluding "motif"	0.603	0.577 / 0.589 / 0.618 / 0.63	0.385	NA	1	0.337/0.345/0.354/0.362/0.37
Orphan_double_motif_7CG5	Supp. Inf. Fig. 7	Set of 21 in silico successful designs with 1x noise, AlphaFold2 model, excluding "motif"	0.646	0.569 / 0.616 / 0.685 / 0.72	0.542	0.362 / 0.454 / 0.637 / 0.734	0.9	0.257/0.275/0.3/0.314/0.343
Orphan_double_motif_7DGW	Supp. Inf. Fig. 7	Set of 3 in silico successful designs with 1x noise, AlphaFold2 model, excluding "motif"	0.722	0.712 / 0.716 / 0.743 / 0.76	0.483	NA	0.333	0.35/0.35/0.35/0.35/0.35
Orphan_double_motif_7DNS	Supp. Inf. Fig. 7	Set of 52 in silico successful designs with 1x noise, AlphaFold2 model, excluding "motif"	0.635	0.58 / 0.603 / 0.666 / 0.718	0.391	0.309 / 0.352 / 0.461 / 0.614	0.94	0.271/0.286/0.293/0.314/0.343
Orphan_double_motif_7F7P	Supp. Inf. Fig. 7	Set of 1 in silico successful designs with 1x noise, AlphaFold2 model, excluding "motif"	0.609	0.609 / 0.609 / 0.609 / 0.609			1	0.2/0.2/0.2/0.2/0.2
Orphan_double_motif_7K3H	Supp. Inf. Fig. 7	Set of 84 in silico successful designs with 1x noise, AlphaFold2 model, excluding "motif"	0.765	0.597 / 0.711 / 0.788 / 0.831	0.435	0.335 / 0.381 / 0.519 / 0.792	0.37	0.2/0.261/0.286/0.314/0.35
Orphan_double_motif_7KUW	Supp. Inf. Fig. 7	Set of 26 in silico successful designs with 1x noise, AlphaFold2 model, excluding "motif"	0.643	0.566 / 0.609 / 0.665 / 0.702	0.413	0.318 / 0.36 / 0.482 / 0.671	0.58	0.189/0.221/0.25/0.268/0.29
Orphan_double_motif_7MQQ	Supp. Inf. Fig. 7	Set of 1 in silico successful designs with 1x noise, AlphaFold2 model, excluding "motif"	0.621	0.621 / 0.621 / 0.621 / 0.621			NA (no hits)	NA (no hits)
Orphan_double_motif_7S5L	Supp. Inf. Fig. 7	Set of 32 in silico successful designs with 1x noise, AlphaFold2 model, excluding "motif"	0.629	0.577 / 0.605 / 0.652 / 0.682	0.365	0.285 / 0.329 / 0.409 / 0.524	0.938	0.218/0.238/0.254/0.286/0.315
Orphan_double_motif_7TJL	Supp. Inf. Fig. 7	Set of 54 in silico successful designs with 1x noise, AlphaFold2 model, excluding "motif"	0.64	0.598 / 0.626 / 0.662 / 0.69	0.447	0.334 / 0.394 / 0.528 / 0.71	0.037	0.265/0.266/0.268/0.27/0.271
Training_set_double_motif_1YES	Supp. Inf. Fig. 7	Set of 3 in silico successful designs with 1x noise, AlphaFold2 model, excluding "motif"	0.627	0.559 / 0.589 / 0.637 / 0.645	0.572	NA	1	0.259/0.264/0.271/0.3/0.323
Training_set_double_motif_2FYD	Supp. Inf. Fig. 7	Set of 8 in silico successful designs with 1x noise, AlphaFold2 model, excluding "motif"	0.576	0.526 / 0.565 / 0.615 / 0.677	0.428	0.301 / 0.367 / 0.549 / 0.594	0.125	0.293/0.293/0.293/0.293/0.293
Training_set_double_motif_3ES3	Supp. Inf. Fig. 7	Set of 55 in silico successful designs with 1x noise, AlphaFold2 model, excluding "motif"	0.787	0.697 / 0.749 / 0.839 / 0.889	0.561	0.403 / 0.483 / 0.637 / 0.767	0.38	0.193/0.25/0.3/0.314/0.35
Training_set_double_motif_3FKA	Supp. Inf. Fig. 7	Set of 38 in silico successful designs with 1x noise, AlphaFold2 model, excluding "motif"	0.559	0.515 / 0.53 / 0.589 / 0.618	0.446	0.313 / 0.386 / 0.513 / 0.649	0.95	0.289/0.307/0.321/0.336/0.352
Training_set_double_motif_3TQB	Supp. Inf. Fig. 7	Set of 13 in silico successful designs with 1x noise, AlphaFold2 model, excluding "motif"	0.591	0.528 / 0.549 / 0.609 / 0.628	0.448	0.321 / 0.408 / 0.497 / 0.591	1	0.149/0.164/0.179/0.193/0.207
Training_set_double_motif_5ECF	Supp. Inf. Fig. 7	Set of 4 in silico successful designs with 1x noise, AlphaFold2 model, excluding "motif"	0.592	0.561 / 0.575 / 0.611 / 0.63	0.41	0.327 / 0.348 / 0.436 / 0.457	NA (no hits)	NA (no hits)
Training_set_double_motif_5JKB	Supp. Inf. Fig. 7	Set of 31 in silico successful designs with 1x noise, AlphaFold2 model, excluding "motif"	0.603	0.54 / 0.576 / 0.642 / 0.715	0.427	0.303 / 0.369 / 0.494 / 0.622	0.77	0.121/0.143/0.186/0.214/0.317
Training_set_double_motif_5NE0	Supp. Inf. Fig. 7	Set of 58 in silico successful designs with 1x noise, AlphaFold2 model, excluding "motif"	0.599	0.545 / 0.567 / 0.622 / 0.671	0.359	0.295 / 0.328 / 0.403 / 0.516	0.72	0.221/0.25/0.264/0.279/0.3
Training_set_double_motif_6FFW	Supp. Inf. Fig. 7	Set of 73 in silico successful designs with 1x noise, AlphaFold2 model, excluding "motif"	0.621	0.549 / 0.588 / 0.662 / 0.725	0.354	0.273 / 0.314 / 0.405 / 0.555	0.47	0.216/0.238/0.25/0.27/0.293

Supplementary Information Table 1 - Part 3/3

Supplementary Information Table 1: Table of aggregate TMalign and protein BLAST scores against native databases for various sets of designs from the paper.

Description of columns:
Dataset: Name of the dataset being analyzed with TMalign/BLAST.
Associated Figures: Which figure(s) does the set of designs appear in.
Description of analyzed dataset: Fuller description of designs in the set analyzed.
(TMalign) Median of highest TM score to PDB: The median value of the highest TMalign score against the entire PDB that every design in the set had.
(TMalign) 5th, 25th, 75th, 95th percentile highest TMscore to PDB: Other percentiles of highest TMalign score against the entire PDB besides the median (50th).
(TMalign) Median pairwise TM score within filtered designs: Median TMscore found between designs when running TMalign of the entire set against itself.
(TMalign) 5th, 25th, 75th, 95th percentile pairwise TM score: Other percentiles of the pairwise TM score within the set of designs.
(BLAST) Fraction with significant blast hits (E-value < 0.1) in UniRef90: When running protein BLAST against UniRef90, what fraction of the designs have hits with E-value < 0.1.
(BLAST) Query SeqID for best UniRef90 hit (5th/25th/50th/75th/95th percentiles): When BLAST finds a hit against UniRef90 for a design with E-value less than 0.1, what are the various percentiles of sequence identity to the query sequence (in non-motif regions) that the hit has?

Part II

Supplementary Methods

1 RoseTTAFold: updated architecture and training details

In this section we provide an overview of relevant details of the three-track architecture of RoseTTAFold (RF) and its training. This architecture includes significant modifications as compared to the original RF [18], which are not a contribution of this work. The model is the "fully connected" model described fully in [60]. We provide this section to assist in the understanding of the architecture of RFdiffusion. The architecture in Methods Figure 1 below. For the purposes of reproducibility of this work, we also provide the initial RF weights from which RFdiffusion is trained (https://github.com/RosettaCommons/RFdiffusion/).

1.1 Backbone structure representation

RF adopts a rigid-frame representation of the residues that comprise protein backbones. The structure of an L residue backbone is described as a collection of residue frames $x = [x_1, \ldots, x_L]$, where each $x_l = (r_l, z_l)$ describes the translation $z_l \in \mathbb{R}^3$ and rigid rotation r_l of the lth residue, but when it is clear from context, we sometimes drop the residue subscript. In particular, each z_l represents the coordinates of the lth C_α carbon, and each r_l is a 3×3 rotation matrix that maps an axis-aligned residue with idealized geometry (i.e. bond lengths and angle) and its C_α at the origin to the positions of these atoms relative to the C_α. For any backbone atom coordinates (z_{C_α}, z_C and z_N) for a given residue we may apply a Gram-Schmidt process to compute a 3×3 rotation matrix r with rows

$$
\begin{aligned}
&r_1 = (z_C - z_{C_\alpha})/\|z_{C_\alpha} - z_C\|_2, \\
&r_2 = \left((z_N - z_{C_\alpha}) - ((z_N - z_{C_\alpha}) \cdot r_1)r_1\right)/\|(z_N - z_{C_\alpha}) - ((z_N - z_{C_\alpha}) \cdot r_1)r_1\|, \text{ and} \\
&r_3 = r_1 \times r_2,
\end{aligned}
\tag{1}
$$

where $\cdot$ and $\times$ are the dot- and cross-products, respectively. 3D backbone coordinates can then

be reconstructed by multiplication of idealized coordinates (with $z^*_{C_\alpha}$ at the origin, $z^*_C - z^*_{C_\alpha}$ along the x-axis, and $z^*_N - z^*_{C_\alpha}$ in the xy-plane) by r as

$$[z_C, z_N, z_{C_\alpha}] = r[z^*_C, z^*_N, z^*_{C_\alpha}] + z\vec{1}_3,$$

where $\vec{1}_3 = [1, 1, 1]$. Accordingly, modeling the coordinates of a triplet of backbone atoms is equivalent to modeling the C_α coordinate z and the rotation matrix r.

1.2 RoseTTAFold architecture

The updated RF architecture is depicted in Methods Figure 1. RF includes several architectural improvements from the original RoseTTAFold network [18]: 1) the 3D structure track now extends throughout the entire network, with coordinates initialized from a template structure; 2) biaxial attention is still used to update 2D pair features, with the addition of an attention bias coming from geometric constraints between residues in the current 3D structure; 3) a similar biased axial attention is used to update the 1D track, where the 2D and 3D tracks are used to bias the attention in the 1D sequence updates; and 4) the incorporation of "recycling" [17] in training, in which the network is executed multiple times with updated input embeddings based on outputs from the previous cycle; the model is trained by back-propagating only through the final iteration. RF contains two major types of architecture blocks: main three-track blocks and the final structure refinement blocks. The 3-track blocks consist of layers of biased row and column attention over the 1D and 2D features, SE(3)-equivariant layers [61, 62] to update 3D coordinates, and layers to communicate between 1D, 2D, and 3D features. The structure refinement block is based on an SE(3)-equivariant network which gives refined 3D coordinates based on given 1D and 2D features. In recycle iterations, coordinates entering the 3D track are initialized from the predictions output in the previous pass through the network, and as a result of the SE(3)-equivariance of the architecture, the predictions in recycling passes update these initial coordinates with SE(3)-equivariance. Following

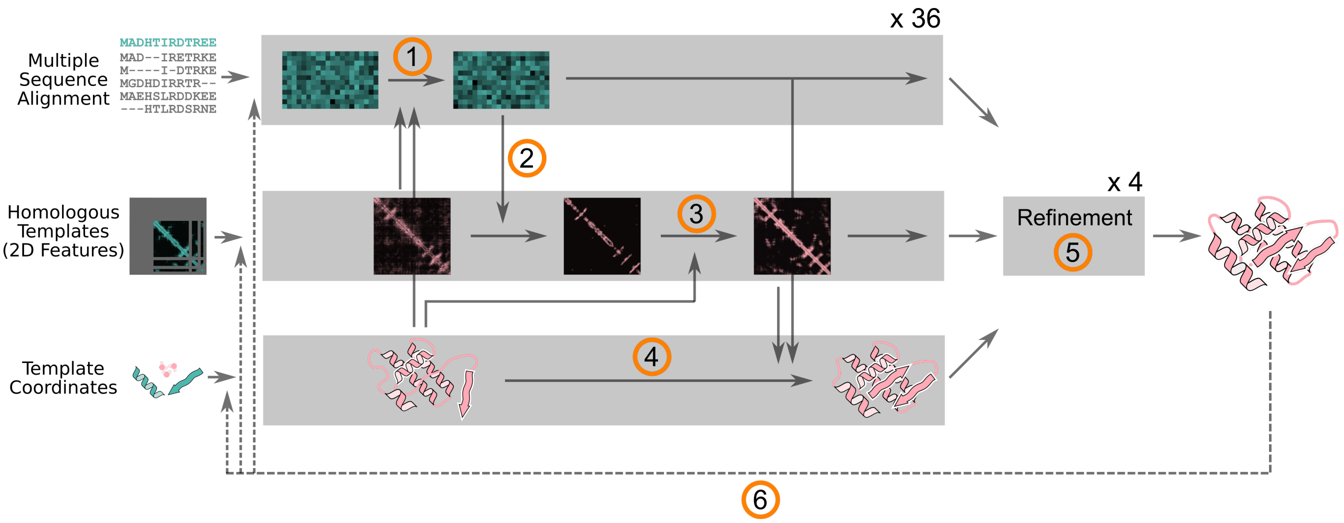

Supplementary Information Table 1: The three track architecture of RoseTTAFold.

AlphaFold2 [17], in addition to predicting backbone structure updates, at each layer (3-track and fine-tuning) RF predicts up to 4 sidechain torsion angles that define all protein sidechain atoms (and one backbone angle to place O). These key differences, denoted by corresponding numbers in Methods Figure 1, are described in detail below.

Inputs and outputs of RF: Before presenting the tensor input and outputs of RoseTTAFold, we introduce the relevant dimensions in these objects:

- L: The length of the query sequence
- I: The number of times the model will be executed (1 plus the number of recycles)
- T: The number of homologous structure provided to the model as templates
- N_short: The number of sequences in the truncated MSA
- N_long: The total number of sequences in the full MSA (capped at 1024)

Multimer prediction with multiple chains: As in the original RoseTTAFold [18] for hetero-complex structure prediction we indicate chain breaks in the positional encoding. Residue indices enter the network through the pair representation; for each pair of residues we include a sequence

Input name (Shape)	Description
`msa_masked`<br>(I, N_short, L, 48)	The truncated MSA with some portions of sequence masked (20aa, 1 unknown, 1 mask, MSA profiles (22), insertion/deletions (2), N-term/C-term (2))
`msa_full`<br>(I, N_long, L, 25)	The full length MSA (20aa, 1 unknown, 1 mask, insertion (1), N-term/C-term (2))
`seq`<br>(I, L, 22)	The sequence whose structure is being predicted (20aa, 1 unknown, 1 mask)
`xyz_prev`<br>(L, 27, 3)	The structure recycling information. In recycle steps, this contains the model's previous prediction. In the first iteration (before recycling), this feature is populated with template structure coordinates if available. Otherwise, this feature set to all zeros. N-C_α-C-O backbone (4), (up to) 10 sidechain atoms, (up to) 13 hydrogen atoms)
`idx_pdb`<br>(L)	The integer index of each residue. Used to assign each residue its neighboring residue.
`t1d`<br>(T, L, 22)	The one-dimensional features associated with each template structure. (20 amino acids, missing template token (1), template-match confidence (1))
`t2d`<br>(T, L, L, 44)	The two-dimensional features associated with each template structure. (36 distance bins (2-20Å, 0.5Å bins) + 1 final distance bin ($>$ 20Å), angle maps (sine and cosine of omega, theta and phi angle) (6), missing residue mask (1))
`xyz_t`<br>(T, L, 27, 3)	The structure of the template structures. This feature is immediately converted to a distogram and anglegram representation by the model. (N, Ca, C backbone atoms)
`alpha_t`<br>(T,L,10*3)	The backbone and sidechain torsion angles of each residue of the template structures. Initially T, L, 10, 2, with sine and cosine of (omega, phi, psi angles (3), (up to) 4 torsion angles, C_β bend (1), C_β twist (1), C_γ bend (1)). This is concatenated with a mask (T, L, 10, 1) indicating which torsion angles are present for a given amino acid, and reshaped to T, L, 30.
`msa_prev`<br>(N_short, L, Cm)	The MSA embedding recycling information. This is the model's previous embedding at each position in the truncated MSA. Cm = 256
`pair_prev`<br>(L, L, Cp)	The 2-D embedding recycling information. This is the model's previous embedding at each edge between each node. Cp = 128
`state_prev`<br>(L, Cs)	The 1-D embedding recycling information. This is the model's previous embedding at each position in the query sequence. Cs = 16

Supplementary Methods Table 1: Description of features input to RoseTTAFold.

**Output name** (shape)	**Description**
`msa` (N_short, L, Cm)	The model's final embedding at each position in the truncated MSA. Cm = 256
`pair` (L, L, Cp)	The model's final embedding at each edge between each node. Cp = 128
`state` (L, L, Cs)	The model's previous embedding at each position in the query sequence. Cs = 16
`xyz` (L, 27, 3)	The model's prediction of the structure. (N-Ca-C-O backbone (4), (up to) 10 sidechain atoms, (up to) 13 hydrogen atoms)
`alpha` (L, 10 * 3)	The model's prediction of sidechain torsions. Initially T, L, 10, 2, with sine and cosine of (omega, phi, psi angles (3), (up to) 4 torsion angles, C_β bend (1), C_β twist (1), C_γ bend (1)). This is concatenated with a mask (T, L, 10, 1) indicating which torsion angles are present for a given amino acid, and reshaped to T, L, 30.
`logits_aa` (N_short , L , 21)	The model's prediction of the unmasked, truncated MSA.
`pred_lddt` (L)	The model's prediction of the LDDT error of each residue.
`logits_dist` (Cdist,L,L)	The model's prediction of the binned distances $d_{l,l'}$ between residue pairs, where $d_{l,l'}$ is the Euclidean distance $C_{\beta,l}$ and $C_{\beta,l'}$. Cdist = 37.
`logits_omega` (Cdist,L,L)	The model's prediction of the binned ω angles between residue pairs, where $\omega_{l,l'}$ is the $C_{\alpha,l}$-$C_{\beta,l}$-$C_{\alpha,l'}$-$C_{\beta,l'}$ dihedral angle.
`logits_theta` (Cdist,L,L)	The model's prediction of the binned θ angles between residue pairs, where $\theta_{l,l'}$ is the N_l-$C_{\alpha,l}$-$C_{\beta,l}$-$C_{\beta,l'}$ dihedral angle.
`logits_phi` (Cphi,L,L)	The model's prediction of the binned ϕ angles between residue pairs, where $\phi_{l,l'}$ is the pseudo-bond angle dictating the direction of $C_{\beta,l'}$ from residue l's frame of reference. Cphi = 19.

Supplementary Methods Table 2: Description of outputs returned by RoseTTAFold.

distance feature clipped between -32 and 32 (with sign indicating direction). To indicate breaks between chains of subunits, we increment residue indices by 100 at the start of each chain.

1.2.1 MSA to MSA updates

The evolving MSA representation is updated with biases from the pair (2D) and structure (3D) tracks, before gated row-wise and column-wise self-attention analogous to the row-wise and column-wise attention in AlphaFold2 [17]. Specifically, pair features are concatenated with pairwise C_α-C_α distances from the emerging 3D structure to yield the bias. Node embeddings from the SE(3) transformer (State features) update the query sequence embedding. Sigmoid-gated row-wise self-attention with the bias term is performed, before unbiased sigmoid-gated column-wise self-attention. Skip connections bypass each attention block. A final feed-forward neural network yields the updated output.

1.2.2 MSA to Pair updates

As in RF, RF-NA, RF2 and AlphaFold2 [18, 55, 60, 17], evolving pair features are updated with co-evolution information extracted from the MSA representation. An outer-product on the MSA embedding captures the coevolutionary signal between all pairs of residues, and these are then aggregated across all sequences in the MSA. This aggregated outer product is then added to the pair features.

1.2.3 Pair to Pair updates

Pair features are updated with tied axial attention as implemented in the original RoseTTAFold [18], with a bias term emanating from the emerging 3D structure. Tied, sigmoid-gated row- and column-wise self-attention is performed, with projected C_α-C_α distances from the emerging 3D structure added to each block. A final feed-forward neural network yields the updated pair features.

1.2.4 Structure to Structure updates

RoseTTAFold uses the SE(3)-Transformer to refine the 3D coordinates [62]. Updates within the 3D track of RoseTTAFold incorporate the MSA and pair features. Protein backbone structure is represented by frames, as in AlphaFold2 [17]. These frames represent the SE(3) node features (C_α-position and N-C_α-C rotation). The protein graph is defined with these nodes connected to K-nearest neighbors. In each structure block, the node features are updated by the query sequence embedding. Pairwise features, encompassing the pair features, C_α-C_α distances from the emerging 3D structure and the primary sequence separation, define the edge inputs to the SE(3)-Transformer. The SE(3)-Transformer predicts the translation and rotation updates to the evolving structure. As in the original RoseTTAfold, degree 0 node features (called "State" features) are also output, which are used in the aforementioned MSA-to-MSA updates to calculate attention maps. Finally, as in RoseTTAFold-NA and RF2, sidechains are predicted using a sidechain-prediction network equivalent to that in AlphaFold2 [55, 60, 17].

1.2.5 Structure Refinement

The final four structure refinement layers are equivalent to the structure-structure updates described in Section 1.2.4.

1.2.6 Recycling

As in AlphaFold2 and RoseTTAFold-NA, recycling is now used to improve the structure prediction accuracy in RoseTTAFold. Specifically, pair features, MSA features and State features are recycled. Pairwise reconstructed C_β-C_β distances from the emerging 3D structure are concatenated with State-features and projected and added to the final pair-features from the previous recycle. This yields the recycled pair embedding, which is added to the initial pair features (deriving from template inputs). The final MSA and State embeddings from the previous recycle are similarly

added to their respective initial embedding at the subsequent recycle. During training, each example has 0-3 (randomly sampled) recycles with gradients tracked and back-propagated only in the final iteration, as in AlphaFold2 [17]

1.3 RoseTTAFold losses

$\mathcal{L}_{\mathbf{FAPE}}$**:** As in AlphaFold2, the primary structure loss used to train RF is the Frame-Aligned Point Error (FAPE) loss [17]. In 90% of training examples, $\mathcal{L}_{\text{FAPE}}$ is clamped at a maximum distance of 10Å, and left unclamped in the remaining 10% of examples. $\mathcal{L}_{\text{FAPE}}$ is split into two componens; one over just the backbone (frame) accuracy, and one over all atoms, as in AlphaFold2. These losses are applied equally on all 40 intermediate structures.

$\mathcal{L}_{\mathbf{tors}}$**:** An L2 loss is applied on the predicted torsion and chi angles (*alphas*), applied across all 40 intermediate structures.

$\mathcal{L}_{\mathbf{dist}}$**:** Losses are also applied on the pairwise-prediction in RoseTTAFold, which encompasses binned distance and orientation predictions (as in RoseTTAFold and TrRosetta [18, 63]). A cross-entropy loss is applied between this prediction and the pairwise-representation of the true structure.

$\mathcal{L}_{\mathbf{MLM}}$**:** Following the strategy described in AlphaFold2, 15% of input MSA tokens are masked (or corrupted) during training (of these, 70% are masked, 10% are mutated to a random residue, 10% are mutated to another residue in the MSA column, and 10% are non-mutated, but have a loss applied). RoseTTAFold predicts the identity of these residues, and a cross entropy is applied between the predicted logits and the true sequence.

$\mathcal{L}_{\mathbf{exp}}$**:** A binary cross entropy loss is also used to predict whether or not a residue is resolved in a structure, as described by AlphaFold2 [17].

$\mathcal{L}_{\textbf{accuracy}}$: A cross entropy loss is used to measure the difference between the true lDDT of the predicted structure and the predicted lDDT (per residue).

$\mathcal{L}_{\textbf{bond}}$: An L2 loss is applied between the true and predicted C-N bond lengths, as well as between the C_α-C-N and C-N-C_α bond angles of the predicted and true structures.

$\mathcal{L}_{\textbf{vdW}}$: Additionally, a loss penalizing clashes is applied, using an estimated Leonard Jones potential (akin to that used in Rosetta [58]), with attractive and repulsive components (scaled by 10^{12} and 10^6 respectively).

1.4 RoseTTAFold training

RF was trained on a mixture of datasets including 1) monomer/homo-oligomer structures in the PDB, 2) hetero-oligomer structures in the PDB (date cutoff August 2nd, 2021), 3) AlphaFold2 structural models having plDDT > 0.758, and 4) negative protein-protein interaction examples generated by random pairing. The training examples were sampled from each database with a ratio of 2:1:4:1. The model was trained using the masked language model ($\mathcal{L}_{\text{MLM}}$) loss, distogram prediction loss $\mathcal{L}_{\text{dist}}$, FAPE loss $\mathcal{L}_{\text{FAPE}}$, torsion angle loss $\mathcal{L}_{\text{tors}}$, accuracy estimation loss $\mathcal{L}_{\text{accuracy}}$, experimentally-resolved loss $\mathcal{L}_{\text{exp}}$, bond geometry loss $\mathcal{L}_{\text{bond}}$ and van der Waals (vdW) energy loss $\mathcal{L}_{\text{vdW}}$. For the initial round of training, only the first six loss terms were used with crop size 256. After 200 epochs of initial round training, the model was fine-tuned for a further 100 epochs, with all the loss terms and a crop size 384. RoseTTAFold was trained for 4 weeks on 64 V100 GPUs on Microsoft Azure. The training details are summarized in Supplementary Methods Table 3.

	Initial training	Fine-tuning
Crop size	256	384
Batch size	64	64
Loss function	3.0*$\mathcal{L}_{\text{MLM}}$ + 1.0*$\mathcal{L}_{\text{dist}}$ + 10.0*$\mathcal{L}_{\text{FAPE}}$ + 10.0*$\mathcal{L}_{\text{tors}}$+ 0.1*$\mathcal{L}_{\text{accuracy}}$ + 0.1*$\mathcal{L}_{\text{exp}}$	3.0*$\mathcal{L}_{\text{MLM}}$ + 1.0*$\mathcal{L}_{\text{dist}}$ + 10.0*$\mathcal{L}_{\text{FAPE}}$ + 10.0*$\mathcal{L}_{\text{tors}}$ + 0.1*$\mathcal{L}_{\text{accuracy}}$ + 0.1*$\mathcal{L}_{\text{bond}}$ + 0.1*$\mathcal{L}_{\text{vdW}}$ + 0.1*$\mathcal{L}_{\text{exp}}$
Learning rate, & scheduling	0.001, Linear warm-up for first 1000 optimization steps, then decay learning rate by 0.95 after every 15000 optimization steps	0.0005, No warm-up. Decay learning rate by 0.95 after every 15000 optimization steps
Examples per epoch	25600	25600
Number of epochs	200	100

Supplementary Methods Table 3: RoseTTAFold training hyperparameters.

2 RFdiffusion: principles and formulation as a generative model of structure

This section details how we have repurposed RoseTTAFold (RF) as the neural network in a diffusion model of protein backbones. Section 2.1 reviews denoising diffusion probabilistic models (DDPMs) to establish notation and terminology. RFdiffusion adapts DDPMs to the rigid-frame representation of residues used by RF (as described in Section 1.1), and Sections 2.2 and 2.3 describe the forward and reverse processes for translation and rotation components of this representation. Section 2.4 describes our use of *self-conditioning*. Section 2.5 presents the mean-squared-error denoising loss used in training, and discusses how minimizing this objective relates to learning the reverse process. Finally, Section 2.6 discusses geometric invariance in RFdiffusion. Some of the theoretical aspects underlying the treatment of the diffusion on residue orientations and geometric invariance are developed in greater detail in concurrent work [64]; these aspects are referred to throughout.

2.1 Diffusion probabilistic modeling background and notation

DDPMs [10, 11] are a class of generative models that approximate a distribution by parameterizing the reversal of a discrete-time diffusion process. The "forward" diffusion process starts with a sample $x^{(0)} \sim q(x^{(0)})$ from an unknown data distribution q, to which we have access only though samples. The data are corrupted at each of T steps, to obtain a sequence of increasingly noisy samples; for each $t = 1, \ldots, T$, we sample $x^{(t)} \sim q(x^{(t)} \mid x^{(t-1)})$ such that the final step $x^{(T)} \sim q(x^{(T)})$ is indistinguishable from a reference distribution that has no dependence on the data. DDPMs approximate $q(x^{(0)})$ with a second distribution $p(x^{(0)})$ parameterized by a backward transition kernel $p(x^{(t-1)} \mid x^{(t)})$ at each t. We train a neural network parameterizing each $p(x^{(t-1)} \mid x^{(t)})$ to approximate $q(x^{(t-1)}|x^{(t)})$. One then draws from $p(x^{(0)})$ by first sampling from the reference distribution $x^{(T)} \sim p(x^{(T)}) \approx q(x^{(T)})$, and then for each $t < T$ repeatedly denoising by sampling $x^{(t-1)} \sim p(x^{(t-1)} \mid x^{(t)})$ until $x^{(0)} \sim p(x^{(0)})$ is obtained. In the limit that the approximations $p(x^{(t-1)}|x^{(t)})$ and $p(x^{(t)})$ of $q(x^{(t-1)}|x^{(t)})$ and $q(x^{(T)})$ are exact, $p(x^{(0)}) = q(x^{(0)})$.

In our case, $q(x^{(0)})$ is a distribution over a native protein backbones parameterized by residue frames. We define the forward noising process independently over the rotational and translational components of this representation. We similarly model the reverse process transitions as conditionally independent across these components given $x^{(t)}$ as

$$p(x^{(t-1)} \mid x^{(t)}) = p(r^{(t-1)}|x^{(t)})p(z^{(t-1)}|x^{(t)}).$$

We next describe the details of the forward and reverse process conditionals for the translations in Section 2.2 and for the rotations in Section 2.3. We finally note that our treatment here may be viewed as a discretization of an SE(3)-equviariant, continuous-time diffusion process on the manifold $\mathrm{SE}(3)^L$, with Brownian motion defined through a product metric that separates across the rotations and translations associated with each of the L residues in a backbone. The full details

of continuous time view are beyond the scope of the present paper, and are developed in greater depth by Yim et al. [64].

2.2 Residue translations, forward and reverse transitions

Our forward process for translations follows closely from previous work by Trippe et al. [5] on C_α backbone generation, that treats C_α backbone coordinates as a 3D point cloud and corrupts them with 3D Gaussian noise. We let $\beta^{(1)}, \beta^{(2)}, \ldots, \beta^{(T)}$ be scalars between 0 and 1 that define a variance schedule such that for each $t = 1, 2, \ldots, T$ the transition density of the forward process is $q(z^{(t)} \mid z^{(t-1)}) = \mathcal{N}(z^{(t)}; \sqrt{1-\beta^{(t)}}z^{(t-1)}, \beta^{(t)} I_3)$. To sample $z^{(t)}$ during training, rather than ancestral sampling $z^{(s)}|z^{(s-1)}$ from $s = 1$ all the way up to $s = t$, we draw $z^{(t)}$ directly from the marginal distribution, $q(z^{(t)}|z^{(0)}) = \mathcal{N}\left(z^{(t)}; \sqrt{\bar{\alpha}^{(t)}}z^{(0)}, (1-\bar{\alpha}^{(t)})I_3\right)$, where we define $\bar{\alpha}^{(t)} = \prod_{s=1}^{t} \alpha^{(t)}$ with $\alpha^{(t)} = 1 - \beta^{(t)}$.

For the reverse process, we desire to use a prediction of denoised coordinates from RoseTTAFold. Given that $q(z^{(t-1)}|z^{(t)}, z^{(0)}) = \mathcal{N}(z^{(t-1)}; \tilde{\mu}(z^{(t)}, z^{(0)}), \tilde{\beta}^{(t)} I_3)$ for $\tilde{\mu}(z^{(t)}, z^{(0)}) = \frac{\sqrt{\bar{\alpha}^{(t-1)}}\beta^{(t)}}{1-\bar{\alpha}^{(t)}} z^{(0)} + \frac{\sqrt{\alpha^{(t)}}(1-\bar{\alpha}^{(t)})}{1-\bar{\alpha}^{(t)}} z^{(t)}$, and $\tilde{\beta}^{(t)} = \frac{1-\bar{\alpha}^{(t-1)}}{1-\bar{\alpha}^{(t)}}\beta^{(t)} \approx \beta^{(t)}$, we define the reverse transitions by

$$
\begin{aligned}
p(z^{(t-1)} \mid x^{(t)}) &= \mathcal{N}(z^{(t)}; \hat{\mu}(x^{(t)}), \beta^{(t)} I_3), \\
\text{with } \ \hat{\mu}(x^{(t)}) &= \frac{\sqrt{\bar{\alpha}^{(t-1)}}\beta^{(t)}}{1-\bar{\alpha}^{(t)}} \hat{z}^{(0)}(x^{(t)}) + \frac{\sqrt{\alpha^{(t)}}(1-\bar{\alpha}^{(t-1)})}{1-\bar{\alpha}^{(t)}} z^{(t)},
\end{aligned} \tag{2}
$$

where $\hat{z}^{(0)}(x^{(t)})$ denotes the predicted C_α coordinates obtained from RFdiffusion, $\hat{x}^{(0)}(x^{(t)})$.

Variance schedule and inference with fewer steps. In baseline inference with $T = 200$ steps, we use a linear variance schedule [11] wherein we choose $\beta^{(t)} = \beta^z_{min} + (\frac{t}{T})(\beta^z_{max} - \beta^z_{min})$ with $\beta^z_{min} = 0.01$ and $\beta^z_{max} = 0.07$. We chose these parameters such that the signal remaining in $z^{(t)}$ from $z^{(0)}$ (as quantified by $\bar{\alpha}^{(t)}$) decays slowly toward zero as t approaches T.

When using RFdiffusion at inference with a different number of timesteps $T' \neq T$, we modify

our variance schedule accordingly by adjusting the limits of the linear schedule as

$$\beta^{z\prime}_{min} = \frac{T}{T'}\beta^{z}_{min} \text{ and } \beta^{z\prime}_{max} = \frac{T}{T'}\beta^{z}_{max}. \tag{3}$$

This choice scales up the variance of the noise added in each step proportionally to the implied fraction of the trajectory traversed in each step.

2.3 Residue rotations, forward and reverse transitions

For the forward and reverse transitions on rotations, we adapt a generalization developed by De Bortoli et al. [23] of diffusion models to Riemannian manifolds. In particular, the space of 3×3 rotation matrices (known as the special orthogonal group of dimension 3, or $SO(3)$) is a compact Riemannian manifold where the techniques of Ho et al. [11] do not apply readily. In brief, De Bortoli et al. [23] build on the continuous-time score-based generative modeling framework of Song et al. [65] and define the forward process as Langevin dynamics on the manifold — and in particular as a Brownian motion when the manifold is compact. The time-reversal of this process is then characterized through the Stein score of the noised data distribution at each t. This subsection relies of some knowledge of properties of $SO(3)$ and its Lie algebra; we refer the reader to Sola et al. [66] for this background.

Forward process defined by Brownian motion on $SO(3)$: The form of the Brownian motion on a manifold is well-defined only with the choice of an inner-product on the associated tangent spaces $\mathcal{T}_r$; we choose a scaling of the Frobenius inner product as an inner product on the tangent spaces of $SO(3)$, such that for any $r \in SO(3)$ and A and B in a $\mathcal{T}_r$,

$$\langle A, B \rangle_{SO(3)} = \text{Trace}(A^\top B)/2. \tag{4}$$

The marginal distribution of a rotation matrix $r^{(t)}$ evolving according to Brownian motion for time t from an initial rotation $r^{(0)}$ is given by the $\mathcal{IG}_{SO(3)}$ distribution [67, 24], which we write as $r^{(t)} \sim \mathcal{IG}_{SO(3)}(\mu = r^{(0)}, \sigma^2 = t)$. With the choice of inner product in Equation (4), the density of the $\mathcal{IG}_{SO(3)}$ distribution with respect to the uniform distribution on $SO(3)$ is given by

$$\mathcal{IG}_{SO(3)}(r^{(t)}; \mu, \sigma^2) = f(\omega(\mu^\top r^{(t)}); \sigma^2), \text{ for } f(\omega; \sigma^2) = \sum_{l=0}^{\infty} (2l+1) e^{-l(l+1)\sigma^2/2} \frac{\sin((l+\frac{1}{2})\omega)}{\sin(\omega/2)}, \quad (5)$$

where μ is 3×3 mean rotation matrix and $\omega(r)$ denotes the angle of rotation in radians associated with a rotation r. The angle may be computed as $\omega(r) = \arccos\left[(\mathrm{trace}(R) - 1)/2\right]$. We refer the reader to [64, Proposition 3.3] for a verification of Equation (5). We approximate the power series in Equation (5) by its truncation after 2000 terms. We formulate a discrete-time forward noising by discretizing the Brownian motion, which provides: $q(r^{(t)} | r^{(t-1)}) = \mathcal{IG}_{SO(3)}(r^{(t)}; r^{(t-1)}, \sigma_t^2 - \sigma_{t-1}^2)$ and marginally $q(r^{(t)} \mid r^{(0)}) = \mathcal{IG}_{SO(3)}(r^{(t)}; r^{(0)}, \sigma_t^2)$, where $\sigma_1^2, \sigma_2^2, \ldots, \sigma_T^2$ is a variance schedule. We choose this schedule so that the rotations are corrupted at a rate similar to the forward process on translations (Supplementary Methods Table 6). In contrast to the translations, which converge to a Gaussian distribution as t increases, the rotations converge to the uniform distribution on $SO(3)$; this uniform distribution, also known as the Haar measure, is invariant to rotation.

Backward transition kernel: To approximate the reverse transitions for the rotations we take inspiration from De Bortoli et al. [23, Theorem 1] and approximate the discretized reversal by a geodesic random walk. In particular, reverse step updates for rotations are computed by taking a noisy step in the tangent space of $SO(3)$ in the direction of the gradient of the log density of a noised structure $x^{(t)}$ with respect to each rotation, and projecting back to the $SO(3)$ manifold using the exponential map De Bortoli et al. [23, Algorithm 1]. The size of the step and the variance of the noise added depend on the noising schedule as in Song et al. [65], and additionally depend on a choice of orthonormal basis for the tangent space; with the choice of inner-product in Equation (4),

the tangent space at I_3, $\mathcal{T}_{I_3}$ (known as the Lie algebra of $SO(3)$) has orthonormal basis vectors

$$f_1 = \begin{bmatrix} 0 & -1 & 0 \\ 1 & 0 & 0 \\ 0 & 0 & 0 \end{bmatrix}, \quad f_2 = \begin{bmatrix} 0 & 0 & 1 \\ 0 & 0 & 0 \\ -1 & 0 & 0 \end{bmatrix}, \quad \text{and} \quad f_3 = \begin{bmatrix} 0 & 0 & 0 \\ 0 & 0 & -1 \\ 0 & 1 & 0 \end{bmatrix}, \tag{6}$$

and for every other $r \in SO(3)$, $\mathcal{T}_r$ has orthonormal basis $\{rf_1, rf_2, rf_3\}$. Each step of the geodesic random walk is computed as

$$r^{(t-1)} = \exp_{r^{(t)}} \left\{ (\sigma_t^2 - \sigma_{t-1}^2) \nabla_{r^{(t)}} \log q(x^{(t)}) + \sqrt{\sigma_t^2 - \sigma_{t-1}^2} \sum_{d=1}^{3} \epsilon_d r^{(t)} f_d \right\}, \tag{7}$$

where $\nabla_{r^{(t)}} \log q(x^{(t)})$ in $\mathcal{T}_{r^{(t)}}$ denotes the Stein score of the forward process at time t, and $\exp_{r^{(t)}}$ denotes the exponential map from $\mathcal{T}_{r^{(t)}}$ to $SO(3)$, and $\epsilon_1, \epsilon_2, \epsilon_3 \overset{iid}{\sim} \mathcal{N}(0, 1)$. The exponential map $\exp_{r^{(t)}}$ may be computed as $\exp_{r^{(t)}}\{v\} = r^{(t)} \exp_{I_3}\{{r^{(t)}}^\top v\}$, where $\exp_{I_3}\{\cdot\}$ is the matrix exponential.

The variance schedule for the rotations is chosen by setting $\sigma_t = \sigma_{min} + \frac{t}{T}\beta_{min} + \frac{1}{2}(\frac{t}{T})^2(\beta^r_{max} - \beta^r_{min})$, with $\sigma_{min} = 0.02$, $\beta^r_{min} = 1.06$, and $\beta^r_{max} = 1.77$

Approximating the score with a denoising prediction: Equation (7) describes how one could sample from the reverse process using the score of the forward process. One could in principle learn this score function directly by score matching training [23]. However, we instead rely on an approximation that directly leverages RoseTTAFold's ability to predict denoised structures once

suitably trained. For a given t and $r^{(t)}$ we may write

$$\begin{aligned}\nabla_{r^{(t)}} \log q(x^{(t)}) &= \mathbb{E}_q\left[\nabla_{r^{(t)}} \log q(x^{(t)} \mid x^{(0)}) \mid x^{(t)}\right] \\ &= \mathbb{E}_q\left[\nabla_{r^{(t)}} \log q(r^{(t)} \mid r^{(0)}) \mid x^{(t)}\right] \\ &\approx \nabla_{r^{(t)}} \log q(r^{(t)} \mid r^{(0)} = \hat{r}^{(0)}) \\ &= \nabla_{r^{(t)}} \log \mathcal{IG}_{SO(3)}(r^{(t)}; \hat{r}^{(0)}, \sigma_t^2),\end{aligned} \tag{8}$$

where the first line is known as the denoising score matching identity [68], the second line is obtained from the conditional independence structure of the forward process, the third line is an approximation that can be thought of as replacing $q(r^{(0)} \mid r^{(t)})$ with a point mass on the noiseless rotation $\hat{r}^{(0)}$ predicted by RFdiffusion, and the final line recognizes the approximation as the gradient of the tractable $\mathcal{IG}_{SO(3)}$ log density. In the expressions above, we use the notation $\mathbb{E}_q[g(x^{(0)}, x^{(t)}) \mid x^{(t)}] = \int g(x^{(0)}, x^{(t)}) q(x^{(0)} \mid x^{(t)}) dx^{(0)}$ to describe the conditional expectation according to q of $g(x^{(0)}, x^{(t)})$ given $x^{(t)}$. We compute the score approximation in the final line of Equation (8) by applying the chain rule to obtain

$$\begin{aligned}\nabla_r \log \mathcal{IG}_{SO(3)}(r; \hat{r}, \sigma_t^2) &= \nabla_r \omega(\hat{r}^\top r) \frac{d}{d\omega} \log f(\omega; \sigma_t^2) \mid_{\omega=\omega(\hat{r}^\top r)} \\ &= r \frac{\log(\hat{r}^\top r)}{\omega(\hat{r}^\top r)} \frac{d}{d\omega} \log f(\omega, \sigma_t^2)|_{\omega=\omega(\hat{r}^\top r)}\end{aligned} \tag{9}$$

where $\log(\hat{r}^\top r)$ is the matrix logarithm and $\omega(r\hat{r}^\top)$ and f are as defined in Equation (5) [64, Proposition 3.4]. $r\frac{\log(\hat{r}^\top r)}{\omega(\hat{r}^\top r)}$ is a unit length perturbation in the direction of $r \log(\hat{r}^\top r)$, and $\frac{d}{d\omega} \log f(\omega, \sigma_t^2) \mid_{\omega=\omega(\hat{r}^\top r)}$ is a scaling of this direction.

We reasoned that the approximation in Equation (8) may be reasonably accurate for two reasons. First, in the case of diffusion probabilistic models with Gaussian noise, where optimizing to convergence would provide $\hat{z}^{(0)}(x^{(t)}) = \mathbb{E}_q\left[z^{(0)} \mid x^{(t)}\right]$, this approximation holds exactly in the sense that $\mathbb{E}_q[\nabla_{z^{(t)}} \log q(z^{(t)} \mid z^{(0)}) \mid x^{(t)}] = \nabla_{z^{(t)}} \log q(z^{(t)} \mid z^{(0)} = \hat{z})$ for $\hat{z} = \hat{z}^{(0)}(x^{(t)})$ (see Proposition

Algorithm 1 RFdiffusion rotation score approximation

function F$(\omega, \sigma^2, L = 2000)$ ▷ $\mathcal{IG}_{SO(3)}$ density factor, truncated to L terms

return $\sum_{l=0}^{L}(2l+1)e^{-l(l+1)\sigma^2/2}\frac{\sin((l+\frac{1}{2})\omega)}{\sin(\omega/2)}$

end function

function ROTATIONSCOREAPPROXIMATION$(r_t, \hat{r}_0, \sigma_t^2)$

$\vec{r}_{0t} = \log(\hat{r}_0^\top r_t)$ ▷ $\vec{r}_{0t} \in \mathbb{R}^{3,3}, \vec{r}_{0t} = -\vec{r}_{0t}^\top$

$\omega_{0t} = \arccos[(\text{trace}(\hat{r}_0^\top r_t) - 1)/2]$ ▷ angle of rotation $\omega_{0t} \in [0, \pi]$

▷ Compute score approximation

$s = \frac{r_t \vec{r}_{0t}}{\omega_{0t}} \cdot \frac{d}{d\omega} \log\text{F}(\omega; \sigma_t^2)|_{\omega=\omega_{0t}}$ ▷ $s \in \mathbb{R}^{3,3}$

return s

end function

1 below). Though this does not hold with equality with $\mathcal{IG}_{SO(3)}$, because $SO(3)$ is a Riemannian manifold and is therefore locally Euclidean, $\mathcal{IG}_{SO(3)}$ closely resembles a Gaussian for low t. Second, again when t is low, $x^{(t)}$ will be close to a plausible structure and, if the model is trained well, $q(r^{(0)} \mid x^{(t)})$ will be concentrated near $r^{(0)}$. Although approximation error may be non-trivial for larger t, we find this approximation to be empirically useful nonetheless. We present computation of this approximation to the score in Algorithm 1.

Altogether, the above derivation suggests updates for the rotations in the reverse process as

$$r^{(t-1)} = r^{(t)} \exp_{I_3}\left\{(\sigma_t^2 - \sigma_{t-1}^2) r^{(t)\top} \nabla_{r^{(t)}} \log \mathcal{IG}_{SO(3)}(r^{(t)}; \hat{r}^{(0)}, \sigma_t^2) + \sqrt{\sigma_t^2 - \sigma_{t-1}^2} \sum_{d=1}^{3} \epsilon_d f_d\right\}, \quad (10)$$

with $\nabla_{r^{(t)}} \mathcal{IG}_{SO(3)}(r^{(t)}; \hat{r}^{(0)}, \sigma_t^2)$ computed as in Equation (9).

Proposition 1. *Suppose $z^{(0)}, z^{(t)} \sim q(z^{(0)}, z^{(t)})$, and that $z^{(t)} \mid z^{(0)} \sim \mathcal{N}(z^{(t)}; \alpha z^{(0)}, \sigma^2)$ according to q for some α and σ^2. If $\hat{z}^{(0)}(z^{(t)}) = \mathbb{E}_q[z^{(0)} \mid z^{(t)}]$ for every $z^{(t)}$, then*

$$\mathbb{E}_q[\nabla_{z^{(t)}} \log q(z^{(t)} \mid z^{(0)}) \mid z^{(t)}] = \nabla_{z^{(t)}} \log q(z^{(t)} \mid z^{(0)} = \hat{z}), \quad \text{for } \hat{z} = \hat{z}^{(0)}(z^{(t)}). \quad (11)$$

Proof. To prove the result, we re-write the left hand side of Equation (11) to obtain

$$\begin{aligned}
\mathbb{E}_q[\nabla_{z^{(t)}} \log q(z^{(t)} \mid z^{(0)}) | z^{(t)}] &= \mathbb{E}_q[-(z^{(t)} - \alpha z^{(0)})/\sigma^2 \mid z^{(t)}] \\
&= -(z^{(t)} - \alpha \mathbb{E}_q[z^{(0)} \mid z^{(t)}])/\sigma^2 \\
&= -(z^{(t)} - \alpha \hat{z}^{(0)}(z^{(t)}))/\sigma^2 \\
&= \nabla_{z^{(t)}} \log q(z^{(t)} \mid z^{(0)} = \hat{z}), \text{ for } \hat{z} = \hat{z}^{(0)}(z^{(t)}),
\end{aligned} \tag{12}$$

as desired. □

2.4 Self-conditioning in reverse process sampling

Self-conditioning was introduced by Chen et al. [25], who showed the technique dramatically improves diffusion performance on image generation and image captioning tasks. We implement self-conditioning in the manner similar to how it is described in Chen et al. [25].

For sampling in diffusion generative models without self-conditioning, at each denoising step once $x^{(t)}$ has been sampled, the prediction of the denoised data from the previous step ($\hat{x}^{(0)}_{\text{prev.}} = \hat{x}^{(0)}(x^{(t+1)})$) is discarded. However, since each denoising step is typically small, successive $\hat{x}^{(0)}(x^{(t)})$ predictions can be similar, so much of the denoising computation must be repeated. By contrast, with self-conditioning one saves the denoising predictions at each step and provides them as an input to the denoising model at the next iteration, instead predicting $x^{(0)}$ as $\hat{x}^{(0)}(x^{(t)}, \hat{x}^{(0)}_{\text{prev.}})$. This process of providing previous network outputs as an input to subsequent iterations is reminiscent of "recycling" in AlphaFold [17] and our updated variant of RoseTTAFold (Section 1.2). When training with self-conditioning, on 50% of examples one performs a usual denoising step, setting $\hat{x}^{(0)}_{\text{prev.}} = 0$ and computing a loss as $L(x^{(0)}, \hat{x}^{(0)}_{\text{prev.}} = 0)$. The other 50% of the time, one (i) simulates an additional forward noising step to obtain $x^{(t+1)} \sim q(x^{(t+1)} \mid x^{(t)})$, (ii) computes $\hat{x}^{(0)}_{\text{prev.}} = \hat{x}^{(0)}(x^{(t+1)}, \hat{x}^{(0)}_{\text{prev.}} = 0)$, and (iii) computes a loss as $L(x^{(0)}, \hat{x}^{(0)}(x^{(t)}, \hat{x}^{(0)}_{\text{prev.}}))$, backpropagating gradients only through the second denoising step. Training and sampling with self-conditioning

Algorithm 2 RFdiffusion generation

function SAMPLEREFERENCE(L)
    ▷ Random initial structure for L residues
    for $l = 1, \ldots, L$ **do**
        $r_l^{(T)} \sim \text{Uniform}(SO(3))$
        $z_l^{(T)} \sim \mathcal{N}(0, I_3)$
        $x_l^{(T)} = (r_l^{(T)}, x_l^{(T)})$
    end for
return $x^{(T)}$
end function

function REVERSESTEP($x^{(t)}, \hat{x}^{(0)}$)
    ▷ One step of reverse diffusion
    for $l = 1, \ldots, L$ **do**
        $(r_l^{(t)}, z_l^{(t)}) = x_l^{(t)}$
        $(\hat{r}_l^{(0)}, \hat{z}_l^{(0)}) = \hat{x}_l^{(0)}$
        ▷ Update translations
        $z_l^{(t-1)} \sim \mathcal{N}(\frac{\sqrt{\bar{\alpha}^{(t-1)}}\beta^{(t)}}{1-\bar{\alpha}^{(t)}}\hat{z}_l^{(0)} + \frac{\sqrt{\alpha^{(t)}}(1-\bar{\alpha}^{(t-1)})}{1-\bar{\alpha}^{(t)}} z_l^{(t)}, \beta^{(t)} I_3)$

        ▷ Update rotations
        $s_l =$ROTATIONSCOREAPPROXIMATION($r_l^{(t)}, \hat{r}_l^{(0)}, \sigma_t^2$)
        $\epsilon_{l,1}, \epsilon_{l,2}, \epsilon_{l,3} \overset{iid}{\sim} \mathcal{N}(0,1)$
        $r_l^{(t-1)} = r_l^{(t)} \exp_{I_3}\left\{(\sigma_t^2 - \sigma_{t-1}^2){r_l^{(t)}}^\top s_l + \sqrt{\sigma_t^2 - \sigma_{t-1}^2}\sum_{d=1}^3 \epsilon_{l,d} f_d\right\}$
        $x_l^{(t-1)} = (r_l^{(t-1)}, z_l^{(t-1)})$
    end for
return $x^{(t-1)}$
end function

function SAMPLE(L)
    ▷ RFdiffusion generation of L-residue backbone structure
    $x^{(T)} = \text{SampleReference}(L)$
    $\hat{x}_{\text{prev.}}^{(0)} = \vec{0}$ ▷ Initialize self-conditioning
    for $t = T, \ldots, 1$ **do**
        $\hat{x}^{(0)} =$ RFDIFFUSION($x^{(t)}, \hat{x}_{\text{prev.}}^{(0)}$)
        $x^{(t-1)} =$ REVERSESTEP($x^{(t)}, \hat{x}^{(0)}$)
        $\hat{x}_{\text{prev.}}^{(0)} = \hat{x}^{(0)}$
    end for
return $\hat{x}^{(0)}$
end function

are described in Algorithms 2 and 3.

In RFdiffusion, we input $\hat{x}^{(0)}_{\text{prev.}}$ through the template structure feature (xyz_t, see Supplementary Methods Table 1) and we input $x^{(t)}$ as coordinates to the 3D track of RF (xyz_prev, see Supplementary Methods Table 1). Inputting $x^{(t)}$ as coordinates, as opposed to the distogram and anglegram used in the template structure feature, allows the network to keep the motif fixed in coordinate space.

2.5 Mean squared error loss on residue frames

The primary objective used to train RFdiffusion is mean squared error loss, averaged across training examples, time steps and the forward noising process,

$$\text{MSE}_{\text{Frame}} = \frac{1}{T}\sum_{t=1}^{T}\mathbb{E}_q[d_{\text{frame}}(x^{(0)}, \hat{x}^{(0)}(x^{(t)}))^2],$$

where

$$d_{\text{frame}}(x^{(0)}, \hat{x}^{(0)}) = \sqrt{\frac{1}{L}\sum_{l=1}^{L}\|z_l^{(0)} - \hat{z}_l^{(0)}\|_2^2 + \|I_3 - \hat{r}_l^{(0)\top} r_l^{(0)}\|_F^2},$$

is a metric on the sets of predicted frames consisting of Euclidean distance on the C_α coordinates ($\|z^{(0)} - \hat{z}^{(0)}\|_2$), and a metric on rotation matrices ($\|I_3 - \hat{r}^{0\top} r^{(0)}\|_F^2$, where $\|\cdot\|_F$ denotes the Frobenius norm [69]. In practice, we use slight modification of $\text{MSE}_{\text{Frame}}$ chosen to improve stability of training (see Section 4.1 for details).

In each training step, we compute an unbiased Monte Carlo estimate of this objective by sampling a time step $t \sim \mathcal{U}(1, \ldots, T)$, a single structure $x^{(0)}$ from our dataset, simulating the forward process to obtain $x^{(t)} \mid x^{(0)} \sim q(x^{(t)} \mid x^{(0)})$, and taking a gradient step on our slight modification of $\text{MSE}_{\text{Frame}}$. Algorithm 3 summarizes the training procedure.

Our choice of $\text{MSE}_{\text{Frame}}$ takes inspiration from [11]. In particular, Ho et al. [11, section 3.2] comment that when the forward process consists of adding Gaussian noise, the training objective

Algorithm 3 RFdiffusion Training

function FORWARDNOISE($x^{(0)}, t$)
  for $l = 1, \ldots, L$ **do**
    $(r_l^{(0)}, z_l^{(0)}) = x_l^{(0)}$
    $z_l^{(t)} \sim \mathcal{N}\left(\sqrt{\bar{\alpha}^{(t)}} z_l^{(0)}, (1 - \bar{\alpha}^{(t)}) I_3\right)$
    $r_l^{(t)} \sim \mathcal{IG}_{SO(3)}\left(r_l^{(0)}, \sigma_t^2\right)$
    $x_l^{(t)} = (r_l^{(t)}, z_l^{(t)})$
  end for
return $x^{(t)}$
end function

function TRAIN
  while not converged **do**
    $x^{(0)} \sim$ TrainingSet
    $t \sim$ Uniform($\{1, \ldots, T\}$)
    if Uniform$(0, 1.0) < 0.5$ **or** $t = T$ **then**
      ▷ Train step without self-conditioning
      $x^{(t)} =$ ForwardNoise($x^{(0)}, t$)
      $\hat{x}^{(0)}_{\text{prev.}} = \vec{0}$
    else
      ▷ Train step with self-conditioning
      $x^{(t+1)} =$ ForwardNoise($x^{(0)}, t + 1$)   ▷ Sample $(x^{(t+1)}, x^{(t)}) \sim q(x^{(t:t+1)} \mid x^{(0)})$
      $x^{(t)} =$ ReverseStep($x^{(t+1)}, x^{(0)}$)

      ▷ Compute self-conditioning input
      $\hat{x}^{(0)}_{\text{prev.}} = RFdiffusion(x^{(t+1)}, \vec{0})$
      $\hat{x}^{(0)}_{\text{prev.}} =$ StopGradient($\hat{x}^{(0)}_{\text{prev.}}$)
    end if
    $\hat{x}^{(0)} = RFdiffusion(x^{(t)}, \hat{x}^{(0)}_{\text{prev.}})$
    Take gradient step on $d_{\text{frame}}\left(x^{(0)}, \hat{x}^{(0)}\right)^2$
  end while
end function

of minimizing the Kullback-Leibler divergence of $q(z^{(t-1)} \mid z^{(t)})$ to $p(z^{(t-1)} \mid z^{(t)})$ can be rewritten as a rescaling of the expected squared error of a prediction of $z^{(0)}$ from noisy observations $z^{(t)}$. In particular if we fix the variance of the backward transitions to β_t as in Section 2.2, then for each t

$$\mathbb{E}_q[\mathrm{KL}(q(z^{(t-1)} \mid z^{(t)}) \| p(z^{(t-1)} \mid z^{(t)}))] = \mathbb{E}_q \left[\frac{\bar{\alpha}^{(t-1)}(1-\alpha_t)^2}{2\beta_t(1-\bar{\alpha}^{(t)})^2} \|z^{(0)} - \hat{z}^{(0)}(z^{(t)})\|_2^2 \right] + c, \tag{13}$$

where c is a constant that does not depend on p (see [70, Equation 99] and [11]). Consequently when one minimizes the right-hand-side of Equation (13) for every t, they maximize a weighted variational lower bound on the likelihood of the data. Moreover, this bound is globally minimized only when each $p(z^{(t-1)} \mid z^{(t)})$ matches $q(z^{(t-1)} \mid z^{(t)})$, and $p(z^{(0)})$ therefore matches the data-distribution [11]. Although Ho et al. [11] found better performance in generative modeling of images when predicting the noise added in the forward process (rather than $x^{(0)}$), we reasoned that by predicting $x^{(0)}$ we could better leverage the inductive biases of RoseTTAFold pre-trained for structure prediction to produce realistic structures.

However, the equivalence of learning to optimally denoise according to average squared distance and matching the reverse process is only known to apply when the forward process consists of Gaussian noise, and likely does not hold for the $\mathcal{IG}_{SO(3)}$ noise used for rotations. However the squared Frobenius norm metric seemed to be a sensible choice because (1) our chosen forward noising process for rotations is approximately Gaussian in the tangent space of $SO(3)$ at $r^{(0)}$ for t close to zero, and (2) this metric is approximately equal to a scaling of squared Euclidean distance in the tangent space of $SO(3)$ when $\hat{r}^{(0)}$ is close to $r^{(0)}$ [71].

2.6 Geometric invariances and RFdiffusion

RFdiffusion leverages the SE(3)-equivariance of RoseTTAFold to parameterize a distribution over protein backbones that is invariant to rotation. In this subsection, we describe why this invariance property is desirable, and how it is conferred by the SE(3)-equivariance of RoseTTAFold.

Our goal of achieving rotational invariance builds on previous work [27, 5], and is motivated by the observation that biochemical characteristics of proteins are conferred by relative geometry of the atoms which comprise them. However, when we describe the structure of protein backbone as a collection of rigid bodies parameterized by $C\alpha$ coordinates and per-residue $N{-}C\alpha{-}C$ orientations, our description invariably relies on the choice of a semantically arbitrary coordinate system. A naive approach to learning a distribution over protein backbones might assign different probabilities to the same structure when viewed from different angles. By contrast, we seek to model any protein structure as equally likely upon a rigid rotation. Indeed, prior work has established that imposing geometric invariances in neural networks imparts inductive biases that can improve generalization and training efficiency [72].

Invariance to translation has also been considered in generative modeling of proteins. While one approach to addressing translational invariance would be to explicitly parameterize an SE(3)-invariant measure, this introduces the challenge of contending with unnormalized measures because no normalized probability distribution can be invariant to translation. In practice, we obviate this challenge by centering all training examples at the origin to eliminate the degrees of freedom corresponding to translation, and considering only invariance to rotation; as demonstrated by Yim et al. [64, proposition 3.5], this does not sacrifice any generality because any SE(3)-invariant measure on $\mathrm{SE}(3)^L$ may be represented a rotationally invariant probability measure on rigid bodies with center of mass set to the origin.

More formally, our goal is ensure that for any structure x and rotation R, $p(x) = p(R * x)$, where p denotes the density parameterized by the model and $R * x = [R * x_1, \ldots, R * x_L] = [(Rr_1, Rz_1), \ldots, (Rr_N, Rz_L)]$ describes the structure obtained by rotating x about the origin by R. To enforce invariance of p with respect to rotations in our DDPM, we follow prior work [73, 5] by (1) using a rotation invariant reference distribution (satisfying $p(x^{(T)}) = p(R * x^{(T)})$ for every $R \in SO(3)$) and (2) constraining the reverse transitions to be rotationally equivariant, i.e. to satisfy $p(x^{(t-1)}|x^{(t)}) = p(R * x^{(t-1)}|R * x^{(t)})$. Criterion (1) is readily satisfied by the choices of the

zero mean Gaussian with isotropic covariance as the reference distribution for translations, and the uniform distribution on $SO(3)$ for rotations.

That criterion (2) above is satisfied owes to the SE(3) equivariance of the denoising network inherited from RoseTTAFold. In particular, because by construction of the reverse process, for any $x^{(t-1)}, x^{(t)}$, and R

$$p(R * x^{(t-1)} | R * x^{(t)}) = \prod_{l=1}^{L} p(R * z_l^{(t-1)} | R * x^{(t)}) p(R * r_l^{(t-1)} | R * x^{(t)}),$$

we see that it is sufficient to show independently for translations and rotations that the distributions sampled in the reverse process are rotationally equivariant. For translations, because RoseTTAFold's prediction $\hat{z}^{(0)}$ is rotationally equivariant with respect to $x^{(t)}$, and because $\hat{\mu}(x^{(t)})$ in Equation (2) is a linear combination of $\hat{z}^{(0)}$ and $z^{(t)}$, $\hat{\mu}(x^{(t)})$ and therefore also $p(z^{(t-1)} \mid x^{(t)})$ are equivariant with respect to $x^{(t)}$.

For rotations, we can confirm equivariance by noticing that the update in Equation (10) for each residue l is computed as the matrix exponential of a rotationally invariant quantity multiplied by $r_l^{(t)}$. In particular, the drift term in Equation (10) may be rewritten as

$$r^{(t)\top} \nabla_{r^{(t)}} \log \mathcal{IG}_{SO(3)}(r^{(t)}; \hat{r}^{(0)}, \sigma_t^2) = \tilde{r}^\top \nabla_{\tilde{r}} \log \mathcal{IG}_{SO(3)}(\tilde{r}; I_3, \sigma_t^2),$$

for $\tilde{r} = \hat{r}^{(0)\top} r^{(t)}$, which is rotationally invariant because $\hat{r}^{(0)}$ is rotationally equivariant.

3 RFdiffusion: methodology for controlled design

We next describe several techniques to *control* generation of backbones in RFdiffusion to meet specific design criteria. Section 3.1 describes generation of symmetric oligomers. Section 3.2 describes our approach to training RFdiffusion for generation via conditional training. Section 3.3 then describes how we modify the architecture of and fine-tune RFdiffusion for targeted binder design, and design subject to topology constraints. Finally, Section 3.4 describes how we can guide generation with extrinsically defined "potentials".

3.1 Generation of oligomers with point group symmetries

As discussed in the main text, generating oligomeric assemblies obeying desired point-group symmetry constraints is crucial in several design contexts. Point group symmetries may be represented by a finite collection of rotation matrices that form a mathematical group with respect to matrix multiplication as the group operation [36]. For example, we may represent the cyclic symmetry group of order K by the set of rotation matrices that rotate increments of $(360/K)^o$ about the z-axis, $C_k = \{R_z^{(k/K)360^o}\}_{k=0}^{K-1}$. Analogous representations exist for all other point groups (including dihedral, octahedral, tetrahedral, and icosahedral). Without loss of generality we set the first rotation to be the identity $R_1 = I_3$. We represent an oligomer with K monomer subunits each with L residues by $X = [x^1, \ldots, x^K]$ where each subunit k consists of the translations and rotations $x^k = ([z_1^k, \ldots, z_L^k], [r_1^k, \ldots, r_L^k])$. Then we say an oligomer obeys a point group symmetry $\mathcal{R} = \{R_1, \ldots, R_k\}$, if $X = [R_1 {*} x^1, \ldots, R_K {*} x^1]$ where $R {*} x_1 = ([R {*} z_1^k, \ldots, R {*} z_L^k], [R {*} r_1^k, \ldots, R {*} r_L^k])$ denotes the rotation of a monomer backbone structure by R.

Previous work has demonstrated some success generating designs with symmetry through Hallucination [6, 7] with the inclusion of penalty terms on the deviation of predicted structures from the desired symmetry, but this work suffered from large computational cost (on the order of 1 GPU

Algorithm 4 Generation of symmetric oligomers

function SampleSymmetric($M, \mathfrak{R} = \{R_k\}_{k=1}^{K}$)
  ▷ RFDiffusion generation of oligomer with symmetry $\mathfrak{R}$
  $x^{(T,1)} = \text{SampleReference}(M)$
  for $t = T, \ldots, 1$ **do**
    $X^{(t)} = [R_1 x^{(t,1)}, \ldots, R_K x^{(t,1)}]$ ▷ Symmetrize chains
    $\hat{X}^{(0)} = RFdiffusion(X^{(t)})$
    $[x^{(t-1,1)}, \ldots, x^{(t-1,K)}] = \text{ReverseStep}(X^{(t)}, \hat{X}^{(0)})$
  end for
return $\hat{X}^{(0)}$
end function

day per design) and low success rates, presumably due to the inability to precisely control the desired symmetry [7]. We hypothesized that RFdiffusion by contrast could provide improved control over symmetries in design by maintaining symmetry in denoising predictions, and by allowing us to enforce hard symmetry constraints during the reverse process (Algorithm 4).

Although we do enforce exact symmetry through explicit symmetrization at each denoising step, we observed that RFdiffusion provides predictions of the denoised oligomer structures that preserve the desired symmetry nearly exactly, even in the first denoising steps (Extended Data Fig. 5A). This property of denoised predictions owes to the exact equivariance of RoseTTAFold with respect to global rotations and the approximate equivariance with respect to permutation (i.e. relabeling) of chains. In particular, Proposition 2 guarantees that rotation and permutation equivariance of a neural network are sufficient conditions for maintenance of point group symmetries in the neural network's output. In RFdiffusion, exact rotation equivariance is inherited from the SE(3)-transformer architecture used in the structure module of RoseTTAFold [62]. Permutation equivariance by contrast arises if the intermediate representations and outputs for each residue are unaffected by the ordering of chains. This is nearly the case with RFdiffusion, with the exception that the RoseTTAFold pair representation contains directional sequence distance feature inputs for each pair of residues, clipped between -32 and 32 residues away; since oligomers are presented to RoseTTAFold by incrementing the sequence position index at the start of each chain, the sign of

these features breaks exact permutation equivariance. However, we find empirically that deviation from exact symmetry in RFdiffusion predictions is minimal (Extended Data Fig. 5A).

Proposition on preservation of symmetry: We next provide a proposition that more precisely illuminates the mechanism by which predictions of denoised structures maintain the desired symmetry at each step.

Proposition 2. *Consider any function* $F : [x_1, \ldots, x_K] \to [y_1, \ldots, y_K]$ *and point group symmetry* $\mathcal{R} = \{R_1, \ldots, R_K\}$. *If* F *is both*

1. *rotation equivariant, that is* $F([R * x_1, \ldots, R * x_K]) = [R * y_1, \ldots, R * y_K]$ *for every rotation matrix* R*, and*

2. *permutation equivariant, that is* $F([x_{\sigma(1)}, \ldots, x_{\sigma(K)}]) = [y_{\sigma(1)}, \ldots, y_{\sigma(K)}]$ *for every permutation* σ*,*

then F *is symmetry preserving. In particular, for any* x, $F([R_1*x, \ldots, R_K*x]) = [R_1*y, \ldots, R_K*y]$ *for some* y.

Notably, Proposition 2 holds for any neural network satisfying assumptions on F above. We now prove the proposition.

Proof. We first establish some basic properties about permutations of point groups. First note that every member $R_k \in \mathcal{R}$ defines a permutation of $\mathcal{R}$ since $\{R_kR_1, R_kR_2, \ldots, R_kR_K\} = \mathcal{R}$. Let σ_k denote the permutation associated with $R_1R_k^T \in \mathcal{R}$. In particular, σ_k is the permutation such that for each m, $R_{\sigma_k(m)} = (R_1R_k^T)R_m$. Notably, $\sigma_k(k) = 1$ because $R_{\sigma_k(k)} = (R_1R_k^T)R_k = R_1$. For any permutation σ, we let $\bar{\sigma}$ denote its inverse, the permutation such that $\bar{\sigma}(\sigma(k)) = k$ for every k. Lastly, note that for $R_{\bar{\sigma}_k(m)} = (R_kR_1^T)R_m$, and so $R_{\bar{\sigma}_k(1)} = (R_kR_1^T)R_1$.

Assume without loss of generality that $F([R_1*x, \ldots, R_K*x])_1 = R_1*y$. To prove the proposition, it suffices to show that for any k, $F([R_1 * x, \ldots, R_k * x])_k = R_k * y$. Consider σ_k as defined above. We can write

$$
\begin{aligned}
F([R_1 * x, \ldots, R_K * x])_k &= F(R_{\bar{\sigma}_k(1)}x, \ldots, R_{\bar{\sigma}_k(K)}x)_{\sigma_k(k)} \\
&= F((R_kR_1^T)R_1x, \ldots, (R_kR_1^T)R_Kx)_1
\end{aligned}
$$

where the first equality follows from permutation equivariance of F, and the second equality follows from the definitions of σ_k and $\bar{\sigma}_k$. Finally, by the rotation equivariance of F,

$$
\begin{aligned}
F((R_k R_1^T)R_1 x, \ldots, (R_k R_1^T)R_K x)_1 &= (R_k R_1^T)F(R_1 x, \ldots, R_K x)_1 \\
&= (R_k R_1^T)R_1 y = R_k y.
\end{aligned}
$$

Therefore $F(R_1 x, \ldots, R_k x)_k = R_k y$ as desired. □

3.2 Conditional training for functional-motif scaffolding

Our approach to scaffolding functional motifs with RFdiffusion follows Trippe et al. [5], who treat motif-scaffolding as a conditional generative modeling problem. We partition the residues of a structure into the residues comprising the *motif* and those comprising the remainder of the backbone, which we refer to as the *scaffold* that supports it. For a structure with L residues, we let $\mathcal{M}$ denote the (potentially discontiguous) set of indices corresponding to the motif and $\mathcal{S}$ be the remaining scaffold indices, such that the union of $\mathcal{M}$ and $\mathcal{S}$ is the set of indices up to L (i.e. $M \cup S = \{1, \ldots, L\}$).

We write $x_{\mathcal{M}}$ to denote the structure of the motif residues and $x_{\mathcal{S}}$ to be the scaffold residue frames such that we may write the whole (un-noised) protein structure as $x^{(0)} = [x_{\mathcal{M}}^{(0)}, x_{\mathcal{S}}^{(0)}]$. Our goal is to sample scaffold backbones from the conditional distribution $q(x_{\mathcal{S}}^{(0)} \mid x_{\mathcal{M}}^{(0)})$. To do this, we aim to learn the reversal of the forward noising process applied only to scaffold residues, with the motif held fixed, $p(x_{\mathcal{S}}^{(t-1)} \mid x_{\mathcal{S}}^{(t)}, x_{\mathcal{M}}^{(0)}) \approx q(x_{\mathcal{S}}^{(t-1)} \mid x_{\mathcal{S}}^{(t)}, x_{\mathcal{M}}^{(0)})$, where $q(x_{\mathcal{S}}^{(t-1)} \mid x_{\mathcal{S}}^{(t)}, x_{\mathcal{M}}^{(0)})$ is the conditional forward noising process described in Sections 2.2 and 2.3.

In earlier work, Wang et al. [4] demonstrated that RoseTTAFold may be trained to respect motif constraints provided as inputs through the template structure input features through retraining. Because the division of residues into motif and scaffold is specific to each design problem, we desired to train RFdiffusion such it may be used for any location of the motif within the sequence.

To this end, we took an amortized training approach, wherein for each motif-scaffolding training example we 1) begin with a structure $x^{(0)}$, 2) choose a random division into motif and scaffold $x^{(0)} = [x^{(0)}_{\mathcal{M}}, x^{(0)}_{\mathcal{S}}]$ (see Section 4.1 for details), 3) apply noise to the scaffold to obtain $x^{(t)}_{\mathcal{S}} \sim q(x^{(t)}_{\mathcal{S}} \mid x^{(0)}_{\mathcal{S}})$, and 4) compute a loss on the RFdiffusion prediction $\hat{x}^{(0)}([x^{(0)}_{\mathcal{M}}, x^{(t)}_{\mathcal{S}}])$ of $x^{(0)} = [x^{(0)}_{\mathcal{M}}, x^{(0)}_{\mathcal{S}}]$. In order to encourage RFdiffusion to not move the motif, we set the time-step input for motif residues to $t = 0$, we include both the motif and the scaffold residues when we compute the loss on the prediction. We center motif-scaffolding training examples on the center of mass of $x^{(0)}_{\mathcal{M}}$; if the training example is instead centered at the center of mass of the full chain, information is leaked about the relative position of the bulk of $x^{(t)}_{\mathcal{S}}$ relative to $x^{(0)}_{\mathcal{M}}$.

Because motif sidechain geometry is crucial for most motif-scaffolding problems, we additionally provide the amino acid sequence and sidechain torsion angles for motif residues as inputs to RFdiffusion (provided through RoseTTAFold's template feature inputs). Overall this strategy is akin to the diffusion model inpainting training and generation described by Saharia et al. [74], who use randomly generated image masks.

In summary, generation of scaffolds conditional on a motif with RFdiffusion differs from unconditional generation only in (1) the inclusion of noise-free motif backbone and sidechain structure in the template inputs and (2) replacement of the motif backbone coordinates in $x^{(t)}$ with un-noised motif coordinates at each step, (3) setting of the timestep for motif residues to 0, and (4) centering examples based on $x^{(0)}_{\mathcal{M}}$ rather than based on $x^{(0)}$.

3.3 Fine-tuning and architecture modifications

We wish to train a diffusion model which can condition on arbitrary features; to accomplish this, additional features must be input to RFdiffusion beyond the features already taken by RoseTTAFold. Given the vast difference in performance between models trained from scratch versus those initialized from RF weights (Extended Data Fig. 1F), we also wish to continue to initialize training

from RF weights. To allow the addition of more features, we choose to expand the size of existing features in RF and to expand the corresponding size of the weights of the embedding layer which initially embeds the feature into the model. We initialize the weights associated with these newly added dimensions in these embedding layers to zero. With this initialization, the model gives exactly the same output as unmodified RF with the initial weights. Upon training, the model can then learn to use the newly expanded features.

In Section 4.3, we describe how we do this in detail for binder design, by controlling secondary structure adjacency and fold family of designs and directing generated binders to target hotspots. In addition to architectural modifications, RFdiffusion can also be further fine-tuned on different conditional tasks; we describe how we have done this for improved scaffolding of functional motifs in Section 4.2.

3.4 Guiding RFdiffusion inference with external potentials

In addition to the network's ability to condition on structural motifs, the inference process can be guided by external potential functions to generate proteins which possess arbitrary desired properties, such as the existence of contacts with another protein or a desired surface concavity. Previous work has demonstrated that diffusion models can be made to sample from conditional distributions $p(x^{(0)} \mid y)$ without retraining if given a classifier able to operate on noisy samples, $p(y \mid x^{(t)})$ [65, Appendix I]. In particular, $p(y = 1 \mid x^{(t)})$ may be understood as a predicted probability that an example $x^{(0)}$ has a property of interest (or is in a given "class") given only the noised observation $x^{(t)}$. In contrast to unguided generation, wherein one noisily moves in the direction $\nabla_{x^{(t)}} \log p(x^{(t)})$ (which points toward $\hat{x}^{(0)}$), with guidance one instead follows $\nabla_{x^{(t)}} \log p(x^{(t)}) + \nabla_{x^{(t)}} \log p(y = 1 \mid x^{(t)})$ in the reverse step[65].

In the present work, we construct heuristic approximations of these classification log probabilities $P(x^{(t)}) \approx \log p(y = 1 \mid x^{(t)})$ for two protein conditional generation objectives, symmetric

oligomer design (Fig. 3, Extended Data Fig. 5) and enzyme design with concave pockets (Extended Data Fig. 6E-H). We show how to incorporate them into the sampling procedure in Algorithm 5, and detail the functional forms of these potentials in Section 4.4. In this work, we consider potentials the are defined as a function of the C_α coordinates alone, and so (in each individual step) these potentials do not impact residue orientations.

Algorithm 5 Generation with guidance

```
function SampleGuided(L, P, GuideScale)
    ▷ Generation of L-residue backbone structure, guided by potential P
    x^(T) = SampleReference(M)
    for t = T, ..., 1 do
        x̂^(0) = RFDiffusion(x^(t))
        x^(t−1) = ReverseStep(x^(t), x̂^(0))
        x^(t−1) = x^(t−1) + GuideScale(t)∇_{x^(t)} P(x^(t))        ▷ Apply guidance
    end for
return x̂^(0)
end function
```

3.5 Tuning diversity by scaling noise at inference

For some problems, reported throughout the manuscript, we reduce the noise added at each step, by including a multiplicative factor to the variance of the noise. Typically, this improves design quality, at the expense of design diversity.

4 RFdiffusion: training and fine-tuning

Sections 2 and 3 described key aspects of our formulation of RFdiffusion and how we have approached using it to generate designs with desired properties. In this section, we provide precise details on RFdiffusion training. Section 4.1 details the inputs to and outputs of a "base" version of RFdiffusion; these inputs and outputs are adapted from their uses in RoseTTAFold (Section 1.2). Section 4.1 also describes the precise losses used and other training information. Section 4.2 provides details of the variant of RFdiffusion fine-tuned on an enzyme active site-scaffolding task. Section 4.3 describes modifications to and funetuning of RFdiffusion for design of protein-protein interactions. Finally, Section 4.4 describes two specific instances of guiding potentials. We subsequently present final details of how we applied RFdiffusion and these fine-tuned variants to specific design tasks in Sections 5 and 6.

4.1 RFdiffusion base model

RFdiffusion was trained on monomer structures in the PDB used for RoseTTAFold training. Training examples consist of the unconditional task 20% of the time and the motif-conditional task 80% of the time. For the motif-conditional task, a contiguous set of residues is selected as the motif, and the true sequence and structure are provided to the model. RFdiffusion is trained starting from the final RF weights. RFdiffusion does not use recycling.

Losses: RFdiffusion was trained with a loss comprising two terms,

$$\mathcal{L}_{\text{Diffusion}} = \mathcal{L}_{\text{Frame}} + w_{\text{2D}}\mathcal{L}_{\text{2D}},$$

Input name (Shape)	Description
`msa_masked` (1,1, L, 48)	The truncated MSA now contains only the masked sequence (20aa, zeros (1), mask (1), repeat aa (20), zeros (1), repeat mask (1), zeros (2), N-term/C-term (2))
`msa_full` (1, 1, L, 25)	The full MSA now contains only the masked sequence (20aa, zeros (1), mask (1), zeros (1), N-term/C-term (2))
`seq` (1, L, 22)	The masked sequence (20aa, zeros (1), mask (1))
`xyz_prev` (L, 27, 3)	The coordinates of all atoms (N-Ca-C-O backbone (4), (up to) 10 sidechain atoms, (up to) 13 hydrogen atoms)
`idx_pdb` (L)	The integer index of each residue. Used to assign each residue its neighboring residue. This feature has the same definition as in RF
`t1d` (1, L, 22)	The one-dimensional features associated with $x^{(t)}$ (20 amino acids, mask (1), timestep (1)). The timestep is set to 1 for all fixed motif residues, and to $1 - \frac{t}{T}$ in all other positions.
`t2d` (1, L, L, 44)	The two-dimensional features associated with $x^{(t)}$ structure. These features are computed from $x^{(t)}$, not from $\hat{x}^{(0)}_{\text{prev.}}$. (36 distance bins (2-20Å, 0.5Å bins) + 1 final distance bin (> 20Å), angle maps (sine and cosine of omega, theta and phi angle) (6), missing residue mask (1))
`xyz_t` (1, L, 27, 3)	The self-conditioning feature. As described in Section 2.4, this is $\hat{x}^{(0)}_{\text{prev.}}$. This feature is immediately converted to a distogram and anglegram representation by the model. (N, C_α, C backbone atoms)
`alpha_t` (1, L, 30)	The sidechain torsions of the motif region of $x^{(t)}$. For positions with a masked sequence, zeros are provided. Initially T, L, 10, 2, with sine and cosine of (omega, phi, psi angles (3), (up to) 4 torsion angles, C_β bend (1), C_β twist (1), C_γ bend (1)). This is concatenated with a mask (T, L, 10, 1) indicating which torsion angles are present for a given amino acid, and reshaped to T, L, 30.
`msa_prev` (1, L, Cm)	The MSA embedding recycling information. This is the model's previous embedding at each position in the masked sequence. Cm = 256
`pair_prev` (L, L, Cp)	The 2-D embedding recycling information. This is the model's previous embedding at each edge between each node. Cp = 128
`state_prev` (L, Cs)	The 1-D embedding recycling information. This is the model's previous embedding at each position in the query sequence. Cs = 16

Supplementary Methods Table 4: Description of features input to RFdiffusion.

Output name (Shape)	Description
msa (1, L, Cm)	The model's final embedding at each position in the masked sequence. Cm = 256
pair (L, L, Cp)	The model's final embedding at each edge between each node. Cp = 128
state (L, Cs)	The model's final embedding at each position in the query sequence. Cs = 16
xyz (L, 27, 3)	The model's full-atom prediction of structure (N-Ca-C-O backbone (4), (up to) 10 sidechain atoms, (up to) 13 hydrogen atoms)

Supplementary Methods Table 5: Outputs returned by RFdiffusion.

where $\mathcal{L}_{\mathrm{Frame}}$ is a modified variant of squared distance loss in $\mathrm{MSE}_{\mathrm{Frame}}$ (Section 2.5), $\mathcal{L}_{\mathrm{2D}}$ is a distogram and anglegram loss, and w_{2D} is a weighting factor. We now describe each loss.

$\mathcal{L}_{\mathrm{Frame}}$ includes two modifications from $\mathrm{MSE}_{\mathrm{Frame}}$ intended to improve the stability of optimization. First, whereas $\mathrm{MSE}_{\mathrm{Frame}}$ relies on a distance computed simply as sum of squared distances defined on the translation and rotation components of residue frames, $\mathcal{L}_{\mathrm{Frame}}$ relies on a weighted sum of these components that includes clamping on translation distance,

$$d_{\mathrm{Frame}}(x^{(0)}, \hat{x}^{(0)}) = \sqrt{\frac{1}{L}\sum_{l=1}^{L}\left(w_{\mathrm{trans}}\min(\|z_l^{(0)} - \hat{z}_l^{(0)}\|_2, d_{\mathrm{clamp}})^2 + w_{\mathrm{rot}}\|I_3 - \hat{r}_l^{(0)\top} r_l^{(0)}\|_F^2\right)},$$

where w_{trans} and w_{rot} are weights on the rotation and translation distances, and d_{clamp} is a maximum distance above which translation distances are clamped. Note that the translation distance is only clamped 90% of the time. Second, $\mathcal{L}_{\mathrm{Frame}}$ includes contributions from $d_{\mathrm{Frame}}(x^{(0)}, \hat{x}^{(0)})$ computed at each intermediate structure module iteration with an exponential weighting, γ that places greater importance on later outputs. In particular, we have

$$\mathcal{L}_{\mathrm{Frame}} = \frac{1}{\sum_{i=0}^{I-1}\gamma^i}\sum_{i=1}^{I}\gamma^{I-i} d_{\mathrm{Frame}}(x^{(0)}, \hat{x}^{(0),i})^2$$

where $\hat{x}^{(0),i}$ is the ith structure block output.

The second term in the loss, $\mathcal{L}_{\mathrm{2D}}$, is inspired by trRosetta [63]. In contrast to the definition-

ally unimodal structure track outputs, the model outputs multimodal distributions of expected distances, dihedral angles, and planar angles between all pairs of contacting residues. $D_{:,l,l'}$, $\Omega_{:,l,l'}$, $\Phi_{:,l,l'}$, $\Theta_{:,l,l'}$, together describe the orientation of residue l relative to residue l'. The following loss consists of the cross entropy between the one-hot histogram of the known inter-residue distances and orientations and the corresponding distributions predicted by the model.

$$\begin{aligned}\mathcal{L}_{\text{2D}}(\text{logits}_d, \text{logits}_\omega, \text{logits}_\theta, \text{logits}_\phi, z_0) =& \text{CrossEntropy}(\text{logits}_{\text{dist}}, D)+ \\ & \text{CrossEntropy}(\text{logits}_\omega, \Omega)+ \\ & \text{CrossEntropy}(\text{logits}_\theta, \Theta)+ \\ & \text{CrossEntropy}(\text{logits}_\phi, \Phi)\end{aligned}$$

where:

$$D \in \mathbf{R}^{[\mathbf{C_{dist}xLxL}]}; D_{b,l,l'} = \mathbb{1}[\text{bin}_{D,b}^{low} \le \max(\|C_{\beta,l'} - C_{\beta,l'}\|_2, 18.5) < \text{bin}_{D,b}^{high}]$$

$$\Omega \in \mathbf{R}^{[\mathbf{C_{dist}xLxL}]}; \Omega_{b,l,l'} = \mathbb{1}[\text{bin}_{\Omega,b}^{low} \le \text{Dihedral}(C_{\alpha,l}, C_{\beta,l}, C_{\alpha,l'}, C_{\beta,l'}) < \text{bin}_{\Omega,b}^{high}]$$

$$\Theta \in \mathbf{R}^{[\mathbf{C_{dist}xLxL}]}; \Omega_{b,l,l'} = \mathbb{1}[\text{bin}_{\Theta,b}^{low} \le \text{Dihedral}(N_{\alpha,l}, C_{\alpha,l}, C_{\beta,l}, C_{\beta,l'}) < \text{bin}_{\Theta,b}^{high}]$$

$$\Phi \in \mathbf{R}^{[\mathbf{C_{phi}xLxL}]}; \Omega_{b,l,l'} = \mathbb{1}[\text{bin}_{\Phi,b}^{low} \le \text{Planar}(C_{\alpha,l}, C_{\beta,l}, C_{\beta,l'}) < \text{bin}_{\Phi,b}^{high}]$$

and the bin edges for converting these angles and distances into a one-hot distribution are given by:

$$\begin{aligned}\text{bin}_{D,i} &= [\frac{i}{2}, \frac{i+1}{2}] \\ \text{bin}_{\Omega,i} = \text{bin}_{\Theta,i} &= [-\pi + \frac{2\pi i}{37}, -\pi + \frac{2\pi(i+1)}{37}] \\ \text{bin}_{\phi,i} &= [\frac{\pi i}{19}, \frac{\pi(i+1)}{19}].\end{aligned}$$

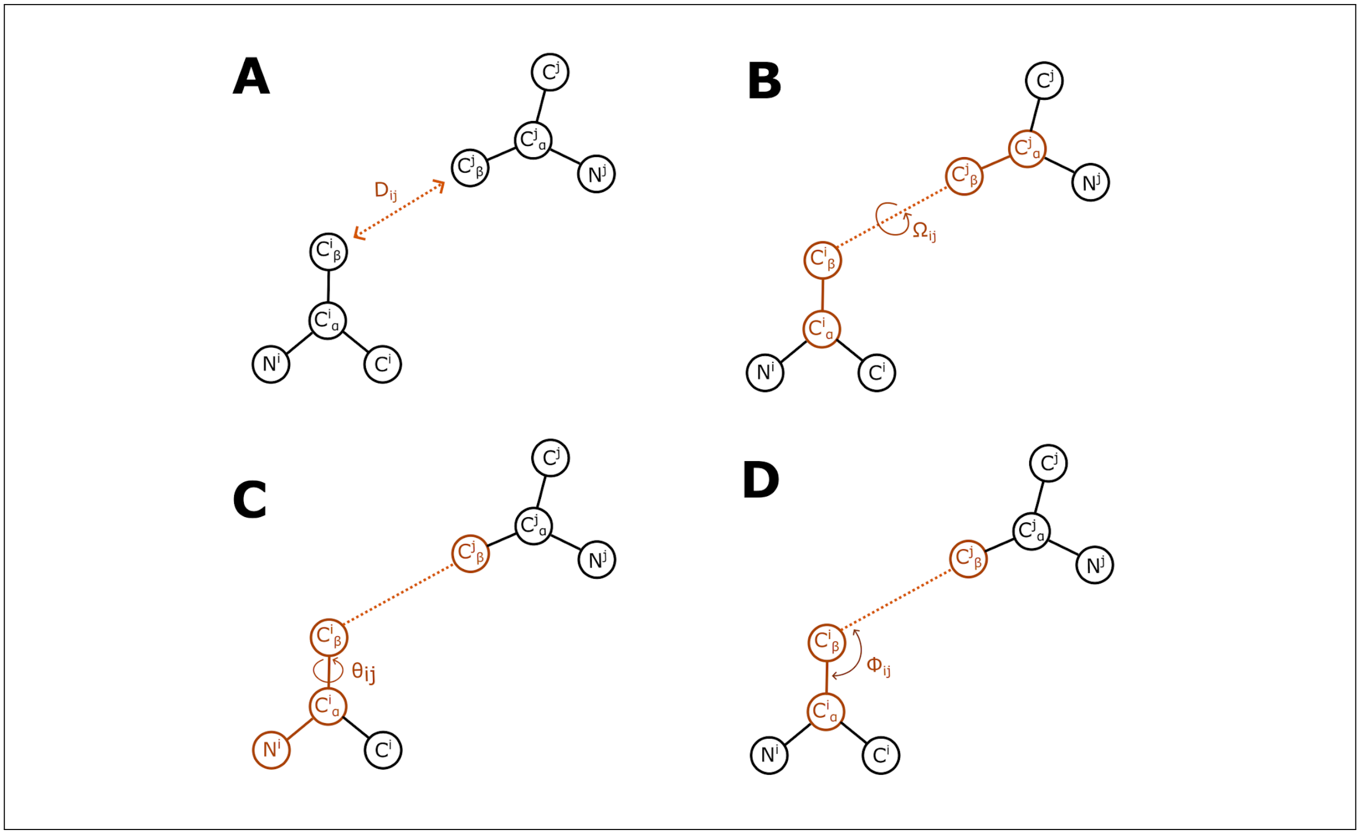

Supplementary Information Table 2: Diagrams for how to compute the four inter-residue distance and dihedral degrees of freedom.

And the formulae for computation of dihedral and planar angles are given by

$$\begin{aligned}\text{Dihedral}(a,b,c,d) &= \text{atan2}(\\ &\quad [c-b]\cdot(([b-a]\times[c-b])\times([c-b]\times[d-c])),\\ &\quad \|c-b\|([b-a]\times[c-b])\cdot([c-b]\times[d-c]))\\ \text{Planar}(a,b,c) &= \arccos(\frac{(a-b)\cdot(c-b)}{\|a-b\|\|c-b\|}).\end{aligned}$$

Motif-centering during training: For both training and scaffold generation, we center the motif at the origin. In preliminary computational experiments, we found that a model trained

with non-centered motifs extracted from structures that were globally centered exhibited biased motif placement. In particular, for motifs not placed at the origin we found that the scaffolds sampled from this model typically placed most residues towards the side of the motif oriented towards the origin. We interpreted this as subtle instance of undesirable label–leakage, and so trained RFdiffusion with motifs centered at the origin to correct this.

Once the distance loss is computed on the motif, motif coordinates are detached from the computation graph. As such, predicted motif coordinates are treated as constants for the purpose of all other loss comptutations. While this does not affect the values of any losses, this choice ensures that the components of loss on which gradients are computed with respect to the predicted motif coordinates are minimized when by predictions which doe not move from its initial coordinates. We made this choice to prevent the possibility that other computed losses would drive the motif from its (desired) initialization, but have not thoroughly explored this choice empirically.

Hyperparameters and coordinate scaling: We train RFdiffusion using the hyperparameters in Supplementary Methods Table 6. Although the coordinate inputs and outputs of RoseTTAFold are in units of Angstroms, we define the diffusion process in a downscaled space by dividing all coordinate values of $x^{(t)}$ and $x^{(0)}$ before performing each diffusion step by a factor of 4 (chosen empirically), and then scaling back up to Angstroms.

Training time: RFdiffusion trained to convergence when initialized from RF weights in 5 epochs. This took 3 days on 8 NVIDIA A100 GPUs.

4.2 Enzyme active site scaffolding by fine-tuning on minimal motifs

The version of RFdiffusion fine-tuned for enzyme active site scaffolding is trained starting from the base version of RFdiffusion. During fine-tuning 30% of tasks are from the base model task

Parameter name	Value
Crop size	384
Pseudo-batch size	64
w_{trans}	0.5
w_{rot}	1.0
w_{2D}	1.0
d_{clamp}	10
p_{clamp}	0.9
Structure block iteration decay rate γ	0.99
Learning rate	0.0005, No warm-up. Decay learning rate by 0.95 after every 10000 optimization steps.
Examples per epoch	25600
Number of diffusion timesteps (T)	200
Variance schedule for translations	$\beta^{(t)} = \beta^z_{min} + (\frac{t}{T})(\beta^z_{max} - \beta^z_{min})$ with $\beta^z_{min} = 0.01$ and $\beta^z_{max} = 0.07$.
Variance schedule for rotations	$\sigma_t = \sigma_{min} + \frac{t}{T}\beta^r_{min} + \frac{1}{2}(\frac{t}{T})^2(\beta^r_{max} - \beta^r_{min})$, with $\sigma_{min} = 0.02$, $\beta^r_{min} = 1.06$, and $\beta^r_{max} = 1.77$
Fraction of protein residues masked (when motif is provided)	Randomly picked from a uniform distribution between 20% and 100%, inclusive.
Probability of motif being contiguous or discontiguous	0.5
Probability of providing self-conditioning information	0.5
Coordinate scaling	0.25

Supplementary Methods Table 6: RFdiffusion training hyperparameters.

set Supplementary Methods (Table 6) and the other 70% are a "triple-contact" task, in which a random set of 3 residues all > 10 residues apart in sequence space but with pairwise C_β–C_β distances < 6Å is selected to form a model "active site". These three residues are included in the motif, and for each, there is a 50% chance of including one flanking residue. If no such triad is found in the monomer (as is the case for approximately 23% of training PDBs), the task would fall back to the base model training task. With the base RFdiffusion model, we note that the model sometimes fails to keep very small input motifs fixed in the output structures. As such, for this enzyme active site model, the motif-specific displacement loss is upweighted by a factor of 10 to encourage the network to keep the motif fixed, in order to compensate for the fact that otherwise motif recapitulation would comprise a significantly lower portion of the overall loss due to the much shorter motif length in this task. The network was fine-tuned for 5 epochs in this manner.

4.3 Architectural modifications for protein-protein interaction design

In this subsection, we describe RFdiffusion architecture modifications to incorporate target "hotspots" (Section 4.3.1) and desired binder topology (Section 4.3.2), and detail fine-tuning training (Section 4.3.3) for protein-protein interaction (PPI) design.

4.3.1 Protein–protein interface hotspots

When designing protein–protein interfaces, it is critical to be able to control the area of the target (fixed) protein to which the designed binding (diffused) protein should associate. To allow this control, we train the model to perform complex design conditioned on "interface hotspot" residues. We define an interface hotspot residue as any residue on the target (fixed) chain of the example that is within 10Å C_β–C_β distance of the binder (diffused) chain.

The 1-D one-hot tensor of interface hotspot residues is concatenated to RF's 1-D template feature (t1d, Supplementary Methods Table 1). We train two models for complex design, one which

Input name (shape)	Description
`t1d` (1, L, 24)	The one-dimensional features associated with $x^{(t)}$. A concatenation of one hot amino acids (20 features), mask (1 feature), timestep (1 feature), repeat mask (1 feature), hotspot (1 feature).

Supplementary Methods Table 7: RFdiffusion input feature modified for fine-tuning on complexes.

Input name (shape)	Description
`t1d` (1, L, 28)	The one-dimensional features associated with $x^{(t)}$ (20 amino acids, mask (1), timestep (1), repeat mask (1)[1], hotspot (1), secondary structure {"helix", "sheet", "loop", "mask"} (4))
`t2d` (1, L, L, 44)	The two-dimensional features associated with $x^{(t)}$ structure. These features are computed from $x^{(t)}$, not from $\hat{x}^{(0)}_{\text{prev.}}$. (36 distance bins (2–20Å, 0.5Å bins) + 1 final distance bin (> 20Å), angle maps (sine and cosine of omega, theta and phi angle) (6), missing residue mask (1), secondary structure {"adjacent", "non-adjacent", "mask"} (3))

Supplementary Methods Table 8: Modified features used in fine-tuning on complexes and fold-conditioning.

includes just hotspot information and another that includes fold–conditioning information and hotspot information. The updated feature shapes and definitions of the entries in each dimension for each model are provided in Supplementary Methods Table 7, respectively.

4.3.2 Secondary structure and block adjacency

The idea to use secondary structure and block-adjacency information was first introduced by Anand and Achim [8]. We review the idea here, discuss the motivation behind the idea, and describe our implementation in depth.

Often, a protein designer will desire a protein with a specific fold (for example: a transmembrane pore made of a beta barrel or a protein binder made of a three-helix bundle). A protein fold is defined by (1) the secondary structure blocks (contiguous regions of alpha helix, beta strand, or loop) it contains and (2) the exact orientation (translation and rotation) of these blocks with respect to one another. A protein designer will often desire to generate diversity within a specific type of fold as well. In such cases, it is critical to allow the under-specification of the exact fold to

allow for diversity within the generated structures; we call these broad collections of folds of the same type a “fold family”.

We wish to train a model which can be conditioned on a fold family. To allow the under-specification of a fold we choose to provide the model coarse information on which secondary structure blocks are within a distance cutoff of one another. Specifically, we provide the following features to the model: (1) an [L,4] one-hot tensor where each position is assigned to a secondary structure type {helix, sheet, loop, mask} and (2) an [L,L,3] one-hot tensor (called the block adjacency matrix) where entries indicate membership in blocks that are within a distance cutoff of one another {non-adjacent, adjacent, mask} (as in Extended Data Fig. 4A).

Secondary structure annotations of every residue in the training set were calculated using DSSP [75]. DSSP is a structure-based algorithm that assigns a per-residue classification of secondary structure type. The block-adjacency matrix of every structure in the training set was calculated from the secondary structure string returned by DSSP. Blocks are marked as “adjacent” in the block-adjacency matrix if (1) neither block is of loop type and (2) the minimum C_α–C_α distance of any pair of inter-block residues is within 8Å.

The 1-D secondary structure tensor is concatenated to RF’s 1-D template feature (`t1d`, see Supplementary Methods Table 1). The 2-D block-adjacency matrix is concatenated to RF’s 2-D template feature (`t2d`, see Supplementary Methods Table 1). The updated feature shapes and the definitions of the entries in each dimension are provided in Supplementary Methods Tables 7 and 8.

4.3.3 Fine-tuning for protein–protein interaction design

The version of RFdiffusion fine-tuned on protein complexes, is trained starting from the base version of RFdiffusion trained for 5 epochs. The training task consists of monomer examples (50%) and complex examples (50%). When the model is shown a complex example, only one side of the complex is noised, the other side is kept fixed (this is in keeping with established PPI design methods [26] where the target protein is kept fixed). When the model is shown a

complex example the model is provided with the residue indices of 0–20% of the residues ("hotspot residues") in the interface on the fixed chain side (the interface is defined as all residues within 10Å C_β–C_β distance of another chain), to permit targeting of the designed binder at inference time. In a separate model, also trained on protein complexes, during both complex and monomer training the model is provided with secondary structure 50% of the time and (independently) block-adjacency information 50% of the time for the noised region. The junctions between blocks of secondary structure and their corresponding entries in the block-adjacency matrix are masked during training, such that at inference time, one does not need to specify exact, per residue secondary structure and block-adjacency matrices. Specifically, 0–75% of secondary structure (and corresponding adjacency, when provided) is masked, with this masking occurring over junctions in secondary structure (mask length 1–8 residues). In totality, this training regimen permits the provision of partial secondary structure and/or adjacency information at inference time.

4.4 Use of potentials for symmetric oligomers and pocket design

Section 3.4 describes our approach to guiding the reverse diffusion process with potentials. We now describe the details of our choices of $P(x^{(t)})$ in applications to symmetric oligomer design and design of enzymes with concave pockets. When designing symmetric oligomers, we employ an inter-chain and intra-chain contact potential to promote the formation of contacts between subunits. Letting $Z = [z^1, \ldots, z^K]$ denote the C_α coordinates in oligomer with K subunits and L residues in each subunit (so for each k, $z_k = [z_{k,1}, \ldots, z_{k,L}]$ with each $z_{k,l} \in \mathbb{R}^3$) we set

$$P_{\mathrm{sym}}(Z) = \sum_{1\le k,k',\le L} \sum_{1\le l,l'\le L} (\mathbb{1}[k \neq k']w_{\mathrm{inter}} + \mathbb{1}[k = k']w_{\mathrm{intra}})\mathrm{Switch}(\|z_{k,l} - z_{k',l'}\|_2^2),$$

where w_{inter} and w_{intra} weight the inter-chain and intra-chain potentials, respectively. We set $w_{\mathrm{inter}} = 2$ and $w_{\mathrm{intra}} = 0.2$ to prioritize the formation of inter-subunit contacts while encouraging individual subunits to be well–packed.

$\text{Switch}(r) = \frac{1-(\frac{r-d_0}{r_c})^n}{1-(\frac{r-d_0}{r_c})^m}$, is a switching function which smoothly transitions from 1 if two atoms are within contact range to 0 when they are out of range. We set the hyperparameters that control its functional dependence on distance as $n = 6$, $m = 12$, $d_0 = 8$, and $r_c = 4$, to reflect the contact distances we would expect between interacting sidechains. It is sufficient to predominantly bias only the early sampling steps ($t \approx T$) to promote contacts in the higher order structure, and unnecessary to continue to do so at towards the end of design trajectories, by which point the quaternary structure is sufficiently determined. As such, we scale the potential by a "guide-scale", $g(t)$, as

$$P_{\text{sym}'}(Z, t) = g(t) P_{\text{sym}}(Z),$$

for $g(t) = (\frac{t}{T})^2$.

When designing enzymes, in addition to recapitulating the sidechain geometry of the active site, a pocket must be formed which has shape complementarity to the substrate. This condition can be captured effectively by a simple attractive-repulsive potential parameterized by the minimum distance between enzyme C_α carbons and substrate atoms. Denoting the coordinates of a substrate with K atoms by $s = \{s_k\}_{k=1}^K$ and the C_α coordinates by $z = [z_1, \ldots, z_L]$, we set: $P_{\text{enzyme}}(z, s) = w_{\text{attr}}[\sum_{1 \le l \le L} \text{Switch}(\min_{1 \le k \le K} \|z_l - s_k\|_2^2) - w_{\text{rep}}[\sum_{1 \le l \le L} \text{Rep}(\min_{1 \le k \le K} \|z_l - s_k\|_2^2)]$, where $\text{Rep}(r) = \mathbb{I}\{r < r_0\} \frac{|r_0 - r|^p}{p r_0^{(p-1)}}$, and we set $w_{\text{attr}} = 1, w_{\text{rep}} = 4, r_0 = 2, p = 1.5$.

The gradient of $\text{Rep}(r)$ decays smoothly from -1 at $r = 0$ to 0 at $r = r_0$, penalizing clashes between the protein backbone and the substrate. We do not use a guide scale with P_{enzyme}, as the potential relates to fine-grained details of the structure which are not fully determined until late in the reverse diffusion process. Empirically, we find the model is sufficiently receptive and robust to bespoke potentials with hyperparameters chosen based on physical intuition. We find that were able to achieve our objectives of interface production in the case of symmetric oligomer design, and implicit substrate modeling in the case of enzyme design without exhaustive hyperparameter tuning.

5 In silico experimental methods

5.1 Justification for using AlphaFold2 as an *in silico* metric

Throughout this work, we generally rely on AF2 for *in silico* validation of designs. A potential concern with the use of a structure prediction network, such as AF2, for validation of the accuracy of RFdiffusion (which is fine-tuned from the RF structure prediction network) is the risk of adversarial examples, given the architectural and training similarities of AF2 and RF. This has been discussed previously [4], and we revisit this concern in this section.

AF2 and RF share notable architectural similarities, and RF is also trained on a distillation set of AF2-predicted structures. ESMFold [21] also shares such similarities (it is similarly trained on AF2-predicted structures). Therefore, these networks are only partially-orthogonal means of validating designed sequences, when one of these structure prediction networks (or a fine-tuned variant of it) is used for protein design. Conveniently, while designing proteins with a structure prediction network and validating designs with the same network has been demonstrated not to work [7, 76], significant literature now demonstrates that using a different structure prediction network, after sequence re-design with, for example, ProteinMPNN [1], provides sufficient orthogonality for predictions to be indicative of experimental success [4, 7, 77, 56, 78, 79, 80, 54].

5.2 ProteinMPNN and AlphaFold2 settings

The precise settings in which ProteinMPNN and AF2 were used differs slightly for the different benchmarks and design campaigns in the paper, and these settings derive from prior established work. These modifications are described in full here.

ProteinMPNN: For protein binder design (Fig. 6), ProteinMPNN is used with a non-default very low sampling temperature, 0.0001. We additionally tested using ProteinMPNN, Rosetta FastRelax [58], and a second round of ProteinMPNN. These settings follow current best practice

[26]. In all other cases, ProteinMPNN was used with default parameters (sampling temperature = 0.1). Cysteines were generally omitted during sequence design, to help with expression and solubility.

AlphaFold2: Throughout the manuscript, AF2 is used in single-sequence mode (no multiple sequence alignment is provided).

For unconditional design, fold-conditioned design and functional motif scaffolding, we use AF2 model_4_ptm, without templates (following previous work [4]).

For validation of symmetric oligomers (cyclic and dihedral; the symmetries for which we report in silico success, Extended Data Fig. 5B), we use AF2 model_4_ptm to predict the whole complex (with n chains, each separated by a 200 amino acid residue offset). AF2 coordinates were initalized at the design structure (the so called "initial guess" method), following previous work designing symmetric oligomers [7]. For icosahedral and octahedral designs, the same pipeline was followed, except that only the C3 symmetric unit was validated, for computational tractability. For tetrahedral designs, we validated only on the monomers.

For validation of nickel-binding oligomers, the validation was the same as for other symmetric oligomers, except that AF2 model_5_ptm was used.

For validation of protein binders, we 1) template the (sometimes cropped) target protein and use AF2 model_1_ptm, and use 2) the "initial guess" method. These settings exactly mirror current best practice, where they have been shown to predict experimental success [26].

ESMFold: For unconditional and fold-conditioned monomer designs in the manuscript, we further validated designs using ESMFold [21]. ESMFold was run using default parameters.

5.3 *In silico* success rate

For all monomer-design tasks we define *in silico* success as backbone RMSD AF2 vs RFdiffusion < 2Å and Motif backbone (when present) RMSD AF2 vs native < 1Å, AF2 pAE < 5; these

choices are largely in line with previous work. They were chosen because they have been shown to be stringent filters indicative of experimental success (Wang et al., 2022 [4]). The only change with respect to Wang et al., 2022 is to use pAE < 5 rather than plDDT > 80 as the AF2 confidence metric. pAE is more stringent, and was chosen to prevent edge cases with high plDDT (e.g. single long helices), that are unlikely to behave well as real proteins. For other tasks, there are additional measures of success. These are all grounded in theory and/or data from prior work:

Binder design: In line with current best practice [26] we additionally filter on an inter-chain AF2 pAE of < 10. We also require binders to be within 1Å of the design structure, and use plDDT as the confidence metric, in line with [26].

Enzyme active site scaffolding: We additionally filter on sidechain RMSD between the AF2 prediction and the design model < 1.5Å. This is because sidechain placement is crucial for enzyme activity.

Fold-conditioned design: We additionally filtered on a TM score between the design and the desired fold of > 0.5. A TM score of > 0.5 is used to define "similar" folds [81], so we ensure that the design is of that specific fold.

Symmetric oligomer design: For symmetric oligomer design, we follow prior work [7] and use plDDT > 80 as the AF2 confidence metric indicating *in silico* success. For the nickel-binding oligomers, where the placement of the imidazole group of the histidine is important for nickel binding, we additionally filter on sidechain RMSD < 1.5Å, in line with the enzyme active site scaffolding metrics.

5.4 Unconditional benchmarking

To test RFdiffusion on unconditional generation of monomers (Fig. 2B-E), we generated 100 designs for lengths 100, 200, 300, 400, 600, 800 and 1000 amino acids. For each backbone, we generated 8 sequences with ProteinMPNN and subsequently predicted their structures with AF2 (or ESMFold

- Fig. Extended Data Fig. 1B, Extended Data Fig. 4D, Supplementary Information Fig. 2B). The best sequence (by alignment of the predicted structure to the design model) was taken for each backbone. We benchmarked against the recently-published RoseTTAFold Hallucination [6]. As some knowledge of how best to use RoseTTAFold for Hallucination is required, these samples were generated by the respective expert. ProteinMPNN was used to design sequences for all benchmarking designs. For ProteinMPNN, a sampling temperature of 0.1 was used, and cysteines were omitted from the designs (as these are often problematic for protein purification). Fourteen 300 amino acid proteins and four 200 amino proteins generated with RFdiffusion were ordered and tested experimentally for expression and CD profiles.

5.5 Conditional benchmarking

The full conditional benchmark is described in Supplementary Methods Supplementary Methods Table 9, and encompasses 25 design challenges from six recent publications [4, 5, 29, 38, 39, 40]. RFdiffusion was compared to RoseTTAFold Hallucination and RF_{joint} Inpainting. While both Hallucination and Inpainting are able to generate sequences directly, for the fairest comparison, we also redesigned the sequence with ProteinMPNN, and took the best of 8 sequences per backbone. Both RF_{joint} Inpainting and RF Hallucination are able to scaffold structure without sequence, so in cases where functional-site residues were not required for function, these methods were permitted to redesign the sequence of the non-functional residues, which is generally beneficial for design. Finally, as Hallucination requires some expert knowledge and empirical hyperparameter tuning, some exploration of the benchmark set was permitted, and these designs were generated by the respective expert.

For a number of comparisons made in the paper (Extended Data Fig. 1, Extended Data Fig. 2B-C, Supplementary Information Fig. 1, Supplementary Information Fig. 2D), a smaller benchmark encompassing a subset of unconditional and conditional benchmark problems described

above was used.

Name [Ref.]	Description	Input	Total Length	Sequence to be redesigned*
1PRW[4]	Double EF-hand motif	5-20,A16-35,10-25,A52-71,5-20	60-105	A16-19,A21,A23,A25, A27-30,A32-35,A52-55, A57,A59,A61,A63-66,A68-71
1BCF[4]	Di-iron binding motif	8-15,A92-99,16-30,A123-130,16-30,A47-54,16-30,A18-25,8-15	96-152	A19-25,A47-50,A52-53,A92-93,A95-99,A123-126,A128-129
5TPN[4]	RSV F-protein Site V	10-40,A163-181,10-40	50-75	A163-168,A170-171,A179
5IUS[4]	PD-L1 binding interface on PD-1	0-30,A119-140,15-40,A63-82, 0-30	57-142	A63,A65,A67,A69,A71,A72,A76, A79,A80,A82,A119,A120,A121, A122,A123,A125,A127,A129, A130,A131,A133,A135,A137, A138,A140
3IXT[39]	RSV F-protein Site II	10-40,P254-277,10-40	50-75	P255,P258-259,P262-263, P268,P271-272,P275-276
5YUI[4]	Carbonic anhydrase active site	5-30,A93-97,5-20,A118-120,10-35,A198-200,10-30	50-100	A93,A95,A97,A118,A120
1QJG[4]	Delta5-3-ketosteroid isomerase active site	10-20,A38,15-30,A14,15-30,A99,10-20	53-103	n/a
1YCR[4]	P53 helix that binds to Mdm2	10-40,B19-27,10-40	40-100	B20-22,B24-25
2KL8[4, 29]	De novo designed protein	A1-7,20,A28-79	79	n/a
7MRX_60[29]	Barnase ribonuclease inhibitor	0-38,B25-46,0-38	60	n/a
7MRX_85[29]	Barnase ribonuclease inhibitor	0-63,B25-46,0-63	85	n/a
7MRX_128[29]	Barnase ribonuclease inhibitor	0-122,B25-46,0-122	128	n/a
4JHW[38]	RSV F-protein Site 0	10-25,F196-212,15-30,F63-69, 10-25	60-90	F196,F198,F203,F211-212,F63,F69
4ZYP[38]	RSV F-protein Site 4	10-40,A422-436,10-40	30-50	A422-427,A430-431,A433-436
5WN9[39]	RSV G-protein 2D10 site	10-40,A170-189,10-40	35-50	A170-175,A188-189
6VW1[4, 40]	ACE2 interface binding SARS-CoV-2	E400-510/20-30,A24-42,4-10, A64-82,0-5†	62-83	A25-26,A29-30,A32-34,A36-42,A64-82
5TRV_short[5]	De novo designed protein	0-35,A45-65,0-35	56	n/a
5TRV_med[5]	De novo designed protein	0-65,A45-65,0-65	86	n/a
5TRV_long[5]	De novo designed protein	0-95,A45-65,0-95	116	n/a
6E6R_short[5]	Ferridoxin Protein	0-35,A23-35,0-35	48	n/a
6E6R_med[5]	Ferridoxin Protein	0-65,A23-35,0-65	78	n/a
6E6R_long[5]	Ferridoxin Protein	0-95,A23-35,0-95	108	n/a
6EXZ_short[5]	RNA export factor	0-35,A28-42,0-35	50	n/a
6EXZ_med[5]	RNA export factor	0-65,A28-42,0-65	80	n/a
6EXZ_long[5]	RNA export factor	0-95,A28-42,0-95	110	n/a

Supplementary Methods Table 9: **A benchmarking set of recently published functional-motif scaffolding problems.** To benchmark RFdiffusion at functional-site scaffolding, against existing methods, we generated a benchmark set encompassing problems described in six recent publications [4, 5, 29, 38, 39, 40], which utilize a range of design methodologies to address these problems. For each problem, named by PDB accession (and, where applicable, the length of the designs to be generated, left column), we recapitulated the inputs as closely as possible with respect to details available in each publication. So that others can test methods on this benchmark, the exact input is specified in the third column. In bold, prefixed by a letter, are the inputs (chain, residues) from the PDB structure provided to the model (the "functional-site"). In non-bold text are the lengths that the different methods randomly sampled to generate good designs. The final lengths of the proteins were either specified by the input to the model, or were provided as constraints (for example, for "6EXZ_Long", the model could sample any N- and C-terminal length between 0 and 95 residues, but the total length of the output had to equal 110 amino acids). For each design challenge, 100 designs were generated, and ProteinMPNN was used to design the sequence of the designed scaffold (the motif sequence was fixed). 8 sequences were designed, with the best sequence chosen for each backbone. *Both the RF_{joint} and RoseTTAFold constrained hallucination approaches can simultaneously redesign sequences during generation, which can, in some cases, be helpful (if extracting the motif exposes hydrophobic residues which may subsequently end up as surface residues in the output designs, for example). Therefore, in this benchmark, these methods were allowed to redesign non-functional residues, listed in the right-most column. † This example is multi-chain generation (scaffolding a functional-site in the presence of a second chain). All methods benchmarked here can represent chain breaks (with large residue index jumps). Full results are shown in Fig. 4A, and tabulated in Supplementary Methods Table 10.

Additionally, to test whether the ability of RFdiffusion to scaffold functional sites was related to their presence in the RF or RFdiffusion training set (Supplementary Information Fig. 7), we compiled a benchmark of proteins either 1) in the RF and RFdiffusion training set, or 2) from orphan proteins not present in the RF, AF2 or RFdiffusion training sets, without known homologs in nature. The 15 orphan proteins are those listed in Fig. 2 of [20]. The 15 proteins from the training set were randomly sampled. To construct a benchmark set, we randomly sampled "motifs" from these 30 structures. We sampled both "single" and "double" motifs. For "single" motifs, we randomly sampled a contiguous 15 amino acid stretch from the structures. For the "double" motifs, we sampled two motifs, each internally contiguous, separated by at least 20 amino acids in primary sequence, but close in Euclidean space. Motif scaffolding was then run with a consistent number of residues, chosen without prior visualization of the motif structures. This is detailed, along with success rates, in Supplementary Methods Table 11.

**Problem Name**	**RFdiffusion (noise=0)**	**RFdiffusion (noise=1)**	**RFjoint**	**RFjoint + Protein-MPNN**	**RF Hallucination**	**RF Hallucination + Protein-MPNN**
1BCF	**100**	98	65	**100**	0	0
6E6R_med	**89**	67	0	27	2	9
2KL8	88	**96**	71	95	20	34
6E6R_long	**86**	63	0	4	0	1
6EXZ_long	**76**	51	0	0	1	4
1YCR	**74**	58	12	57	11	61
6VW1	**69**	66	0	24	2	32
5TPN	**61**	59	0	3	0	1
6EXZ_med	**49**	33	0	3	5	15
4ZYP	**40**	31	1	21	1	18
6E6R_short	**39**	29	0	23	3	7
5TRV_long	**37**	30	0	0	0	2
3IXT	35	16	21	**62**	2	34
5TRV_med	**24**	20	0	3	0	3
7MRX_85	**11**	6	0	0	0	0
7MRX_128	**9**	4	0	0	0	0
1PRW	8	9	0	**22**	0	0
5TRV_short	4	**7**	0	2	0	1
7MRX_60	**2**	0	0	0	0	0
6EXZ_short	2	4	1	**27**	4	15
5IUS	**2**	0	0	1	0	0
5YUI	0	0	0	**1**	0	0
5WN9	0	**1**	0	0	0	0
4JHW	0	0	0	0	0	0
1QJG	0	**2**	0	0	0	0

Supplementary Methods Table 10: **Functional-site scaffolding benchmark results.** Full results for the benchmark test described in Fig. 4A and Supplementary Methods Table 9. In each case, values represent the success rate (%) in a set of 100 designs generated with each method.

PDB	Set	Input, Single Motif	Total Length, Single Motif	Success Rate (%)	Input, Double Motif	Total Length, Double Motif	Success Rate (%)
7F7P	Orphan	0-100,B7-21,0-100	115	64	0-60,B32-46,30-60,A7-21,0-60	140	1
7AD5	Orphan	0-100,A99-113,0-100	115	0	0-60,A89-103,30-60,A37-51,0-60	140	0
7MQQ	Orphan	0-100,A115-129,0-100	115	42	0-60,A115-129,30-60,A80-94,0-60	140	1
7DGW	Orphan	0-100,A30-44,0-100	115	97	0-60,A70-84,30-60,A22-36,0-60	140	3
7KWW	Orphan	0-100,B14-28,0-100	115	5	0-60,B38-52,30-60,B10-24,0-60	140	0
7AHO	Orphan	0-100,A199-213,0-100	115	18	0-60,E119-133,30-60,E216-230,0-60	140	0
7WRK	Orphan	0-100,A80-94,0-100	115	0	0-60,A99-113,30-60,A132-146,0-60	140	0
7TJL	Orphan	0-100,A32-46,0-100	115	67	0-60,A67-81,30-60,A31-45,0-60	140	54
7A8S	Orphan	0-100,A14-28,0-100	115	83	0-60,A41-55,30-60,A72-86,0-60	140	2
7KUW	Orphan	0-100,A38-52,0-100	115	85	0-60,A30-44,30-60,A2-16,0-60	140	26
7BNY	Orphan	0-100,A85-99,0-100	115	0	0-60,A83-97,30-60,A111-125,0-60	140	0
7S5L	Orphan	0-100,A365-379,0-100	115	58	0-60,A27-41,30-60,A77-91,0-60	140	32
7CG5	Orphan	0-100,A95-109,0-100	115	0	0-60,A6-20,30-60,A63-77,0-60	140	21
7DNS	Orphan	0-100,A58-72,0-100	115	81	0-60,B3-17,30-60,B48-62,0-60	140	52
7K3H	Orphan	0-100,B47-61,0-100	115	83	0-60,A5-19,30-60,A55-69,0-60	140	84
4JWC	Train	0-100,B583-597,0-100	115	44	0-60,B459-473,30-60,B424-438,0-60	140	0
4WSF	Train	0-100,A44-58,0-100	115	24	0-60,A53-67,30-60,A23-37,0-60	140	0
3ES3	Train	0-100,A143-157,0-100	115	85	0-60,A56-70,30-60,A17-31,0-60	140	55
3FKA	Train	0-100,D103-117,0-100	115	54	0-60,C22-36,30-60,C103-117,0-60	140	38
2W7Y	Train	0-100,A141-155,0-100	115	1	0-60,A290-304,30-60,A374-388,0-60	140	0
5ECF	Train	0-100,D62-76,0-100	115	74	0-60,H125-139,30-60,F119-133,0-60	140	4
4M1T	Train	0-100,A111-125,0-100	115	0	0-60,C121-135,30-60,C83-97,0-60	140	0
6FFW	Train	0-100,B99-113,0-100	115	0	0-60,A183-197,30-60,A212-226,0-60	140	73
1YES	Train	0-100,A38-52,0-100	115	12	0-60,A75-89,30-60,A209-223,0-60	140	3
5NE0	Train	0-100,A63-77,0-100	115	1	0-60,A11-25,30-60,A89-103,0-60	140	58
2FYD	Train	0-100,D297-311,0-100	115	17	0-60,D150-164,30-60,D382-396,0-60	140	8
5JKB	Train	0-100,D33-47,0-100	115	0	0-60,A131-145,30-60,A79-93,0-60	140	31
3TQB	Train	0-100,A57-71,0-100	115	4	0-60,A65-79,30-60,A37-51,0-60	140	13
2EF5	Train	0-100,F208-222,0-100	115	18	0-60,D140-154,30-60,D94-108,0-60	140	0
4XJC	Train	0-100,F109-123,0-100	115	11	0-60,D52-66,30-60,D160-174,0-60	140	0

Supplementary Methods Table 11: **Scaffolding sites from orphan proteins or proteins in the training dataset.** Full description of the protein motifs and lengths used to compare RFdiffusion performance at scaffolding motifs from the training set and motifs from orphan proteins with no homology to proteins in the training set. The single 15 residue "motif" was randomly selected. The double motifs were randomly selected as being separated (> 20 amino acids) in primary sequence, but close in Euclidean space. The length ranges used to scaffold these motifs were the same for all motifs. *In silico* success rates are also reported.

5.6 Assessing diversity of designs

Designs were assessed for their structural diversity both to each other, and to the PDB (PDB100 April 19, 2022), using the TM score [82]. Full results (encompassing 5th, 25th 50th 75th and 95th percentiles) for all design campaigns shown in the paper are detailed in Extended Data 1. For motif scaffolding cases, the motif region was extracted from the structure that was TM aligned, to prevent undue bias from the (native) motif within the designed structures. In Extended Data Fig. 1I and Extended Data Fig. B-C, designs were clustered at a 0.6 pairwise TM score cutoff.

5.7 Using protein BLAST to check for similar sequences in UniRef90

In Extended Data 1, we provide statistics on protein BLAST hits from the 2022-04-25 version of UniRef90 for designed sequences from each problem attempted in the paper. Here, we outline the methods used to procure those results.

For each problem, a fasta format file was created containing the sequences designed by ProteinMPNN for RFdiffusion backbones. The fastas were split, and then commands were created to run Protein-Protein BLAST version 2.11.0+ against the UniRef90 database, an example of which is shown below (note back slashes are denoting new lines in a shell script):

```
blastp -query example_fasta.fasta\
-db /path/to/databases/uniref90\
-evalue 1e-1\
-num_threads $SLURM_JOB_CPUS_PER_NODE > output.fasta
```

This execution meant that only hits with e-value below 1e-1 (a generous cutoff) were reported by BLAST into the output file. The output files were then parsed into a pandas DataFrame, and hits per design were de-duplicated to keep the strongest hit (lowest e-value) if a hit existed for a design. Statistics could then be reported on (a) the fraction of designs for a problem that had BLAST

hits below e-value 1e-1 and (b) the 5th, 25th, 50th, 75th, and 95th percentile sequence identity fractions outside of the motif (if applicable) for the most significant BLAST hit, normalized to the number of query/design positions. In other words, when calculating the fraction of identities normalized to the query sequence, amino acids in the query and the target that were aligned and identical but were part of the motif did not contribute towards the sequence ID fractions. These data are reported in Extended Data 1.

5.8 Assessing choice of losses

Previous work on using DDPMs for protein design has used Frame Aligned Point Error (FAPE) as the loss function [8]. FAPE was introduced in AF2 and was also used to train RoseTTAFold. FAPE is SE(3) invariant but not invariant to reflections, this makes it an ideal loss for protein structure prediction where the exact global orientation of the predicted structure is arbitrary, but chirality within the structure is important. With a DDPM, however, $x^{(0)}$ must be in the same global frame as $x^{(t)}$ since $x^{(0)}$ and $x^{(t)}$ are interpolated between to generate $x^{(t-1)}$. We reasoned that, as FAPE is SE(3) invariant, a model trained with FAPE would not learn to make predictions in the same global frame as the inputs. We tested this by comparing a model trained with FAPE to a model trained with the C_α and rotation squared distance losses described in Section 1.4. By contrast these losses are not SE(3) invariant.

We found that the model trained with FAPE was quite poor at unconditional generation (Extended Data Fig. 1D, left). In the motif scaffolding task, $x^{(0)}$ and $x^{(t)}$ can be aligned to one another using the fixed motif. This effectively eliminates the global frame problem as any arbitrary SE(3) action applied by the model can be reversed by this motif-alignment step. In the motif-scaffolding task we found that the model trained with FAPE performed comparatively to the model trained with MSE losses (Extended Data Fig. 1D, right). We conclude that maintaining a global coordinate frame is vitally important for coherence of RFdiffusion trajectories. We further conclude that, while the

squared distance nature of the MSE loss promotes matching the reversal of the forward process (Section 2.2, 2.3, 2.5), the L1 FAPE loss, when a global coordinate system is available (through alignment to a fixed motif), empirically performs equivalently.

5.9 Design of fold-conditioned proteins

To design TIM barrels, we constructed secondary structure and block adjacency inputs from a previously-designed TIM barrel (PDB: 6WVS). Any regions of loop secondary structure were masked, and to generate larger TIM barrels than the original, we randomly sampled additional length (1-15 residues inserted as “mask” tokens into the loops). We generated a total of 2400 designs, generating designs with three different noise scales during inference (0, 0.5, 1). No external potentials were used. Designs were classified as TIM barrels if the TM score against 6WVS was greater than 0.5, and AF2 repredicted the designs (pAE < 5, RMSD to design < 2Å). 11 designs passing stringent filters (AF2 pAE < 3.5, RMSD AF2 vs design < 0.75Å) were ordered.

To design NTF2 folds, we constructed secondary structure and block adjacency inputs from a preexisting set of 1000 NTF2 proteins. These were randomly selected and used as input to RFdiffusion. 900 designs were generated in total, at three different noise scales (0, 0.5, 1). Designs were classified as NTF2 folds if they had a TM score greater than 0.5 to PDB: 1GYS.

5.10 Design of symmetric oligomers

To better understand RFdiffusion's capacity to design symmetric oligomers, we generated backbones for the following groups: dihedral (D2, D3, D4, D5), cyclic (C3, C5, C6, C8, C10, C12), tetrahedral, octahedral, and icosahedral. We tested RFdiffusion's ability to design symmetry for these groups both with and without a guiding potential function for inter- and intra- chain contacts, weighting in all cases the intrachain contacts over the interchain. For dihedral, cyclic, and tetrahedral symmetries, protomers had 60-110 amino acids per chain, and for a subset of the cyclic

symmetries (C3, C5, C6), additional models were designed with large protomers (150-400 amino acids per chain) to test RFdiffusion's ability to design unconditional yet large oligomers. The octahedral and icosahedral models were designed by modeling the minimal number of subunits (100-200 amino acids per protomer) required to capture all axes of symmetry (O: 4-, 3-, and 2-fold; I: 5-, 3-, and 2-fold).

Original backbones were filtered by sufficient oligomeric interfaces (determined by C_α-C_α backbone distances between chains) to enrich for backbones with a higher likelihood for assembly following design. Cyclic and D2 symmetries were filtered for backbones consisting of protomers forming at least two distinct 10 residue interfaces, whereas all other symmetries were required to form at least three distinct 10 residue interfaces. Following filtering, all backbones were redesigned with ProteinMPNN, and then sequences were validated by AF2 (for the cyclic and dihedral symmetries). Given the complexity and challenge these symmetries present, we provided AF2 with an initial guess, as done in Bennett et al. [26], and increased the number of recycles the model could use in the predictions. Tetrahedra were predicted using RoseTTAFold, and octahedron and icosahedron were predicted with AF2 along their C3 axes of symmetry only. Designs were considered successful (success rates for cyclic and dihedral shown in Extended Data Fig. 5B) if the structure predictions had a mean plDDT $>$ 80 and an RMSD between prediction and design model of $<$ 2Å. This same filtering regime was also used for the cage symmetries, but applied to the C3 predictions (for octahedra and icosahedra), and the monomer predictions (for tetrahedra).

5.11 Design of p53 helix scaffolds

To design scaffolds able to hold the Mdm2-binding helix of p53, we used the version of RFdiffusion fine-tuned for protein-protein interaction design (Section 4.2), and provided the network with both the p53 helix and the whole Mdm2 protein structure from PDB: 1YCR. To encourage extra contacts with the target protein, we explored using an external potential to encourage inter-chain contacts

(Section 4.4). The set of 96 designs were filtered by RMSD between the AF2 model and design < 1.2Å, AF2 pAE < 6.6, AF2 pAE between the two chains < 10, and a radius of gyration of the monomer < 16 Å. 45/96 designs were generated without external potentials, and 51 with potentials. No fold information was provided to the network in this design case.

5.12 Design of theoretical scaffolds to enzyme active sites

To design scaffolds able to hold the catalytic sites of enzymes, we used the version of RFdiffusion fine-tuned for sparse motif masks. Curated enzyme active site annotations were obtained from M-CSA. There are 7 enzyme classes in M-CSA, but enzyme classes 1-5 comprise 96% of curated M-CSA entries with annotated residues, cofactors, reactants and products. For each enzymes class a random M-CSA ID corresponding to a triadic active site was selected. Multiple structures exist for any given M-CSA ID, so for each M-CSA ID structures containing that active site PDBs were pulled from at random from RCSB. A PDB was accepted for the category if the active site residues were at least 10 residues apart in order to fully capture the difficulty of catalytic site scaffolding. The selected (PDB ID, active site) for enzyme classes 1-5 were: (1a4i, Lys56-Gln100-Asp125), (1cwy, Asp293-Glu340-Asp395), (1de3, His50-Glu96-His137), (1p1x, Asp102-Lys167-Lys201), (1snz, His107-His176-Glu307). For each active site, 100 designs of length 150 with 10-100 residues spacing the active site residues were made for each of the six permutations of the active site residue orderings. 8 ProteinMPNN sequences per design were computed, and AF2 was used to predict structure of the design. Designs were considered successful (success rates for enzyme active site scaffolding shown in Fig. 4) if AF2 Motif RMSD vs native: backbone < 1 Å, backbone and sidechain atoms < 1.5 Å, RMSD AF2 vs design < 2 Å, AF2 pAE < 5.

5.13 Design of theoretical C3-symmetric spike SARS-CoV-2 spike protein binding oligomers

To design the theoretical C3-symmetric oligomers to scaffold the ACE2 mimic AHB2, we started with the C3-symmetric cryo-EM structure of the minibinders against the spike protein from [43]. The first 55 residues of the minibinder were used as the asymmetric unit in a C3-symmetric motif input to the model.

The model weights from both the 5th epoch of RFdiffusion training as well as the 8th epoch were used with T=200 length trajectories. All combinations of inter- and intrachain contact potentials with weights (0.1, 0.3, 0.5, 1) and (0.5, 1), respectively, were applied to the trajectories, with 25 designs being computed per combination. 32 ProteinMPNN sequences per design were computed, and AF2 was used to predict the structure of the oligomers via the inference method described by Wicky et al. [7].

5.14 Design of symmetric Nickel binding oligomers

To design the C4-symmetric Ni^{2+}-binding proteins, we started from three sets of imidazole groups positioned in square planar coordination geometry bearing C4 rotational symmetry with the associated symmetry axis being aligned to the Z-axis (Fig 5B, Supplementary Information Fig. 9). The imidazoles were placed with the NE2 atoms at a distance of 2.2Å from the metal center (a common bond length for His–Ni^{2+} in the MetalPDB [83]) and the different sets of imidazole groups were positioned such that they formed dihedral angles of 0°, 22°, and 45° between the Z-axis and the plane of the heterocyclic system (Supplementary Information Fig. 9A). We note that larger dihedral angles resulted in clashing imidazole moieties and were therefore not considered in our designs.

Next, three sets of backbone dependent, non-clashing inverse rotamers [59] from the Dunbrack

rotamer library were sampled for pieces of ideal alpha-helix ($\phi = -40°, \psi = -60°$) containing the histidine rotamers in the middle, and an alanine residue on either side of the histidine (three residues total per asymmetric unit going into the model). For set 1, rotamers chosen were of probability 0.3502, 0.1207, 0.0647, 0.0474, 0.0469 (Supplementary Information Fig. 9B), for set 2 probability 0.0365, and for set 3 probabilities 0.3502, 0.0648, 0.0305, and 0.0131 (Supplementary Information Fig. 9E). Note that the differences between the sets is that set 1 is associated with scaffolding the imidazole groups with no shear (0° dihedral) while sets 2 and 3 are associated with scaffolding the imidazole groups with shear (22°, 45°).

After construction of the inverse rotamers, the imidazole groups from their histidines were aligned to the aforementioned square-planar imidazole groups, resulting in various C4-symmetric motifs that could then be input to the model. 100 reverse diffusion trajectories were run for the full T=200 steps for all symmetric motifs, with 50 residues designed on either side of the inverse rotamer helix chunks in each chain (total complex length 412 residues, coordinating histidine always at position 52 in each chain). As in Section 4.4, an intra-chain guiding potential was used during the trajectory with a weight of 1, an inter-chain guiding potential with a weight of .06, and a global multiplicative factor of 2. For set 1, half (50) of the designs per motif were designed such that the effect of the external potential decayed quadratically during the trajectory, while the other half having potentials decay cubicly, while for sets 2 and 3 only a quadratically decaying external potential was utilized. Importantly, multiple models from the training session which produced RFdiffusion were tested to see which checkpoint could scaffold the sites most accurately, and pilot experiments suggested the set of weights after the 8th epoch, rather than the 5th epoch (standard used for this paper) should be used.

Before sequence design with ProteinMPNN, RFdiffusion outputs were filtered to only allow designs for which the backbone RMSD from the model < 1Å RMSD from the true motif. This yielded 199 backbones for set 1 and 201 backbones for sets 2 and 3, and ProteinMPNN was then used to perform symmetric sequence design on all residues except the histidines (the original alanines in

the motif were also re-designed), with 16 sequences per backbone. AF2 was then used to predict the structure of all designed sequences.

To assemble a final set of 24 designs to order for testing from set 1, designs from the set were filtered with the following criteria: (1) full-atom RMSD on all 4 histidines between the AF2 prediction and the motif ≤ 0.6Å, (2) AF2 plDDT ≥ 90 (3) AF2 PAE ≤ 6. This filtering yielded 39 designs. From here, these 39 designs were clustered at a TM-score cutoff of 0.85 using a simple greedy clustering algorithm, yielding 20 representative backbones. 4 additional designs passing the RMSD, plDDT and PAE metrics (presumably with TM-score > 0.85 vs some of the aforementioned 20) were hand-picked to create a final 24 designs from set 1.

To assemble a final set of 24 designs to order for testing from sets 2 and 3, designs from these sets were filtered with the following criteria: (1) Full atom RMSD on all 4 histidines between the AF2 prediction and the motif ≤ 0.66Å, (2) AF2 plDDT ≥ 90, (3) AF2 PAE ≤ 6. This yielded exactly 24 designs without clustering by TM-score. Unlike designs from the first set, in some cases, multiple ProteinMPNN sequences were ordered for a single designed backbone. This set of 24 proteins therefore comprised 10 unique RFdiffusion-generated backbones, all with a TM score of < 0.8 to the other backbones in the set.

Mutant sequences for all 48 ordered designs were created by simply replacing the histidine at position 52 with alanine. 44 of the wild-type designs were successfully transformed into *E. coli*, which is what is reported on in the main text and the experimental methods.

5.15 Design of protein binders to rigid targets

To test the ability of RFdiffusion to design de novo binders to rigid targets, we designed binders to five targets: PD-L1 (PDB: 5O45), IL7 Receptor subtype Alpha α (PDB: 3DI3), Insulin Receptor (PDB: 4ZXB), TrkA Receptor (PDB: 1WWW) and Influenza Hemagglutinin (PDB: 5VLI). We generated designs both with and without fold conditioning, with the folds used derived from

scaffold sets typically used for Rosetta-based protein binder design [12]. In all cases, we targeted binders, using input "hotspot" residues, to a specific site on the target protein. The hotspots selected were as follows (chain and residue index from PDB):

PD-L1: A56, A115, A123

IL7 Receptor subtype Alpha: B58, B80, B139

Insulin Receptor: E64, E88, E96

TrkA Receptor: X294, X296, X333

Influenza Hemagglutinin: B521, B545, B552

In line with current best practice, we tried using the ProteinMPNN-FastRelax protocol described in Bennett et al. [26], this protocol starts with a round of ProteinMPNN and then cycles between FastRelax [58] and ProteinMPNN to attempt to iteratively improve the sequence and structure agreement. We did not find ProteinMPNN-FastRelax to be systematically helpful for design success rates, perhaps because RFdiffusion generates high quality backbones to start with and the FastRelax refinement is not needed.

For the five design cases, we generated several thousand designs. To filter designs we ran AF2 with an initial guess and target templating [26]. Briefly, this configuration of AF2 runs without a multiple sequence alignment and without template information for the de novo binder, which ensures that predictions are not biased towards examples which have sequence or structual homology to the PDB. This configuration uses the template feature in AF2 to provide the exact structure of the target protein, as we are designing with a rigid target and know the structure of the target *a priori*, we desire for AF2 to keep the target fixed and only predict the dock and structure of the de novo binder. Finally, this configuration of AF2 initializes the dock and structure of the de novo binder with the RFdiffusion design model of the dock and structure; this protocol is well-characterized retrospectively and prospectively as described in Bennett et al [26]. We classed a design as successful if it had AF2 pAE of interaction between

binder and target < 10 (this has been shown to be highly indicative of design success), as well as RMSD between the designed binder and the AF2 prediction < 1Å, and AF2 plDDT > 80. Success rates are reported in Fig. 6B, and were orders of magnitude higher than with traditional Rosetta binder design. Retrospective success rates, reported in Fig 6 legend, were calculated by filtering the previously ordered design library to only those designs which passed the AF2 interchain pAE cutoff which was used in the RFdiffusion binder campaign for each target. The previously ordered libaries were generated using the method of Fleishman *et al* [84] for Flu HA and the method of Cao *et al* [12] for the others. The interchain pAE cutoffs were:

PD-L1: interchain pAE < 5
IL7 Receptor subtype Alpha: interchain pAE < 8
Insulin Receptor: interchain pAE < 5
TrkA Receptor: interchain pAE < 6
Influenza Hemagglutinin: interchain pAE < 10
The Influenza Hemagglutinin design campaign performed with the Rosetta pipeline was small (88 designs) and none of these designs passed the interchain pAE filter so this analysis was not included in Fig 6.
Before ordering designs we also manually removed edge case examples where a binder has only two helices and did not form a well-packed protein core since these proteins were not likely to express in solution.

5.16 Figures and statistics shown in the paper

Protein structures depicted in this paper were rendered in PyMOL V2.5.0 [85], and graphs were plotted with Matplotlib V3.6.2 [86] and Seaborn V0.11.2 [87]. Note that for all boxplots displayed in the paper, for aesthetic reasons, outliers are not displayed. SEC data was analyzed using PyCORN

0.19, and BLI data was analyzed using ForteBio data analysis software. Appropriate statistical tests were performed using SciPy [88], as indicated in figure legends.

5.17 Ablations

Throughout the manuscript, we include ablations to core aspects of RFdiffusion to provide an understanding of the determinants of high sample quality. These ablations, along with the results, are summarized in Supplementary Methods Supplementary Methods Table 12.

Furthermore, during design with RFdiffusion, we often include additional conditioning inputs to the model, which are useful in specific design contexts. As a summary of the specific design scenarios when such inputs are useful, we further provide Supplementary Methods Supplementary Methods Table 13. The table includes both the use case, and the associated data demonstrating the additional feature achieves the desired outcome.

Variable	Description	Ablation conclusion	Figure(s)
Self-conditioning	Allowing the model to condition on $\hat{X}^0{}_t$ (its previous prediction of the final structure) to make the prediction of $\hat{X}^0{}_{t-1}$	Using self-conditioning is necessary	2F, S3E,G
Structure prediction pre-training	Training RFdiffusion starting from pre-trained RoseTTAFold structure prediction weights, or from randomly initialized weights	With a fixed compute budget of 5 training epochs, structure prediction pretraining is necessary	2F, S3F
RFdiffusion training	Whether to perform diffusion training at all (i.e., performing diffusion style sampling from the original structure prediction network)	Training the diffusion task is necessary	2F
MSE training loss (as opposed to FAPE)	When training, whether to use MSE loss on coordinate and frame orientation (default) or use FAPE	MSE loss is necessary for performance on unconditional sampling, but not strictly necessary for performance on motif scaffolding	2F, S3D

Supplementary Methods Table 12: Training ablations for RFdiffusion.

Variable	Description	Ablation conclusion	Figure(s)
Oligomer inter- and intra-chain contact potentials	Using an auxillary potential function to bias oligomer trajectories to have more inter- and/or intra-chain contacts	Significantly increases *in silico* success rate for oligomer design.	S10C
Ligand "pocket" potential	Using auxillary potential function to bias trajectories to contact but not clash with the ligand	Using the pocket potential reduces protein-substrate clashes, increases protein-substrate contacts, and is necessary in practice when scaffolding enzyme active sites.	S16B
Interface hotspot feature	Binary feature indicating whether or not a residue is part of the binding interface in a complex	The hotspot feature grants desirable control over binder location.	S19B
Secondary structure / block adjacency features	Coarse grained 1D and 2D features input to the model, encoding secondary structure type and secondary structure element proximity, respectively.	SS/Block adj. features allow efficient sampling of a fold family.	2H,S7,S17D

Supplementary Methods Table 13: Inference time ablations for RFdiffusion.

6 In vitro experimental methods

6.1 Plasmid construction

Symmetric oligomer, unconditional proteins, TIM barrels and protein binder designs were ordered as synthetic genes (eBlocks, Integrated DNA Technologies) with compatible BsaI overhangs to the target cloning vector, LM0627 (see Wicky et al. [7]) for Golden Gate assembly. LM0627 is a modified expression vector containing a Kanamycin resistance gene and a ccdb lethal gene between BsaI cut sites. Subcloning into LM0627 results in the following product: MSG-[**protein**]-GSGSHHWGSTHHHHHH, with the C-terminal SNAC [89] cleavage tag and 6XHis affinity tag respectively underlined. Helical peptide binders were ordered in a similar format, except for the addition of adaptors (GGGSGGGGSASHMRS, SSEISFCSEPPPSRRS) permitting cloning into the pETcon3 vector (as well as LM0627), to permit both purification in *E. coli* and yeast surface display.

6.2 Protein expression and purification

For the oligomeric, unconditional proteins, TIM barrels and protein binder expression screens, a previously reported protocol was followed [7], with some modifications as denoted. In short, Golden Gate subcloning reactions of designs were carried out in 96-well PCR plates in 1μL volume. Reaction mixtures were then transformed into a chemically competent expression strain (BL21(DE3)), and 1-hour outgrowths were split directly into four 96-deep well plates containing 0.9-1.0mL of auto-induction media (autoclaved TBII media supplemented with Kanamycin, 2mM MgSO4, 1X 5052) for a final total volume of approximately 4ml. The following day (20-24 hrs later), cells were harvested and lysed, and clarified lysates were applied directly to a 50μL bed of Ni-NTA agarose resin in a 96-well fritted plate equilibrated with a Tris wash buffer. After sample application and flow through, the resin was thoroughly washed, and samples were eluted in 200μL of a Tris elution

buffer containing 300mM imidazole. For oligomers, 0.5 M EDTA was spiked into the eluates (10 mM final) to reduce self-association due to the 6XHis tag. All eluates were sterile filtered with a 96-well 0.22μm filter plate (Agilent 203940-100) prior to size exclusion chromatography.

Protein designs were then screened via SEC using an AKTA FPLC outfitted with an autosampler capable of running samples from a 96-well source plate. The symmetric oligomers, unconditional proteins and TIM barrels were run on a Superdex200 Increase 5/150 GL column (Cytiva 28990945). The protein binders were run on a Superdex75 Increase 5/150 GL column (Cytiva 29148722). The icosahedral designs were run on a Superose6 5/150 GL column (Cytiva 29091597). For the cyclic and dihedral symmetric oligomers, and the unconditional proteins and TIM barrels, either a running buffer of 20 mM NaPhos pH 7.4, 100 mM NaCl or 20 mM Tris pH 8, 50 mM NaCl was used. For the tetrahedral, octahedral, and icosahedral oligomers, samples were run in 20 mM Tris pH 8, 50 mM NaCl, 100 mM Glycine. To improve peak resolution, the SEC column was connected directly in line from the autosampler to the UV detector. 0.25 mL fractions were collected from each run, and selected fractions were pooled for further analysis (LC-MS, native mass spectrometry, negative stain EM, SDS-page).

Genes encoding the designs for Ni2+-binding were cloned into a C-terminal Strep-tag construct via the Golden Gate method. Resulting plasmids were transformed into BL21(DE3) cells and protein expression was performed at 50 mL scale via autoinduction for approximately 24 hours, in which the first 4 hours cultures were grown at 37°C and the remaining time at 18°C. Cultures were harvested at 4000g for 10 minutes in a tabletop centrifuge, supernatant discarded, and resuspended in approximately 30 mL lysis buffer (50 mM Tris-HCl, 150 mM NaCl, 1 mM EDTA, 0.1 mg/mL lysozyme, 0.01 mg/mL DNAse, $\frac{1}{2}$ tablet of pierce protease inhibitor tablet/50 mL culture, pH 8.0). Sonication was performed with a 4-prong head for 5 minutes total, 10s pulse on-off at 80% amplitude. The resulting lysate was clarified by centrifugation at 14000g for 30 minutes. Resulting supernatant was applied to 1 mL of Streptactin resin equilibrated with wash buffer (50 mM Tris-HCl, 150 mM NaCl, 1 mM EDTA, pH 8.0) and incubated for approximately 15 minutes with mild

agitation. Resin was subsequently washed with at least 10 CVs of wash buffer and 0.4 CVs of elution buffer (50 mM Tris-HCl, 150 mM NaCl, 1 mM EDTA, 2.5 mM desthiobiotin, pH 8.0). Another 1.3 CV of elution buffer was applied to the resin and eluate was collected for purification by size-exclusion chromatography. Samples were applied to an S200 column equilibrated once with 20 mM HEPES, 50 mM NaCl, 1 mM EDTA, pH 7.4 to ensure removal of any trace metals, then again with the same buffer without EDTA.

6.3 Negative-stain EM sample preparation

De novo designed oligomeric proteins were diluted to $\sim$ 0.1mg/mL for negative stain. 3μL of the diluted complexes were immediately negatively stained after diluting using Gilder Grids overlaid with a thin layer of carbon and 0.75% uranyl formate.

6.4 Negative-stain EM data collection, processing, and validation

Data were collected on an Talos L120C 120kV electron microscope equipped with a CETA camera. A total of $\sim$150-250 images were collected per sample by using a random defocus range of 1.3–2.3 μm, with a total exposure of between 30 and 50 e-/A2, with a pixel size of either 1.54 or 2.49 Å/pixel. All data were automatically acquired using EPU (ThermoFisher Scientific). All data processing was performed using CryoSPARC V4.0.3 [90]. The parameters of the contrast transfer function (CTF) were estimated using Patch CTF, with minimal and maximal fitting resolutions set to 40Å and 8Å, respectively. Particles were picked initially in a reference-free manner using blob picker, followed by template picking using well-defined 2D classes of intact oligomers. Particles were extracted after correcting for the effect of the CTF for each micrograph with a box size of 80 pixels, except for icosahedron HE0902, which was extracted with a box size of 180 pixels to account for its large relative size. Extracted particles were sorted by reference-free 2D classification over 20 iterations. Given the small size of these particles, 2D classification was performed both in the

presence and absence of CTF correction, with the best resulting classes selected for 3D *ab initio* reconstruction. More often than not, turning off CTF correction dramatically improved 2D class average quality. Notably, only constructs that displayed a combination of both "top-down" and "side" views (to ensure complete angular coverage) were selected for nsEM 3D reconstruction attempts. 3D *ab initio* jobs for each RFdiffusion construct displaying good angular coverage were performed by sorting into 3-4 classes, in a single attempt, in the presence and absence of the appropriate symmetry operator and compared. Resulting *ab initio* jobs which immediately converged on a map that exhibited clearly discernible features bearing a striking degree of similarity to both the 2D class average projections and computational design model were subsequently rigid-body docked against the AlphaFold2 prediction model for validation. All *ab initio* 3D maps with near perfect agreement to the design model were next run through homogenous refinement in the absence of applied symmetry to further validate their authenticity. Any 3D maps where any level of ambiguity was observed were immediately discarded. Furthermore, any 3D reconstruction attempts requiring multiple rounds of *ab initio* generation to yield convergence on a map in agreement with the design model were deemed as "low confidence" and were also discarded. For both cases, only the 2D classification results were reported in the supplementary material.

Design ID	Symmetry	10Å	20Å	30Å
HE0822	C3	0.9339	0.9521	0.9522
HE0626	C6	0.8181	0.825	0.813
HE0675	C8	0.8677	0.8877	0.8859
HE0490	D3	0.9321	0.9368	0.9274
HE0537	D4	0.942	0.9424	0.9302
HE0902	I	0.9032	0.9176	0.9163
NiB16	C4	0.866	0.883	0.8823

Supplementary Methods Table 14: Correlation of simulated model density with obtained 3D reconstruction using ChimeraX map fitting. Coefficients are calculated at three different resolutions: 10Å, 20Å, and 30Å.

6.5 CryoEM sample preparation and data collection

Symmetric oligomers

CryoEM grids were prepared by diluting protein samples with TBS 1 to 10 times immediately before applying 3.5 μL to glow-discharged 400 mesh, C-flat, 2 micron holes, 2 micron spacing, CF-2/2-4C (CF-224C-100) (Electron Microscopy Sciences) cryoEM grids. For D4 samples, 6 consecutive blots were applied in order to obtain the highest particle density [91]. Grids were blotted using a blot force of 0 and 5.5 second blot time at 100% humidity and 4°C and plunge-frozen in liquid ethane using a Vitrobot Mark IV (FEI Thermo Scientific). cryoEM grids were screened on a Glacios transmission electron microscope (FEI Thermo Scientific) operated at 200 kV and equipped with a K3 Summit direct detector. Automated Glacios data collection was carried out using SerialEM software at a nominal magnification of 36,000x (0.883 Å/pixel). Movies were acquired in counting mode fractionated in 50 frames of 200 ms at 8.5 e-/pixel/sec for a total dose of $\sim$ 65e-/Å^2. Details of data processing are illustrated in Extended Data Fig. 9.

Influenza H1 + *HA_20* minibinder Prior to freezing, 5 μM of Influzena H1 monomer (strain A/USA:Iowa/1943 H1N1) was allowed to co-incubate at 4°C for 10 minutes with 5 μM (i.e. a 3-fold molar excess relative to each H1 monomer) of the RFdiffusion *HA_20* minibinder in 150 mM NaCl, 25 mM Tris (pH = 8.0) buffer. To prepare cryoEM sample grids for the bound protein-protein complex, 3 μL of calculated 0.28 mg/mL Influenza H1 was applied to glow-discharged Quantifoil R 2/2 300 mesh copper grids overlaid with an additional thin layer of carbon. Vitrification was performed on a Mark IV Vitrobot at 22°C at 100% humidity, with a wait time of 7.5 seconds, a blot time of 0.5 seconds, and a blot force of 0 before being immediately plunged frozen into liquid

ethane. The sample grids were clipped following standard protocols before being loaded into a ThermoFisher Titan Krios 300 kV transmission electron microscope for imaging.

Data collection was performed automatically using Leginon [92] to control a ThermoFisher Titan Krios 300 kV equipped with a standalone K3 Summit direct electron detector with an energy filter. Influenza H1 bound to the RFdiffusion *HA_20* minibinder was collected using counting mode with random defocus ranges spanned between -0.7 and -1.8 μm using image shift, with five shots per hole for a total of 9,431 collected movies with a calculated pixel size of 0.84 Å/pix and a calculated total dose of 64.27 e-/Å2.

6.6 CryoEM data processing and model building

Symmetric oligomers

Multiple datasets were collected for each design and combined early on during processing. See Extended Data Fig. 9 and processing flowcharts for details. Briefly, images were manually curated to remove poor quality acquisitions such as bad ice or large regions of carbon. Dose-weighting and image alignment of all 50 frames was carried out using MotionCor2 [93] with 5X5 patch or with cryosparc v4 patch alignment tool with default parameters. Super-resolution data was binned 2X during alignment. Initial CTF parameters were estimated using CTFfind4 [94]. Particle picking was done with a Gaussian blob picker and in some cases followed by a template picker. Particles were extensively classified in 2D to remove ice and noisy particles, but unfortunately yielded practically no "top" or "tilted" view particles. However, multiple orthogonal side views down the 2-fold axis were observed displaying high agreement to the design model. Starting models for all designs were always obtained *ab initio*. FSC curves were generated using cryoSPARC. All EM maps will be deposited in the EMDB and can be found in the supplementary data.

Influenza H1 + *HA_20* minibinder

All data processing was carried out in CryoSPARC (v4.0.3) [90] and CryoSPARC Live. Alignment of movie frames was performed using Patch Motion with an estimated B-factor of 500Å^2, with a maximum alignment resolution set to 5. Defocus and astigmatism values were estimated using Patch CTF with default parameters. Influenza A Hemagglutinin particles bound to diffused minibinder *HA_20* were initially picked in a reference-free manner using Blob Picker and extracted with a box size of 340 pixels. This was followed by a round of 2D classification and subsequent template-picking using the best 2D class averages low-pass filtered to 20Å. Particles were next picked with Template Picker and were manually inspected before extracting with a box size of 340 pixels for a total of 1,077,686 particles. A round of reference-free 2D classification was next performed in CryoSPARC with a maximum alignment resolution of 6Å. The best classes which revealed clearly visible secondary-structural elements were used for 3D ab initio determination using the C1 symmetry operator. This was followed by a round of 3D heterogeneous refinement using C1 symmetry and sorting into 3 distinct classes, all of which revealed stoichiometric binding of the diffused *HA_20* minibinder to the Influenza A Hemagglutinin stem. Non-uniform 3D refinement with C3 symmetry was performed on 308,846 of the best particles, yielding a final high-resolution map with an estimated global resolution of 2.93Å, following per-particle defocus refinement. This map was sharpened using local B-factor sharpening with DeepEMhancer using the highRes deep learning model for display and model building purposes. Local resolution estimates were determined in CryoSPARC using an FSC threshold of 0.143. 3D maps for the half maps, final unsharpened maps, and the final sharpened maps were deposited in the EMDB under accession number EMD-40557.

The 2009 H1N1 pandemic influenza virus H1 glycoprotein (PDB: 3LZG) was used as an initial reference for building the cryoEM structure of the A/USA:Iowa/1943 H1N1 bound to the diffused *HA_20* minibinder. The model was manually edited and trimmed using Coot to match the A/USA:Iowa/1943 H1N1 sequence used for structural determination [95]. The *de novo* predicted

design model for the *HA_20* minibinder was used as an initial reference for building into the corresponding density. We further refined each structure in Rosetta using density-guided protocols [96]. EM density-guided molecular dynamics simulations were next performed using Interactive Structure Optimization by Local Direct Exploration (ISOLDE), with manual local inspection and guided correction of rotamers and clashes throughout simulated iterations. ISOLDE runs were performed at a simulated 25 Kelvin, with a round of Rosetta density-guided relaxation performed afterward. This process was repeated iteratively until convergence and high agreement with the map was achieved. Multiple rounds of relaxation and minimization were performed on the complete capsids, followed by human inspection for errors after each step. Throughout this process, we applied strict non-crystallographic symmetry constraints in Rosetta[96]. Phenix real-space refinement was subsequently performed as a final step before the final model quality was analyzed using Molprobity[97]. Figures were generated using either UCSF Chimera [98] or UCSF ChimeraX [99]. The final structure was deposited under PDB accession number 8SK7.

6.7 Circular dichroism experiments

For circular dichroism (CD) experiments, designs (TIM barrels or unconditional designs) were diluted to 0.2mg/ml in 20mM Tris (pH 8.0) and 50mM NaCl. Spectra were acquired on a JASCO J-1500 CD Spectrophotometer. Thermal melt analyses were performed between 25°C and 95°C, measuring CD at 222 nm. All reported measurements were acquired within the linear range of the instrument.

6.8 Bio-layer inferometry (BLI) binding experiments

BLI experiments were performed on an Octet Red96 (ForteBio) instrument, with streptavidin coated tips (Sartorius Item no. 18-5019). Buffer comprised 1X HBS-EP+ buffer (Cytiva BR100669) supplemented with 0.1% w/v bovine serum albumin. Prior to target loading, each design was tested

for binding against unloaded tips via a 120 s baseline, 120 s association and 120 s dissociation cycle. For IL7-Ra, PD-L1, Mdm2, hemagglutinin, Insulin Receptor (Sino Biological 11081-H08H-B), and TrkA, 40nM of biotinylated target protein was loaded on the tips for 300 s followed by a 60 s baseline measurement. After loading, all designs underwent a 120 s baseline, 120 s association and 120 s dissociation. For each design, a previously validated *de novo* binder [12] was included alongside 95 new designs. Four out of five positive controls were the same sequence as the previously reported designs with the addition of an MSG on the N-terminus with SNAC and His tags on the C-terminus, as per the cloning protocol described. The influenza positive control was a reengineered version of the previously reported HA binder (PDB: 7RDH) where non-interface residues were redesigned using ProteinMPNN to improve expression. Baseline measurements of unloaded tips were subtracted from their matched measurement of the loaded tip. The response was taken as the average reading from 105 - 115 s during association. Binders were classified as those whose response was $>$ 50% of the control. Up to 20 of the hits were taken forward for further titration experiments where concentration, association and dissociation times were chosen based on apparent affinity from the single point screen. Global kinetic fitting was used to determine K_Ds across the dilution series [100]. Insulin Receptor contains two independent binding sites so it was fit with a 2:1 heterogeneous ligand model and both K_Ds are reported.

6.9 Comparison of experimental success rates between design campaigns

All 5 targets chosen for *de novo* binder design were previously targeted for binder design with earlier Rosetta-based methods. The success rates in Fig 6B under "Rosetta" come from the following publications: IL-7Rα, Insulin Receptor, and TrkA are from Cao et al. [12] in Extended Data 1; PD-L1 is from an unpublished binder campaign using the method of Cao et al. [12]. For PD-L1 a binder is called a success if the upper bound K_D estimate calculated from yeast surface display

enrichment is less than 50 μM; Influenza HA is from Fleishman et al. [101], where a success rate of 2 / 88 is reported.

6.10 Isothermal titration calorimetry

Protein and NiSO4 samples were prepared in buffer containing 20 mM HEPES, 50 mM NaCl, pH 7.4. Protein sample concentrations ranged from 30 μM to 100 μM and NiSO4 samples were prepared at 10 times the effective concentration of Ni2+ coordination sites (e.g. 100 μM of a designed tetramer yields 25 μM Ni2+ coordination sites in the sample cell, and thus a NiSO4 concentration of 250 μM was used in the syringe). Isothermal titration calorimetry experiments were performed on an automated Microcal PEAQ-ITC. Fits of the resulting data for the determination of dissociation constants (K_D) was performed in the Microcal PEAQ-ITC Analysis Software.

References

[1] J. Dauparas, I. Anishchenko, N. Bennett, H. Bai, R. J. Ragotte, L. F. Milles, B. I. M. Wicky, A. Courbet, R. J. de Haas, N. Bethel, P. J. Y. Leung, T. F. Huddy, S. Pellock, D. Tischer, F. Chan, B. Koepnick, H. Nguyen, A. Kang, B. Sankaran, A. K. Bera, N. P. King, and D. Baker. Robust deep learning-based protein sequence design using ProteinMPNN. *Science*, page eadd2187, 2022.

[2] Noelia Ferruz, Steffen Schmidt, and Birte Höcker. ProtGPT2 is a deep unsupervised language model for protein design. *Nature Communications*, 13(1):4348, July 2022. ISSN 2041-1723.

[3] Jedediah M. Singer, Scott Novotney, Devin Strickland, Hugh K. Haddox, Nicholas Leiby, Gabriel J. Rocklin, Cameron M. Chow, Anindya Roy, Asim K. Bera, Francis C. Motta, Longxing Cao, Eva-Maria Strauch, Tamuka M. Chidyausiku, Alex Ford, Ethan Ho, Alexander Zaitzeff, Craig O. Mackenzie, Hamed Eramian, Frank DiMaio, Gevorg Grigoryan, Matthew Vaughn, Lance J. Stewart, David Baker, and Eric Klavins. Large-scale design and refinement of stable proteins using sequence-only models. *PLOS ONE*, 17(3):e0265020, March 2022. ISSN 1932-6203.

[4] Jue Wang, Sidney Lisanza, David Juergens, Doug Tischer, Joseph L. Watson, Karla M. Castro, Robert Ragotte, Amijai Saragovi, Lukas F. Milles, Minkyung Baek, Ivan Anishchenko, Wei Yang, Derrick R. Hicks, Marc Expòsit, Thomas Schlichthaerle, Jung-Ho Chun, Justas Dauparas, Nathaniel Bennett, Basile I. M. Wicky, Andrew Muenks, Frank DiMaio, Bruno Correia, Sergey Ovchinnikov, and David Baker. Scaffolding protein functional sites using deep learning. *Science*, 377(6604):387–394, 2022.

[5] Brian L Trippe, Jason Yim, Doug Tischer, Tamara Broderick, David Baker, Regina Barzilay,

and Tommi Jaakkola. Diffusion probabilistic modeling of protein backbones in 3D for the motif-scaffolding problem. In *International Conference on Learning Representations*, 2023.

[6] Ivan Anishchenko, Samuel J. Pellock, Tamuka M. Chidyausiku, Theresa A. Ramelot, Sergey Ovchinnikov, Jingzhou Hao, Khushboo Bafna, Christoffer Norn, Alex Kang, Asim K. Bera, Frank DiMaio, Lauren Carter, Cameron M. Chow, Gaetano T. Montelione, and David Baker. De novo protein design by deep network hallucination. *Nature*, 2021.

[7] B. I. M. Wicky, L. F. Milles, A. Courbet, R. J. Ragotte, J. Dauparas, E. Kinfu, S. Tipps, R. D. Kibler, M. Baek, F. DiMaio, X. Li, L. Carter, A. Kang, H. Nguyen, A. K. Bera, and D. Baker. Hallucinating symmetric protein assemblies. *Science*, 378(6615):56–61, 2022.

[8] Namrata Anand and Tudor Achim. Protein Structure and Sequence Generation with Equivariant Denoising Diffusion Probabilistic Models, 2022.

[9] Shitong Luo, Yufeng Su, Xingang Peng, Sheng Wang, Jian Peng, and Jianzhu Ma. Antigen-Specific Antibody Design and Optimization with Diffusion-Based Generative Models. page 13.

[10] Jascha Sohl-Dickstein, Eric Weiss, Niru Maheswaranathan, and Surya Ganguli. Deep unsupervised learning using nonequilibrium thermodynamics. In *International Conference on Machine Learning*, pages 2256–2265. PMLR, 2015.

[11] Jonathan Ho, Ajay Jain, and Pieter Abbeel. Denoising diffusion probabilistic models. *Advances in Neural Information Processing Systems*, 33:6840–6851, 2020.

[12] Longxing Cao, Brian Coventry, Inna Goreshnik, Buwei Huang, William Sheffler, Joon Sung Park, Kevin M. Jude, Iva Marković, Rameshwar U. Kadam, Koen H. G. Verschueren, Kenneth Verstraete, Scott Thomas Russell Walsh, Nathaniel Bennett, Ashish Phal, Aerin Yang, Lisa Kozodoy, Michelle DeWitt, Lora Picton, Lauren Miller, Eva-Maria Strauch, Nicholas D.

DeBouver, Allison Pires, Asim K. Bera, Samer Halabiya, Bradley Hammerson, Wei Yang, Steffen Bernard, Lance Stewart, Ian A. Wilson, Hannele Ruohola-Baker, Joseph Schlessinger, Sangwon Lee, Savvas N. Savvides, K. Christopher Garcia, and David Baker. Design of protein-binding proteins from the target structure alone. *Nature*, 605(7910):551–560, May 2022. ISSN 1476-4687.

[13] Brian Kuhlman, Gautam Dantas, Gregory C. Ireton, Gabriele Varani, Barry L. Stoddard, and David Baker. Design of a novel globular protein fold with atomic-level accuracy. *Science*, 302 (5649):1364–1368, 2003. doi: 10.1126/science.1089427. URL `https://www.science.org/doi/abs/10.1126/science.1089427`.

[14] Aditya Ramesh, Mikhail Pavlov, Gabriel Goh, Scott Gray, Chelsea Voss, Alec Radford, Mark Chen, and Ilya Sutskever. Zero-Shot Text-to-Image Generation, February 2021.

[15] Chitwan Saharia, William Chan, Saurabh Saxena, Lala Li, Jay Whang, Emily Denton, Seyed Kamyar Seyed Ghasemipour, Burcu Karagol Ayan, S. Sara Mahdavi, Rapha Gontijo Lopes, Tim Salimans, Jonathan Ho, David J. Fleet, and Mohammad Norouzi. Photorealistic Text-to-Image Diffusion Models with Deep Language Understanding, May 2022.

[16] Kevin E. Wu, Kevin K. Yang, Rianne van den Berg, James Y. Zou, Alex X. Lu, and Ava P. Amini. Protein structure generation via folding diffusion. September 2022.

[17] John Jumper, Richard Evans, Alexander Pritzel, Tim Green, Michael Figurnov, Olaf Ronneberger, Kathryn Tunyasuvunakool, Russ Bates, Augustin Žídek, Anna Potapenko, Alex Bridgland, Clemens Meyer, Simon A. A. Kohl, Andrew J. Ballard, Andrew Cowie, Bernardino Romera-Paredes, Stanislav Nikolov, Rishub Jain, Jonas Adler, Trevor Back, Stig Petersen, David Reiman, Ellen Clancy, Michal Zielinski, Martin Steinegger, Michalina Pacholska, Tamas Berghammer, Sebastian Bodenstein, David Silver, Oriol Vinyals, Andrew W. Se-

nior, Koray Kavukcuoglu, Pushmeet Kohli, and Demis Hassabis. Highly accurate protein structure prediction with AlphaFold. *Nature*, 596(7873):583–589, 2021.

[18] Minkyung Baek, Frank DiMaio, Ivan Anishchenko, Justas Dauparas, Sergey Ovchinnikov, Gyu Rie Lee, Jue Wang, Qian Cong, Lisa N. Kinch, R. Dustin Schaeffer, Claudia Millán, Hahnbeom Park, Carson Adams, Caleb R. Glassman, Andy DeGiovanni, Jose H. Pereira, Andria V. Rodrigues, Alberdina A. van Dijk, Ana C. Ebrecht, Diederik J. Opperman, Theo Sagmeister, Christoph Buhlheller, Tea Pavkov-Keller, Manoj K. Rathinaswamy, Udit Dalwadi, Calvin K. Yip, John E. Burke, K. Christopher Garcia, Nick V. Grishin, Paul D. Adams, Randy J. Read, and David Baker. Accurate prediction of protein structures and interactions using a three-track neural network. *Science*, 373(6557):871–876, 2021.

[19] Joseph L. Watson, Asim Bera, David Juergens, Jue Wang, and David Baker. X-ray crystallographic validation of design from this paper | Science | AAAS, July 2022.

[20] Ruidong Wu, Fan Ding, Rui Wang, Rui Shen, Xiwen Zhang, Shitong Luo, Chenpeng Su, Zuofan Wu, Qi Xie, Bonnie Berger, Jianzhu Ma, and Jian Peng. High-resolution de novo structure prediction from primary sequence, July 2022.

[21] Zeming Lin, Halil Akin, Roshan Rao, Brian Hie, Zhongkai Zhu, Wenting Lu, Allan dos Santos Costa, Maryam Fazel-Zarandi, Tom Sercu, Sal Candido, et al. Language models of protein sequences at the scale of evolution enable accurate structure prediction. *bioRxiv*, 2022.

[22] H. M. Berman, J. Westbrook, Z. Feng, G. Gilliland, T. N. Bhat, H. Weissig, I. N. Shindyalov, and P. E. Bourne. The Protein Data Bank. *Nucleic acids research*, 28(1):235–242, January 2000. ISSN 0305-1048 1362-4962. doi: 10.1093/nar/28.1.235. Place: England.

[23] Valentin De Bortoli, Emile Mathieu, Michael John Hutchinson, James Thornton, Yee Whye

Teh, and Arnaud Doucet. Riemannian score-based generative modelling. In *Advances in Neural Information Processing Systems*, 2022.

[24] Adam Leach, Sebastian M Schmon, Matteo T Degiacomi, and Chris G Willcocks. Denoising diffusion probabilistic models on SO(3) for rotational alignment. In *ICLR 2022 Workshop on Geometrical and Topological Representation Learning*, 2022.

[25] Ting Chen, Ruixiang Zhang, and Geoffrey Hinton. Analog bits: Generating discrete data using diffusion models with self-conditioning. In *International Conference on Machine Learning Research*, 2023.

[26] Nathaniel R Bennett, Brian Coventry, Inna Goreshnik, Buwei Huang, Aza Allen, Dionne Vafeados, Ying Po Peng, Justas Dauparas, Minkyung Baek, Lance Stewart, et al. Improving de novo protein binder design with deep learning. *Nature Communications*, 14(1):2625, 2023.

[27] Namrata Anand and Possu Huang. Generative modeling for protein structures. In *Advances in Neural Information Processing Systems*, 2018.

[28] John Ingraham, Max Baranov, Zak Costello, Vincent Frappier, Ahmed Ismail, Shan Tie, Wujie Wang, Vincent Xue, Fritz Obermeyer, Andrew Beam, and Gevorg Grigoryan. Illuminating protein space with a programmable generative model. *bioRxiv*, 2022. doi: 10.1101/2022.12.01.518682. URL `https://www.biorxiv.org/content/early/2022/12/02/2022.12.01.518682`.

[29] Jin Sub Lee and Philip M. Kim. ProteinSGM: Score-based generative modeling for de novo protein design. *bioRxiv*, 2022.

[30] José Nelson Onuchic, Zaida Luthey-Schulten, and Peter G. Wolynes. Theory of protein folding: The energy landscape perspective. *Annual Review of Physical Chemistry*, 48(1):

545–600, 1997. doi: 10.1146/annurev.physchem.48.1.545. URL https://doi.org/10.1146/annurev.physchem.48.1.545. PMID: 9348663.

[31] Michael Jendrusch, Jan O. Korbel, and S. Kashif Sadiq. AlphaDesign: A de novo protein design framework based on AlphaFold, October 2021.

[32] Benjamin Basanta, Matthew J. Bick, Asim K. Bera, Christoffer Norn, Cameron M. Chow, Lauren P. Carter, Inna Goreshnik, Frank Dimaio, and David Baker. An enumerative algorithm for de novo design of proteins with diverse pocket structures. *Proceedings of the National Academy of Sciences*, 117(36):22135–22145, September 2020. ISSN 0027-8424, 1091-6490.

[33] Xingjie Pan, Michael C. Thompson, Yang Zhang, Lin Liu, James S. Fraser, Mark J. S. Kelly, and Tanja Kortemme. Expanding the space of protein geometries by computational design of de novo fold families. *Science*, 369(6507):1132–1136, August 2020.

[34] Jessica Marcandalli, Brooke Fiala, Sebastian Ols, Michela Perotti, Willem de van der Schueren, Joost Snijder, Edgar Hodge, Mark Benhaim, Rashmi Ravichandran, Lauren Carter, Will Sheffler, Livia Brunner, Maria Lawrenz, Patrice Dubois, Antonio Lanzavecchia, Federica Sallusto, Kelly K. Lee, David Veesler, Colin E. Correnti, Lance J. Stewart, David Baker, Karin Loré, Laurent Perez, and Neil P. King. Induction of Potent Neutralizing Antibody Responses by a Designed Protein Nanoparticle Vaccine for Respiratory Syncytial Virus. *Cell*, 176(6):1420–1431.e17, March 2019. ISSN 0092-8674, 1097-4172.

[35] Gabriel L. Butterfield, Marc J. Lajoie, Heather H. Gustafson, Drew L. Sellers, Una Nattermann, Daniel Ellis, Jacob B. Bale, Sharon Ke, Garreck H. Lenz, Angelica Yehdego, Rashmi Ravichandran, Suzie H. Pun, Neil P. King, and David Baker. Evolution of a designed protein assembly encapsulating its own RNA genome. *Nature*, 552(7685):415–420, December 2017. ISSN 1476-4687.

[36] D. S. Goodsell and A. J. Olson. Structural symmetry and protein function. *Annual Review of Biophysics and Biomolecular Structure*, 29:105–153, 2000.

[37] Reinhard Sterner and Birte Höcker. Catalytic versatility, stability, and evolution of the (betaalpha)8-barrel enzyme fold. *Chemical reviews*, 105(11):4038–4055, November 2005. ISSN 0009-2665. doi: 10.1021/cr030191z. Place: United States.

[38] Fabian Sesterhenn, Che Yang, Jaume Bonet, Johannes T. Cramer, Xiaolin Wen, Yimeng Wang, Chi-I. Chiang, Luciano A. Abriata, Iga Kucharska, Giacomo Castoro, Sabrina S. Vollers, Marie Galloux, Elie Dheilly, Stéphane Rosset, Patricia Corthésy, Sandrine Georgeon, Mélanie Villard, Charles-Adrien Richard, Delphyne Descamps, Teresa Delgado, Elisa Oricchio, Marie-Anne Rameix-Welti, Vicente Más, Sean Ervin, Jean-François Eléouët, Sabine Riffault, John T. Bates, Jean-Philippe Julien, Yuxing Li, Theodore Jardetzky, Thomas Krey, and Bruno E. Correia. De novo protein design enables the precise induction of RSV-neutralizing antibodies. *Science*, 368(6492), May 2020. ISSN 1095-9203 0036-8075.

[39] Che Yang, Fabian Sesterhenn, Jaume Bonet, Eva A. van Aalen, Leo Scheller, Luciano A. Abriata, Johannes T. Cramer, Xiaolin Wen, Stéphane Rosset, Sandrine Georgeon, Theodore Jardetzky, Thomas Krey, Martin Fussenegger, Maarten Merkx, and Bruno E. Correia. Bottom-up de novo design of functional proteins with complex structural features. *Nature chemical biology*, 17(4):492–500, April 2021. ISSN 1552-4469 1552-4450.

[40] Anum Glasgow, Jeff Glasgow, Daniel Limonta, Paige Solomon, Irene Lui, Yang Zhang, Matthew A. Nix, Nicholas J. Rettko, Shoshana Zha, Rachel Yamin, Kevin Kao, Oren S. Rosenberg, Jeffrey V. Ravetch, Arun P. Wiita, Kevin K. Leung, Shion A. Lim, Xin X. Zhou, Tom C. Hobman, Tanja Kortemme, and James A. Wells. Engineered ACE2 receptor traps potently neutralize SARS-CoV-2. *Proceedings of the National Academy of Sciences of the United States of America*, 117(45):28046–28055, November 2020. ISSN 0027-8424.

[41] Patrick Chène. Inhibiting the p53-MDM2 interaction: an important target for cancer therapy. *Nature reviews. Cancer*, 3(2):102–109, February 2003. ISSN 1474-175X. doi: 10.1038/nrc991. Place: England.

[42] P. H. Kussie, S. Gorina, V. Marechal, B. Elenbaas, J. Moreau, A. J. Levine, and N. P. Pavletich. Structure of the MDM2 oncoprotein bound to the p53 tumor suppressor transactivation domain. *Science (New York, N.Y.)*, 274(5289):948–953, November 1996. ISSN 0036-8075. doi: 10.1126/science.274.5289.948. Place: United States.

[43] Andrew C. Hunt, James Brett Case, Young-Jun Park, Longxing Cao, Kejia Wu, Alexandra C. Walls, Zhuoming Liu, John E. Bowen, Hsien-Wei Yeh, Shally Saini, Louisa Helms, Yan Ting Zhao, Tien-Ying Hsiang, Tyler N. Starr, Inna Goreshnik, Lisa Kozodoy, Lauren Carter, Rashmi Ravichandran, Lydia B. Green, Wadim L. Matochko, Christy A. Thomson, Bastian Vögeli, Antje Krüger, Laura A. VanBlargan, Rita E. Chen, Baoling Ying, Adam L. Bailey, Natasha M. Kafai, Scott E. Boyken, Ajasja Ljubetič, Natasha Edman, George Ueda, Cameron M. Chow, Max Johnson, Amin Addetia, Mary Jane Navarro, Nuttada Panpradist, Michael Gale, Benjamin S. Freedman, Jesse D. Bloom, Hannele Ruohola-Baker, Sean P. J. Whelan, Lance Stewart, Michael S. Diamond, David Veesler, Michael C. Jewett, and David Baker. Multivalent designed proteins neutralize SARS-CoV-2 variants of concern and confer protection against infection in mice. *Science translational medicine*, 14(646):eabn1252, May 2022. ISSN 1946-6234.

[44] Joshua Silverman, Qiang Liu, Alice Bakker, Wayne To, Amy Duguay, Ben M. Alba, Richard Smith, Alberto Rivas, Peng Li, Hon Le, Erik Whitehorn, Kevin W. Moore, Candace Swimmer, Victor Perlroth, Martin Vogt, Joost Kolkman, and Willem Pim C. Stemmer. Multivalent avimer proteins evolved by exon shuffling of a family of human receptor domains. *Nature Biotechnology*, 23(12):1556–1561, December 2005. ISSN 1087-0156.

[45] Laurent Detalle, Thomas Stohr, Concepción Palomo, Pedro A. Piedra, Brian E. Gilbert, Vicente Mas, Andrena Millar, Ultan F. Power, Catelijne Stortelers, Koen Allosery, José A. Melero, and Erik Depla. Generation and Characterization of ALX-0171, a Potent Novel Therapeutic Nanobody for the Treatment of Respiratory Syncytial Virus Infection. *Antimicrobial Agents and Chemotherapy*, 60(1):6–13, January 2016. ISSN 1098-6596.

[46] Eva-Maria Strauch, Steffen M. Bernard, David La, Alan J. Bohn, Peter S. Lee, Caitlin E. Anderson, Travis Nieusma, Carly A. Holstein, Natalie K. Garcia, Kathryn A. Hooper, Rashmi Ravichandran, Jorgen W. Nelson, William Sheffler, Jesse D. Bloom, Kelly K. Lee, Andrew B. Ward, Paul Yager, Deborah H. Fuller, Ian A. Wilson, and David Baker. Computational design of trimeric influenza-neutralizing proteins targeting the hemagglutinin receptor binding site. *Nature Biotechnology*, 35(7):667–671, July 2017. ISSN 1546-1696.

[47] Seyhan Boyoglu-Barnum, Daniel Ellis, Rebecca A. Gillespie, Geoffrey B. Hutchinson, Young-Jun Park, Syed M. Moin, Oliver J. Acton, Rashmi Ravichandran, Mike Murphy, Deleah Pettie, Nick Matheson, Lauren Carter, Adrian Creanga, Michael J. Watson, Sally Kephart, Sila Ataca, John R. Vaile, George Ueda, Michelle C. Crank, Lance Stewart, Kelly K. Lee, Miklos Guttman, David Baker, John R. Mascola, David Veesler, Barney S. Graham, Neil P. King, and Masaru Kanekiyo. Quadrivalent influenza nanoparticle vaccines induce broad protection. *Nature*, 592(7855):623–628, April 2021. ISSN 1476-4687.

[48] Alexandra C. Walls, Brooke Fiala, Alexandra Schäfer, Samuel Wrenn, Minh N. Pham, Michael Murphy, Longping V. Tse, Laila Shehata, Megan A. O'Connor, Chengbo Chen, Mary Jane Navarro, Marcos C. Miranda, Deleah Pettie, Rashmi Ravichandran, John C. Kraft, Cassandra Ogohara, Anne Palser, Sara Chalk, E.-Chiang Lee, Kathryn Guerriero, Elizabeth Kepl, Cameron M. Chow, Claire Sydeman, Edgar A. Hodge, Brieann Brown, Jim T. Fuller, Kenneth H. Dinnon, Lisa E. Gralinski, Sarah R. Leist, Kendra L. Gully, Thomas B. Lewis, Miklos Guttman, Helen Y. Chu, Kelly K. Lee, Deborah H. Fuller, Ralph S. Baric,

Paul Kellam, Lauren Carter, Marion Pepper, Timothy P. Sheahan, David Veesler, and Neil P. King. Elicitation of Potent Neutralizing Antibody Responses by Designed Protein Nanoparticle Vaccines for SARS-CoV-2. *Cell*, 183(5):1367–1382.e17, November 2020. ISSN 0092-8674, 1097-4172.

[49] Eric N. Salgado, Richard A. Lewis, Susanne Mossin, Arnold L. Rheingold, and F. Akif Tezcan. Control of protein oligomerization symmetry by metal coordination: C2 and C3 symmetrical assemblies through Cu(II) and Ni(II) coordination. *Inorganic chemistry*, 48(7):2726–2728, April 2009. ISSN 1520-510X 0020-1669. doi: 10.1021/ic9001237. Place: United States.

[50] Eric N. Salgado, Xavier I. Ambroggio, Jeffrey D. Brodin, Richard A. Lewis, Brian Kuhlman, and F. Akif Tezcan. Metal templated design of protein interfaces. *Proceedings of the National Academy of Sciences of the United States of America*, 107(5):1827–1832, February 2010. ISSN 1091-6490 0027-8424. doi: 10.1073/pnas.0906852107. Place: United States.

[51] Alfredo Quijano-Rubio, Umut Y. Ulge, Carl D. Walkey, and Daniel-Adriano Silva. The advent of de novo proteins for cancer immunotherapy. *Current Opinion in Chemical Biology*, 56:119–128, June 2020. ISSN 1879-0402.

[52] Aaron Chevalier, Daniel-Adriano Silva, Gabriel J. Rocklin, Derrick R. Hicks, Renan Vergara, Patience Murapa, Steffen M. Bernard, Lu Zhang, Kwok-Ho Lam, Guorui Yao, Christopher D. Bahl, Shin-Ichiro Miyashita, Inna Goreshnik, James T. Fuller, Merika T. Koday, Cody M. Jenkins, Tom Colvin, Lauren Carter, Alan Bohn, Cassie M. Bryan, D. Alejandro Fernández-Velasco, Lance Stewart, Min Dong, Xuhui Huang, Rongsheng Jin, Ian A. Wilson, Deborah H. Fuller, and David Baker. Massively parallel de novo protein design for targeted therapeutics. *Nature*, 550(7674):74–79, October 2017. ISSN 1476-4687 0028-0836. doi: 10.1038/nature23912. Place: England.

[53] Christopher Frank, Ali Khoshouei, Yosta de Stigter, Dominik Schiewitz, Shihao Feng,

Sergey Ovchinnikov, and Hendrik Dietz. Efficient and scalable de novo protein design using a relaxed sequence space. *bioRxiv*, 2023. doi: 10.1101/2023.02.24.529906. URL `https://www.biorxiv.org/content/early/2023/02/25/2023.02.24.529906`.

[54] Susana Vázquez Torres, Philip J. Y. Leung, Isaac D. Lutz, Preetham Venkatesh, Joseph L. Watson, Fabian Hink, Huu-Hien Huynh, Andy Hsien-Wei Yeh, David Juergens, Nathaniel R. Bennett, Andrew N. Hoofnagle, Eric Huang, Michael J MacCoss, Marc Expòsit, Gyu Rie Lee, Elif Nihal Korkmaz, Jeff Nivala, Lance Stewart, Joseph M. Rogers, and David Baker. De novo design of high-affinity protein binders to bioactive helical peptides. *bioRxiv*, 2022.

[55] Minkyung Baek, Ryan McHugh, Ivan Anishchenko, David Baker, and Frank DiMaio. Accurate prediction of nucleic acid and protein-nucleic acid complexes using RoseTTAFoldNA. *bioRxiv*, 2022.

[56] Andy Hsien-Wei Yeh, Christoffer Norn, Yakov Kipnis, Doug Tischer, Samuel J. Pellock, Declan Evans, Pengchen Ma, Gyu Rie Lee, Jason Z. Zhang, Ivan Anishchenko, Brian Coventry, Longxing Cao, Justas Dauparas, Samer Halabiya, Michelle DeWitt, Lauren Carter, K. N. Houk, and David Baker. De novo design of luciferases using deep learning. *Nature*, 614(7949): 774–780, 2023.

[57] António J M Ribeiro, Gemma L Holliday, Nicholas Furnham, Jonathan D Tyzack, Katherine Ferris, and Janet M Thornton. Mechanism and Catalytic Site Atlas (M-CSA): a database of enzyme reaction mechanisms and active sites. *Nucleic Acids Research*, 46(Database issue): D618–D623, January 2018. ISSN 0305-1048.

[58] Andrew Leaver-Fay, Michael Tyka, Steven M. Lewis, Oliver F. Lange, James Thompson, Ron Jacak, Kristian Kaufman, P. Douglas Renfrew, Colin A. Smith, Will Sheffler, Ian W. Davis, Seth Cooper, Adrien Treuille, Daniel J. Mandell, Florian Richter, Yih-En Andrew Ban, Sarel J. Fleishman, Jacob E. Corn, David E. Kim, Sergey Lyskov, Monica Berrondo, Stuart

Mentzer, Zoran Popović, James J. Havranek, John Karanicolas, Rhiju Das, Jens Meiler, Tanja Kortemme, Jeffrey J. Gray, Brian Kuhlman, David Baker, and Philip Bradley. ROSETTA3: an object-oriented software suite for the simulation and design of macromolecules. *Methods in Enzymology*, 487:545–574, 2011.

[59] Maxim V. Shapovalov and Roland L. Dunbrack. A Smoothed Backbone-Dependent Rotamer Library for Proteins Derived from Adaptive Kernel Density Estimates and Regressions. *Structure*, 19(6):844–858, June 2011. ISSN 0969-2126.

[60] Minkyung Baek, Ivan Anishchenko, Ian R. Humphreys, Qian Cong, David Baker, and Frank DiMaio. Efficient and accurate prediction of protein structure using rosettafold2. *bioRxiv*, 2023. doi: 10.1101/2023.05.24.542179. URL https://www.biorxiv.org/content/early/2023/05/25/2023.05.24.542179.

[61] Nathaniel Thomas, Tess Smidt, Steven Kearnes, Lusann Yang, Li Li, Kai Kohlhoff, and Patrick Riley. Tensor field networks: Rotation-and translation-equivariant neural networks for 3D point clouds. *arXiv preprint arXiv:1802.08219*, 2018.

[62] Fabian Fuchs, Daniel Worrall, Volker Fischer, and Max Welling. Se (3)-transformers: 3d roto-translation equivariant attention networks. *Advances in Neural Information Processing Systems*, 33:1970–1981, 2020.

[63] Jianyi Yang, Ivan Anishchenko, Hahnbeom Park, Zhenling Peng, Sergey Ovchinnikov, and David Baker. Improved protein structure prediction using predicted interresidue orientations. *Proceedings of the National Academy of Sciences*, 117(3):1496–1503, 2020.

[64] Jason Yim, Brian L Trippe, Valentin De Bortoli, Emile Mathieu, Arnaud Doucet, Regina Barzilay, and Tommi Jaakkola. SE(3) diffusion model with application to protein backbone generation. *arXiv preprint arXiv:2302.02277*, 2023.

[65] Yang Song, Jascha Sohl-Dickstein, Diederik P Kingma, Abhishek Kumar, Stefano Ermon, and Ben Poole. Score-based generative modeling through stochastic differential equations. In *International Conference on Learning Representations*, 2021.

[66] Joan Sola, Jeremie Deray, and Dinesh Atchuthan. A micro lie theory for state estimation in robotics. *arXiv preprint arXiv:1812.01537*, 2018.

[67] Dmitry I Nikolayev and Tatjana I Savyolov. Normal distribution on the rotation group SO (3). *Textures and Microstructures*, 29, 1970.

[68] Pascal Vincent. A connection between score matching and denoising autoencoders. *Neural computation*, 23(7):1661–1674, 2011.

[69] Pierre M. Larochelle, Andrew P. Murray, and Jorge Angeles. A distance metric for finite sets of rigid-body displacements via the polar decomposition. *Journal of Mechanical Design*, 129 (8):883–886, 2007.

[70] Calvin Luo. Understanding diffusion models: A unified perspective. *arXiv preprint arXiv:2208.11970*, 2022.

[71] Du Q. Huynh. Metrics for 3D rotations: Comparison and analysis. *Journal of Mathematical Imaging and Vision*, 35(2):155–164, 2009.

[72] Simon Batzner, Albert Musaelian, Lixin Sun, Mario Geiger, Jonathan P Mailoa, Mordechai Kornbluth, Nicola Molinari, Tess E Smidt, and Boris Kozinsky. E (3)-equivariant graph neural networks for data-efficient and accurate interatomic potentials. *Nature communications*, 13(1):2453, 2022.

[73] Minkai Xu, Lantao Yu, Yang Song, Chence Shi, Stefano Ermon, and Jian Tang. Geodiff: A geometric diffusion model for molecular conformation generation. In *International Conference on Learning Representations*, 2022.

[74] Chitwan Saharia, William Chan, Huiwen Chang, Chris A. Lee, Jonathan Ho, Tim Salimans, David J. Fleet, and Mohammad Norouzi. Palette: Image-to-Image Diffusion Models, May 2022.

[75] Wolfgang Kabsch and Christian Sander. Dictionary of protein secondary structure: pattern recognition of hydrogen-bonded and geometrical features. *Biopolymers: Original Research on Biomolecules*, 22(12):2577–2637, 1983.

[76] Robert Verkuil, Ori Kabeli, Yilun Du, Basile I. M. Wicky, Lukas F. Milles, Justas Dauparas, David Baker, Sergey Ovchinnikov, Tom Sercu, and Alexander Rives. Language models generalize beyond natural proteins. *bioRxiv*, 2022.

[77] Christopher Frank, Ali Khoshouei, Yosta de Stigter, Dominik Schiewitz, Shihao Feng, Sergey Ovchinnikov, and Hendrik Dietz. Efficient and scalable de novo protein design using a relaxed sequence space. *bioRxiv*, 2023.

[78] Linna An, Derrick R Hicks, Dmitri Zorine, Justas Dauparas, Basile I. M. Wicky, Lukas F. Milles, Alexis Courbet, Asim K. Bera, Hannah Nguyen, Alex Kang, Lauren Carter, and David Baker. Hallucination of closed repeat proteins containing central pockets. *bioRxiv*, 2022.

[79] Casper Goverde, Benedict Wolf, Hamed Khakzad, Stéphane Rosset, and Bruno E. Correia. De novo protein design by inversion of the AlphaFold structure prediction network. *bioRxiv*, 2022.

[80] David E. Kim, Davin R. Jensen, David Feldman, Doug Tischer, Ayesha Saleem, Cameron M. Chow, Xinting Li, Lauren Carter, Lukas Milles, Hannah Nguyen, Alex Kang, Asim K. Bera, Francis C. Peterson, Brian F. Volkman, Sergey Ovchinnikov, and David Baker. De novo design of small beta barrel proteins. *Proceedings of the National Academy of Sciences*, 120 (11):e2207974120, March 2023.

[81] Jinrui Xu and Yang Zhang. How significant is a protein structure similarity with tm-score = 0.5? *Bioinformatics*, 26(7):889–895, 2010.

[82] Yang Zhang and Jeffrey Skolnick. Scoring function for automated assessment of protein structure template quality. *Proteins*, 57(4):702–710, December 2004. ISSN 1097-0134 0887-3585.

[83] Claudia Andreini, Gabriele Cavallaro, Serena Lorenzini, and Antonio Rosato. MetalPDB: a database of metal sites in biological macromolecular structures. *Nucleic Acids Research*, 41 (D1):D312–D319, January 2013. ISSN 0305-1048.

[84] Sarel J. Fleishman, Timothy A. Whitehead, Damian C. Ekiert, Cyrille Dreyfus, Jacob E. Corn, Eva-Maria Strauch, Ian A. Wilson, and David Baker. Computational design of proteins targeting the conserved stem region of influenza hemagglutinin. *Science*, 332(6031):816–821, 2011. doi: 10.1126/science.1202617.

[85] LLC Schrödinger and Warren DeLano. PyMOL, May 2020.

[86] J. D. Hunter. Matplotlib: A 2D graphics environment. *Computing in Science & Engineering*, 9(3):90–95, 2007.

[87] Michael Waskom, Olga Botvinnik, Drew O'Kane, Paul Hobson, Saulius Lukauskas, David C. Gemperline, Tom Augspurger, Yaroslav Halchenko, John B. Cole, Jordi Warmenhoven, Julian de Ruiter, Cameron Pye, Stephan Hoyer, Jake Vanderplas, Santi Villalba, Gero Kunter, Eric Quintero, Pete Bachant, Marcel Martin, Kyle Meyer, Alistair Miles, Yoav Ram, Tal Yarkoni, Mike Lee Williams, Constantine Evans, Clark Fitzgerald, Brian, Chris Fonnesbeck, Antony Lee, and Adel Qalieh. mwaskom/seaborn: v0.8.1 (September 2017), September 2017.

[88] Pauli Virtanen, Ralf Gommers, Travis E. Oliphant, Matt Haberland, Tyler Reddy, David Cournapeau, Evgeni Burovski, Pearu Peterson, Warren Weckesser, Jonathan Bright, Stéfan J.

van der Walt, Matthew Brett, Joshua Wilson, K. Jarrod Millman, Nikolay Mayorov, Andrew R. J. Nelson, Eric Jones, Robert Kern, Eric Larson, C. J. Carey, İlhan Polat, Yu Feng, Eric W. Moore, Jake VanderPlas, Denis Laxalde, Josef Perktold, Robert Cimrman, Ian Henriksen, E. A. Quintero, Charles R. Harris, Anne M. Archibald, Antônio H. Ribeiro, Fabian Pedregosa, and Paul van Mulbregt. SciPy 1.0: fundamental algorithms for scientific computing in Python. *Nature Methods*, 17(3):261–272, March 2020. ISSN 1548-7105.

[89] Bobo Dang, Marco Mravic, Hailin Hu, Nathan Schmidt, Bruk Mensa, and William F DeGrado. SNAC-tag for sequence-specific chemical protein cleavage. *Nature methods*, 16(4): 319–322, 2019.

[90] Ali Punjani, John L Rubinstein, David J Fleet, and Marcus A Brubaker. cryoSPARC: algorithms for rapid unsupervised cryo-EM structure determination. *Nature methods*, 14(3): 290–296, 2017.

[91] Joost Snijder, Andrew J Borst, Annie Dosey, Alexandra C Walls, Anika Burrell, Vijay S Reddy, Justin M Kollman, and David Veesler. Vitrification after multiple rounds of sample application and blotting improves particle density on cryo-electron microscopy grids. *Journal of structural biology*, 198(1):38–42, 2017.

[92] Anchi Cheng, Carl Negro, Jessica F. Bruhn, William J. Rice, Sargis Dallakyan, Edward T. Eng, David G. Waterman, Clinton S. Potter, and Bridget Carragher. Leginon: New features and applications. *Protein Science : A Publication of the Protein Society*, 30(1):136–150, January 2021. ISSN 0961-8368.

[93] Shawn Q Zheng, Eugene Palovcak, Jean-Paul Armache, Kliment A Verba, Yifan Cheng, and David A Agard. MotionCor2: anisotropic correction of beam-induced motion for improved cryo-electron microscopy. *Nature methods*, 14(4):331–332, 2017.

[94] Alexis Rohou and Nikolaus Grigorieff. Ctffind4: Fast and accurate defocus estimation from electron micrographs. *Journal of structural biology*, 192(2):216–221, 2015.

[95] Paul Emsley, Bernhard Lohkamp, William G. Scott, and Kevin Cowtan. Features and development of coot. *Acta Crystallographica Section D - Biological Crystallography*, 66:486–501, 2010.

[96] Julia Koehler Leman, Brian D Weitzner, Steven M Lewis, Jared Adolf-Bryfogle, Nawsad Alam, Rebecca F Alford, Melanie Aprahamian, David Baker, Kyle A Barlow, Patrick Barth, et al. Macromolecular modeling and design in rosetta: recent methods and frameworks. *Nature methods*, 17(7):665–680, 2020.

[97] Christopher J Williams, Jeffrey J Headd, Nigel W Moriarty, Michael G Prisant, Lizbeth L Videau, Lindsay N Deis, Vishal Verma, Daniel A Keedy, Bradley J Hintze, Vincent B Chen, et al. Molprobity: More and better reference data for improved all-atom structure validation. *Protein Science*, 27(1):293–315, 2018.

[98] Eric F Pettersen, Thomas D Goddard, Conrad C Huang, Gregory S Couch, Daniel M Greenblatt, Elaine C Meng, and Thomas E Ferrin. Ucsf chimera—a visualization system for exploratory research and analysis. *Journal of computational chemistry*, 25(13):1605–1612, 2004.

[99] Pettersen EF, Goddard TD, Huang CC, Meng EC, Couch GS, Croll TI, Morris JH, and Ferrin TE. UCSF ChimeraX: Structure visualization for researchers, educators, and developers. *Protein Science*, 30:70–82, 2021.

[100] Wolfgang Ott, Ellis Durner, and Hermann E Gaub. Enzyme-mediated, site-specific protein coupling strategies for surface-based binding assays. *Angewandte Chemie*, 130(39):12848–12851, 2018.

[101] Sarel J Fleishman, Timothy A Whitehead, Damian C Ekiert, Cyrille Dreyfus, Jacob E Corn, Eva-Maria Strauch, Ian A Wilson, and David Baker. Computational design of proteins targeting the conserved stem region of influenza hemagglutinin. *Science*, 332(6031):816–821, 2011.
